# Supplementary material for: Transient telomere uncapping triggers telomeric and subtelomeric rearrangements
Source: EMBO Rep. 2026 Feb 17;27(6):1607–31. doi: 10.1038/s44319-026-00717-4 (PMC13022453; doi:10.1038/s44319-026-00717-4)
Supplement: Supplementary file 1 — Table EV1 [file 44319_2026_717_MOESM1_ESM.docx]

| Strain | Genotype |
| --- | --- |
| yT1291 | MAT**α** *ura3-1 trp1-1 leu2-3,112 his3-11,15 can1-100* ADE2 RAD5 *cdc5::CDC5-3HA-TRP1 cdc13-1* |
| yT1292 | MAT**α** *ura3-1 trp1-1 leu2-3,112 his3-11,15 can1-100* ADE2 RAD5 *cdc5::cdc5-ad-3HA-TRP1* *cdc13-1* |
| yZX409 | yT1291 *rad51::HIS3* |
| yZX419 | yT1291 *rad52::HIS3* |
| yZX424 | yT1291 *rad59::HIS3* |
| yZX447 | yT1291 *pol32::HIS3* |
| yZX500 | yT1291 *mec1-21* |
| yZX503 | yT1291 *rad59::HIS3 rad51::LEU2* |

**Table EV1. Yeast strains used in this study.**
