# Supplementary material for: Transient telomere uncapping triggers telomeric and subtelomeric rearrangements
Source: EMBO Rep. 2026 Feb 17;27(6):1607–31. doi: 10.1038/s44319-026-00717-4 (PMC13022453; doi:10.1038/s44319-026-00717-4)
Supplement: Supplementary file 2 — Table EV2 [file 44319_2026_717_MOESM2_ESM.docx]

| Strain | Chemistry | Bases kept | Read count | Median depth | | N50 | PHRED quality | Nb of contigs | Asssembly size (b) |
| --- | --- | --- | --- | --- | --- | --- | --- | --- | --- |
| yT1291 | SQK-LSK109 | 600M | 23500 | 38 | 27354 | | 15.46 | 16 | 12254979 |
| yT1291_2 | SQK-LSK109 | 700M | 10054 | 51 | 72413 | | 16.39 | 16 | 12374096 |
| yT1291_survivor_c2 | SQK-LSK109 | 600M | 22814 | 35 | 27842 | | 14.05 | 17 | 12381501 |
| yT1291_survivor_c3.1 | SQK-LSK109 | 600M | 23728 | 34 | 26273 | | 14.65 | 17 | 12385410 |
| yT1291_survivor_c3.2 | SQK-LSK109 | 600M | 22788 | 34 | 21138 | | 14.64 | 17 | 12369144 |
| yT1291_survivor_c3.3 | SQK-LSK109 | 600M | 29840 | 29 | 21138 | | 14.15 | 16 | 12399802 |
| yT1291_survivor_c3.4 | SQK-LSK109 | 600M | 36991 | 29 | 16703 | | 14.38 | 20 | 10968454 |
| yT1291_survivor_c5 | SQK-LSK109 | 600M | 23127 | 37 | 27912 | | 14.61 | 17 | 12310970 |
| yT1291_survivor_c17 | SQK-LSK109 | 600M | 22085 | 37 | 28315 | | 14.84 | 17 | 12432499 |
| yT1291_survivor_c20 | SQK-LSK109 | 581M | 42120 | 32 | 20924 | | 13.38 | 17 | 12305791 |
| yT1291_survivor_c21 | SQK-LSK109 | 600M | 30013 | 31 | 22007 | | 14.18 | 16 | 12368767 |
| yT1291_survivor_c26 | SQK-LSK110 | 600M | 13423 | 39 | 43254 | | 21.03 | 19 | 12492133 |
| yT1291_survivor_c27 | SQK-LSK109 | 600M | 39491 | 31 | 20928 | | 13.37 | 17 | 12354973 |
| yT1291_survivor_c29 | SQK-LSK110 | 600M | 6324 | 42 | 93349 | | 24.6 | 18 | 12759246 |
| yZX424 | SQK-LSK110 | 600M | 9428 | 37 | 64933 | | 23.8 | 17 | 12436295 |
| yZX424_survivor_c2 | SQK-LSK110 | 600M | 10639 | 27 | 54440 | | 22.7 | 20 | 11167879 |
| yZX424_survivor_c6 | SQK-LSK110 | 600M | 13517 | 37 | 42869 | | 24.58 | 16 | 12292235 |
| yZX424_survivor_c9 | SQK-LSK110 | 600M | 9748 | 36 | 59303 | | 22.84 | 17 | 12297725 |
| yZX424_survivor_c12 | SQK-LSK110 | 600M | 11500 | 39 | 55698 | | 22.62 | 17 | 12412417 |
| yZX409 | SQK-LSK110 | 600M | 15797 | 32 | 36955 | | 24.08 | 18 | 12492910 |
| yZX409_survivor_c8 | SQK-LSK110 | 600M | 11161 | 40 | 52275 | | 23.75 | 17 | 12483071 |
| yZX409_survivor_c9 | SQK-LSK110 | 600M | 14151 | 34 | 40646 | | 23.25 | 18 | 12669384 |
| yZX409_survivor_c10 | SQK-LSK110 | 600M | 14310 | 32 | 40646 | | 23.25 | 17 | 12379387 |
| yZX409_survivor_c17 | SQK-LSK110 | 600M | 13810 | 34 | 41877 | | 24.1 | 17 | 12473960 |
| yZX419 | SQK-LSK110 | 600M | 13530 | 27 | 42948 | | 20.34 | 18 | 12257221 |
| yZX419_survivor_c3 | SQK-LSK110 | 600M | 11990 | 33 | 47334 | | 23.09 | 19 | 12373752 |
| yZX419_survivor_c8 | SQK-LSK110 | 600M | 8660 | 41 | 67801 | | 22.23 | 17 | 12282927 |
| yZX419_survivor_c11 | SQK-LSK110 | 600M | 9137 | 40 | 62967 | | 20.82 | 17 | 12247306 |
| yZX419_survivor_c15 | SQK-LSK110 | 600M | 8521 | 42 | 68814 | | 22.35 | 17 | 12272940 |
| yZX447 | SQK-LSK110 | 600M | 8909 | 40 | 64933 | | 23.8 | 17 | 12248378 |
| yZX447_survivor_c5 | SQK-LSK110 | 600M | 9901 | 42 | 57975 | | 24.1 | 16 | 12321768 |
| yZX447_survivor_c6 | SQK-LSK110 | 600M | 7967 | 42 | 71286 | | 24.71 | 16 | 12330222 |
| yZX447_survivor_c13 | SQK-LSK110 | 600M | 10467 | 42 | 55385 | | 23.97 | 17 | 12351550 |
| yZX447_survivor_c16 | SQK-LSK110 | 600M | 12623 | 39 | 46147 | | 20.79 | 17 | 12285666 |
| yZX503 | SQK-LSK110 | 600M | 10706 | 39 | 55698 | | 22.91 | 17 | 12535522 |
| yZX503_survivor_c2 | SQK-LSK110 | 600M | 9242 | 38 | 64338 | | 23.11 | 17 | 12359884 |
| yZX503_survivor_c7 | SQK-LSK110 | 600M | 11479 | 38 | 53107 | | 22.63 | 18 | 12562493 |
| yZX503_survivor_c14 | SQK-LSK110 | 600M | 8335 | 40 | 70524 | | 23.53 | 17 | 12425479 |
| yZX503_survivor_c15 | SQK-LSK110 | 600M | 8177 | 40 | 71656 | | 23.38 | 17 | 12382989 |

**Table EV2. Sequencing and assembly statistics.**

“Chemistry” indicates the type of flowcell and reagent kits used. “Bases kept” indicates the number of bases used for assembly after filtering. “Read count” indicates the number of reads used for assembly after filtering. “Median depth” indicates the median depth of filtered reads mapped to the final assembly. “N50” corresponds to the N50 of the reads used for assembly. “PHREd quality” is the average PHRED quality score of the reads used for assembly. “Nb of contigs” is the final number of contigs in assemblies, excluding mitochondrial contigs.
