# Supplementary material for: Transient telomere uncapping triggers telomeric and subtelomeric rearrangements
Source: EMBO Rep. 2026 Feb 17;27(6):1607–31. doi: 10.1038/s44319-026-00717-4 (PMC13022453; doi:10.1038/s44319-026-00717-4)
Supplement: Supplementary file 4 — Dataset EV1 [file 44319_2026_717_MOESM4_ESM.zip › Dataset EV1 Legend.rtf]

Data EV1. All Y’ elements in the genome assembly of the control cdc13-1 strain.
