# Supplementary material for: Transient telomere uncapping triggers telomeric and subtelomeric rearrangements
Source: EMBO Rep. 2026 Feb 17;27(6):1607–31. doi: 10.1038/s44319-026-00717-4 (PMC13022453; doi:10.1038/s44319-026-00717-4)
Supplement: Supplementary file 5 — Dataset EV2 [file 44319_2026_717_MOESM5_ESM.zip › Dataset EV2/Dataset EV2 Legend.rtf]

Dataset EV2. Representation as in Fig. 3D of all extremities across all sequenced clones in all strains (one per folder). The legend of each sample is shown on the representation of Chromosome I left.
