# Supplementary figures and images for: Transient telomere uncapping triggers telomeric and subtelomeric rearrangements (part 2 of 3)
Source: EMBO Rep. 2026 Feb 17;27(6):1607–31. doi: 10.1038/s44319-026-00717-4 (PMC13022453; doi:10.1038/s44319-026-00717-4)

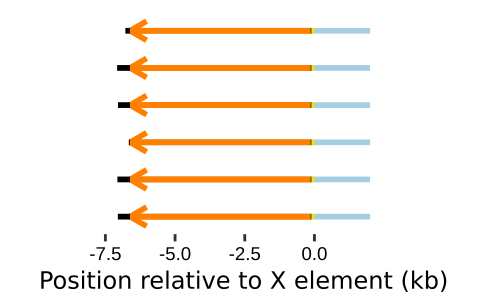

Supplement: Supplementary file 5 — Dataset EV2 [file 44319_2026_717_MOESM5_ESM.zip › Dataset EV2/rad51_rad59/Chr_V.left.png]

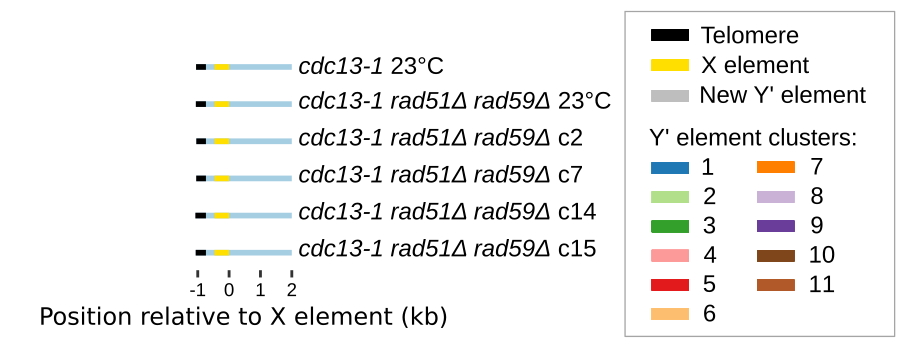

Supplement: Supplementary file 5 — Dataset EV2 [file 44319_2026_717_MOESM5_ESM.zip › Dataset EV2/rad51_rad59/Chr_I.left.png]

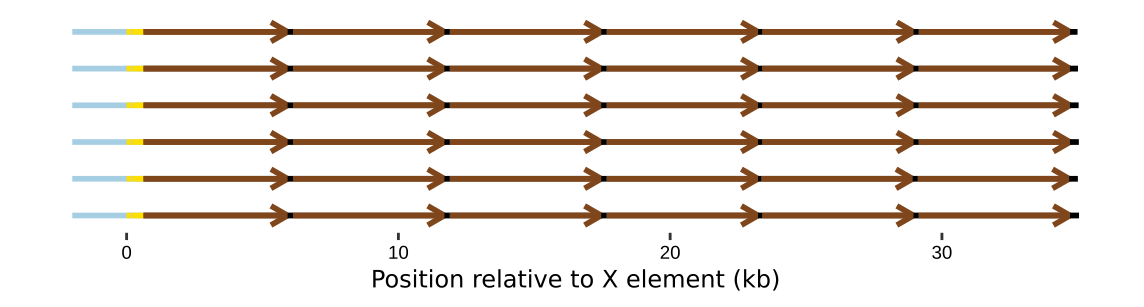

Supplement: Supplementary file 5 — Dataset EV2 [file 44319_2026_717_MOESM5_ESM.zip › Dataset EV2/rad51_rad59/Chr_XVI.right.png]

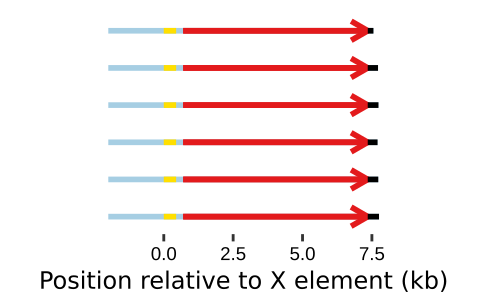

Supplement: Supplementary file 5 — Dataset EV2 [file 44319_2026_717_MOESM5_ESM.zip › Dataset EV2/rad51_rad59/Chr_XV.right.png]

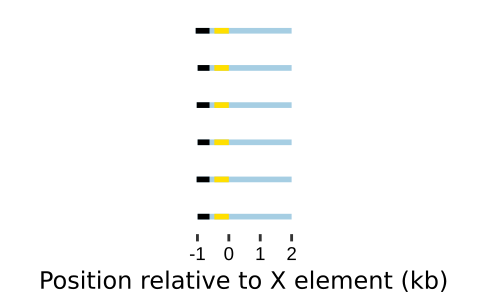

Supplement: Supplementary file 5 — Dataset EV2 [file 44319_2026_717_MOESM5_ESM.zip › Dataset EV2/rad51_rad59/Chr_VII.left.png]

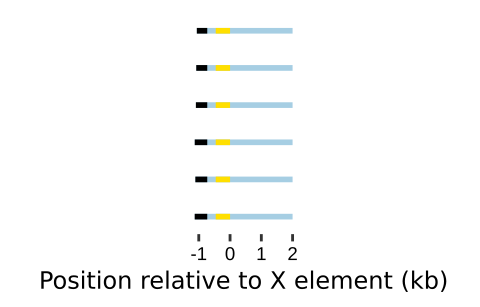

Supplement: Supplementary file 5 — Dataset EV2 [file 44319_2026_717_MOESM5_ESM.zip › Dataset EV2/rad51_rad59/Chr_XV.left.png]

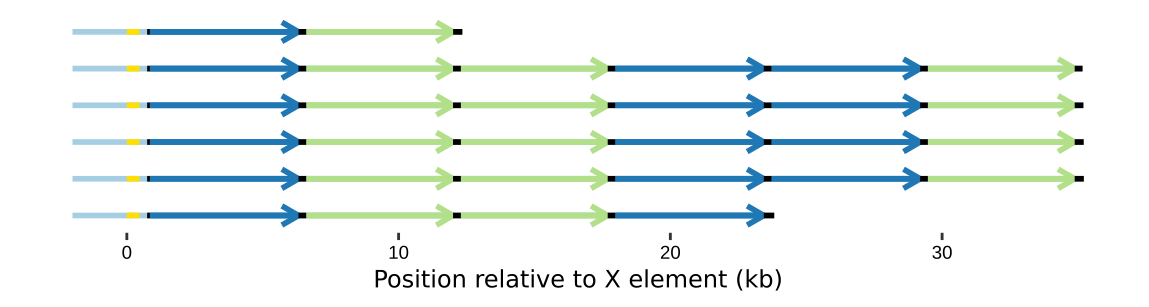

Supplement: Supplementary file 5 — Dataset EV2 [file 44319_2026_717_MOESM5_ESM.zip › Dataset EV2/rad51_rad59/Chr_I.right.png]

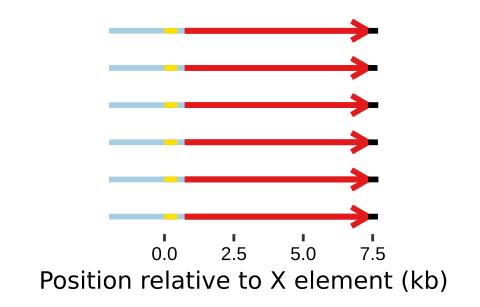

Supplement: Supplementary file 5 — Dataset EV2 [file 44319_2026_717_MOESM5_ESM.zip › Dataset EV2/rad51_rad59/Chr_V.right.png]

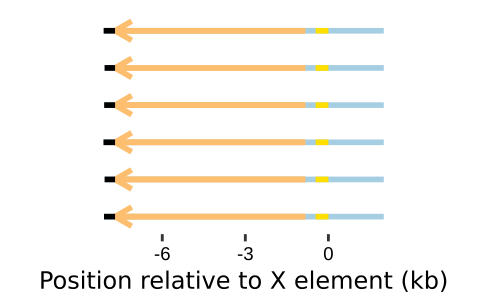

Supplement: Supplementary file 5 — Dataset EV2 [file 44319_2026_717_MOESM5_ESM.zip › Dataset EV2/rad51_rad59/Chr_IX.left.png]

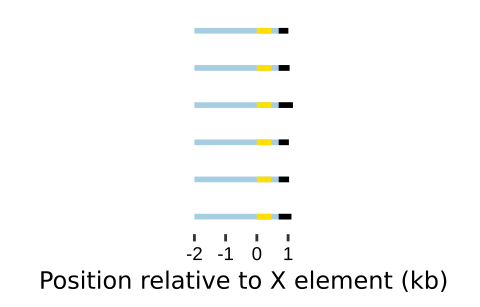

Supplement: Supplementary file 5 — Dataset EV2 [file 44319_2026_717_MOESM5_ESM.zip › Dataset EV2/rad51_rad59/Chr_XI.right.png]

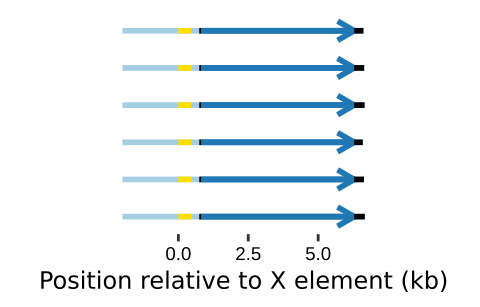

Supplement: Supplementary file 5 — Dataset EV2 [file 44319_2026_717_MOESM5_ESM.zip › Dataset EV2/rad51_rad59/Chr_III.right.png]

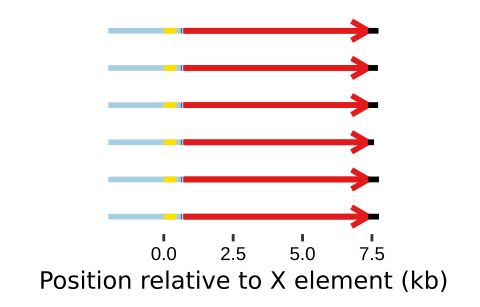

Supplement: Supplementary file 5 — Dataset EV2 [file 44319_2026_717_MOESM5_ESM.zip › Dataset EV2/rad51_rad59/Chr_VII.right.png]

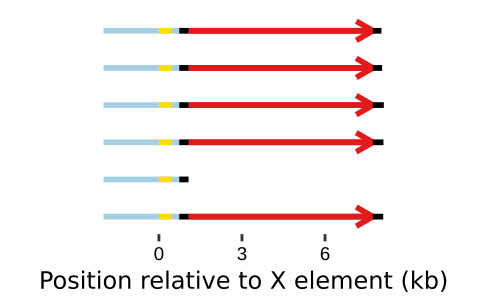

Supplement: Supplementary file 5 — Dataset EV2 [file 44319_2026_717_MOESM5_ESM.zip › Dataset EV2/rad51_rad59/Chr_IV.right.png]

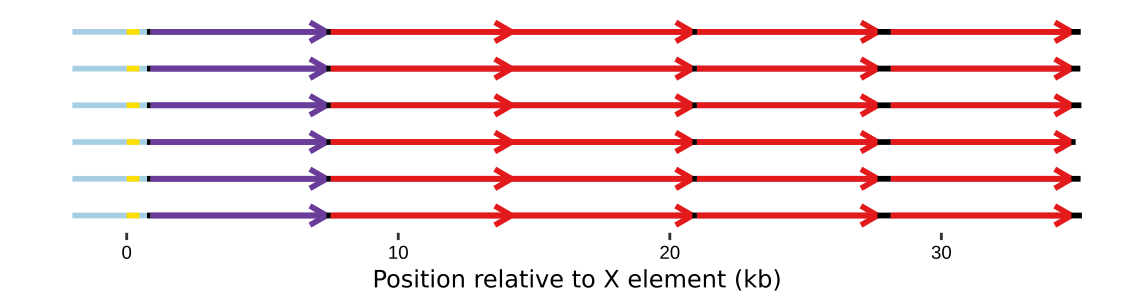

Supplement: Supplementary file 5 — Dataset EV2 [file 44319_2026_717_MOESM5_ESM.zip › Dataset EV2/rad51_rad59/Chr_XII.right.png]

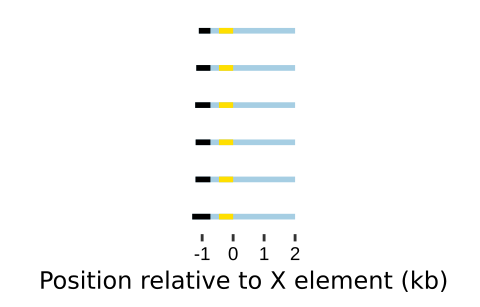

Supplement: Supplementary file 5 — Dataset EV2 [file 44319_2026_717_MOESM5_ESM.zip › Dataset EV2/rad51_rad59/Chr_III.left.png]

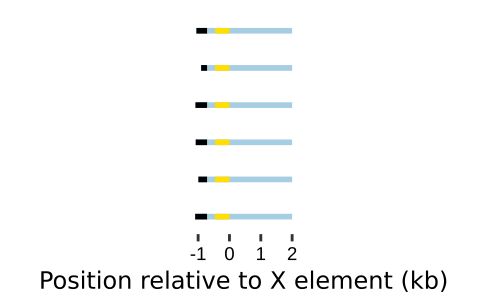

Supplement: Supplementary file 5 — Dataset EV2 [file 44319_2026_717_MOESM5_ESM.zip › Dataset EV2/rad51_rad59/Chr_IV.left.png]

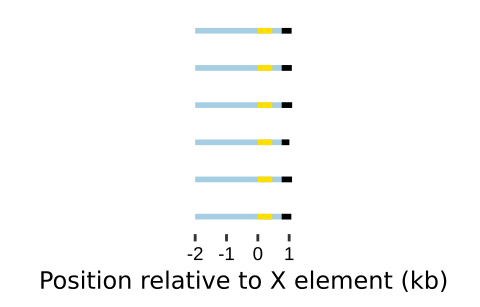

Supplement: Supplementary file 5 — Dataset EV2 [file 44319_2026_717_MOESM5_ESM.zip › Dataset EV2/rad51_rad59/Chr_X.right.png]

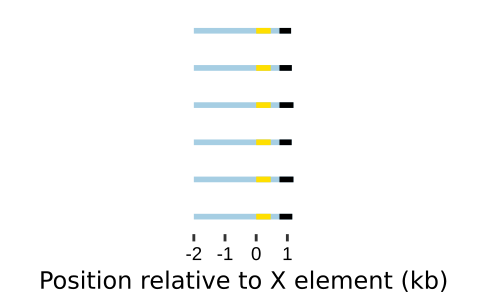

Supplement: Supplementary file 5 — Dataset EV2 [file 44319_2026_717_MOESM5_ESM.zip › Dataset EV2/rad51_rad59/Chr_IX.right.png]

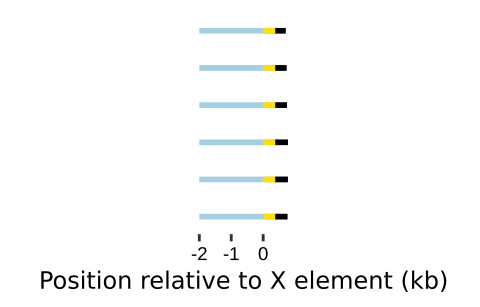

Supplement: Supplementary file 5 — Dataset EV2 [file 44319_2026_717_MOESM5_ESM.zip › Dataset EV2/rad51_rad59/Chr_VI.right.png]

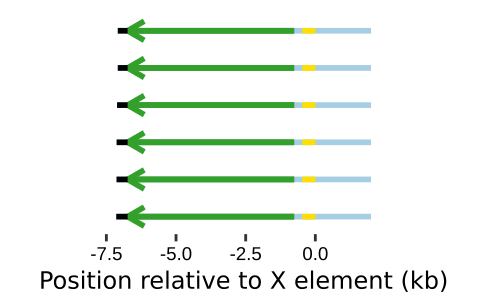

Supplement: Supplementary file 5 — Dataset EV2 [file 44319_2026_717_MOESM5_ESM.zip › Dataset EV2/rad51_rad59/Chr_II.left.png]

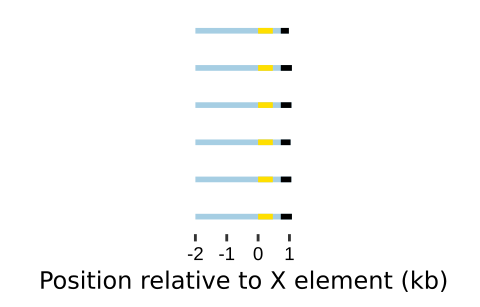

Supplement: Supplementary file 5 — Dataset EV2 [file 44319_2026_717_MOESM5_ESM.zip › Dataset EV2/rad51_rad59/Chr_VIII.right.png]

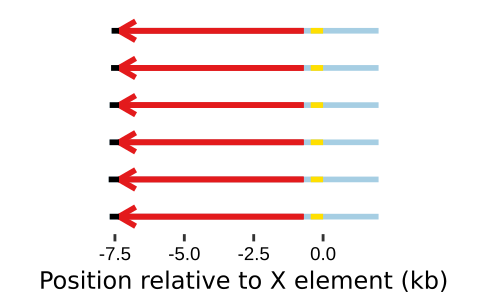

Supplement: Supplementary file 5 — Dataset EV2 [file 44319_2026_717_MOESM5_ESM.zip › Dataset EV2/rad51_rad59/Chr_XVI.left.png]

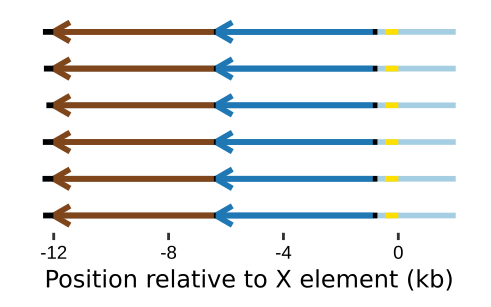

Supplement: Supplementary file 5 — Dataset EV2 [file 44319_2026_717_MOESM5_ESM.zip › Dataset EV2/rad51_rad59/Chr_XII.left.png]

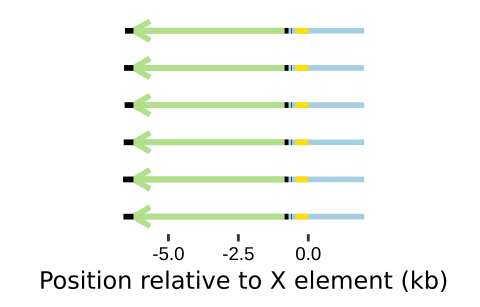

Supplement: Supplementary file 5 — Dataset EV2 [file 44319_2026_717_MOESM5_ESM.zip › Dataset EV2/rad51_rad59/Chr_VI.left.png]

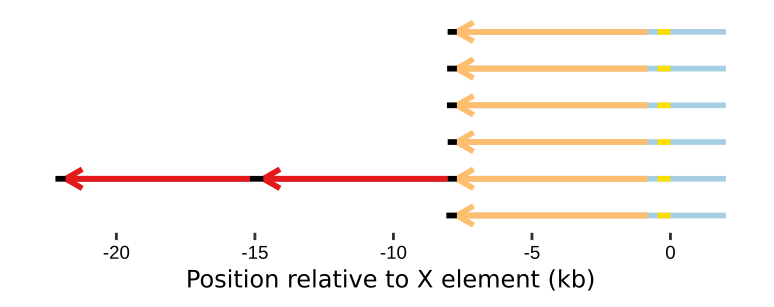

Supplement: Supplementary file 5 — Dataset EV2 [file 44319_2026_717_MOESM5_ESM.zip › Dataset EV2/rad51_rad59/Chr_X.left.png]

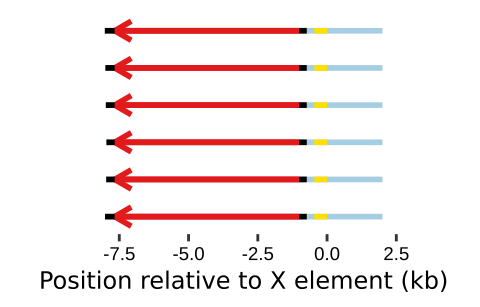

Supplement: Supplementary file 5 — Dataset EV2 [file 44319_2026_717_MOESM5_ESM.zip › Dataset EV2/rad51_rad59/Chr_XIV.left.png]

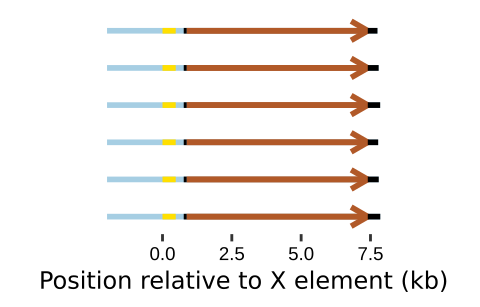

Supplement: Supplementary file 5 — Dataset EV2 [file 44319_2026_717_MOESM5_ESM.zip › Dataset EV2/rad51_rad59/Chr_XIV.right.png]

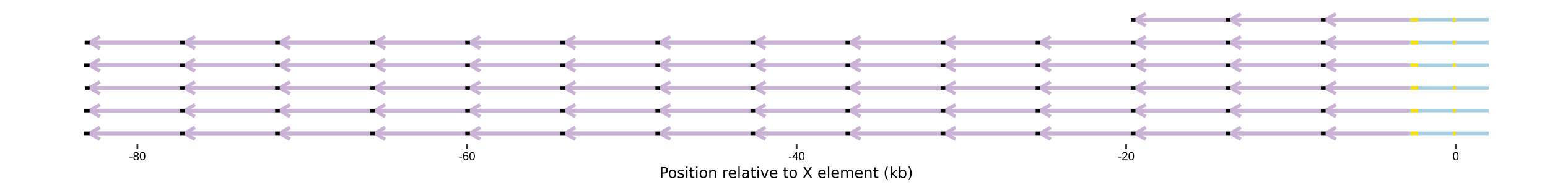

Supplement: Supplementary file 5 — Dataset EV2 [file 44319_2026_717_MOESM5_ESM.zip › Dataset EV2/rad51_rad59/Chr_VIII.left.png]

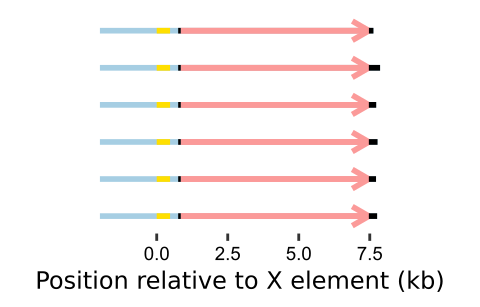

Supplement: Supplementary file 5 — Dataset EV2 [file 44319_2026_717_MOESM5_ESM.zip › Dataset EV2/rad52/Chr_II.right.png]

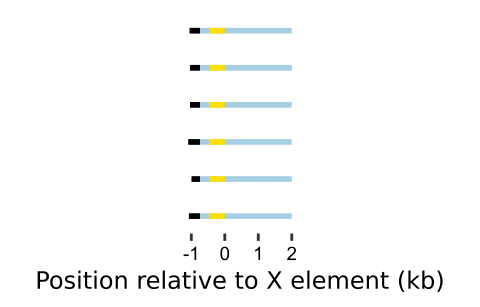

Supplement: Supplementary file 5 — Dataset EV2 [file 44319_2026_717_MOESM5_ESM.zip › Dataset EV2/rad52/Chr_XI.left.png]

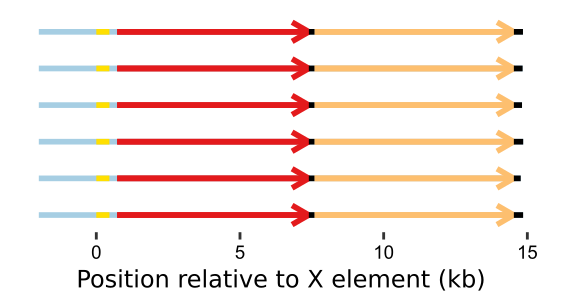

Supplement: Supplementary file 5 — Dataset EV2 [file 44319_2026_717_MOESM5_ESM.zip › Dataset EV2/rad52/Chr_XIII.right.png]

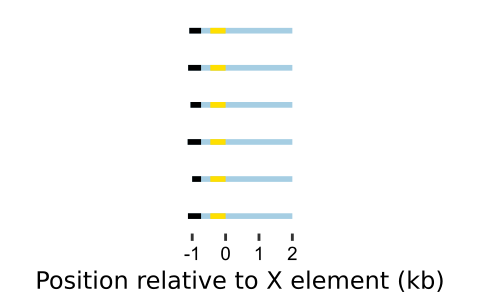

Supplement: Supplementary file 5 — Dataset EV2 [file 44319_2026_717_MOESM5_ESM.zip › Dataset EV2/rad52/Chr_XIII.left.png]

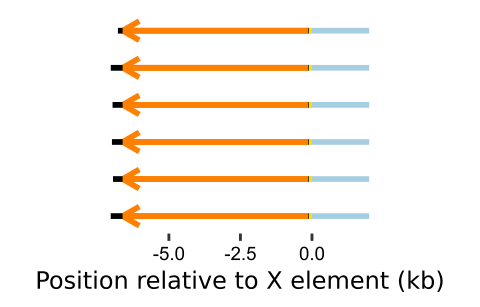

Supplement: Supplementary file 5 — Dataset EV2 [file 44319_2026_717_MOESM5_ESM.zip › Dataset EV2/rad52/Chr_V.left.png]

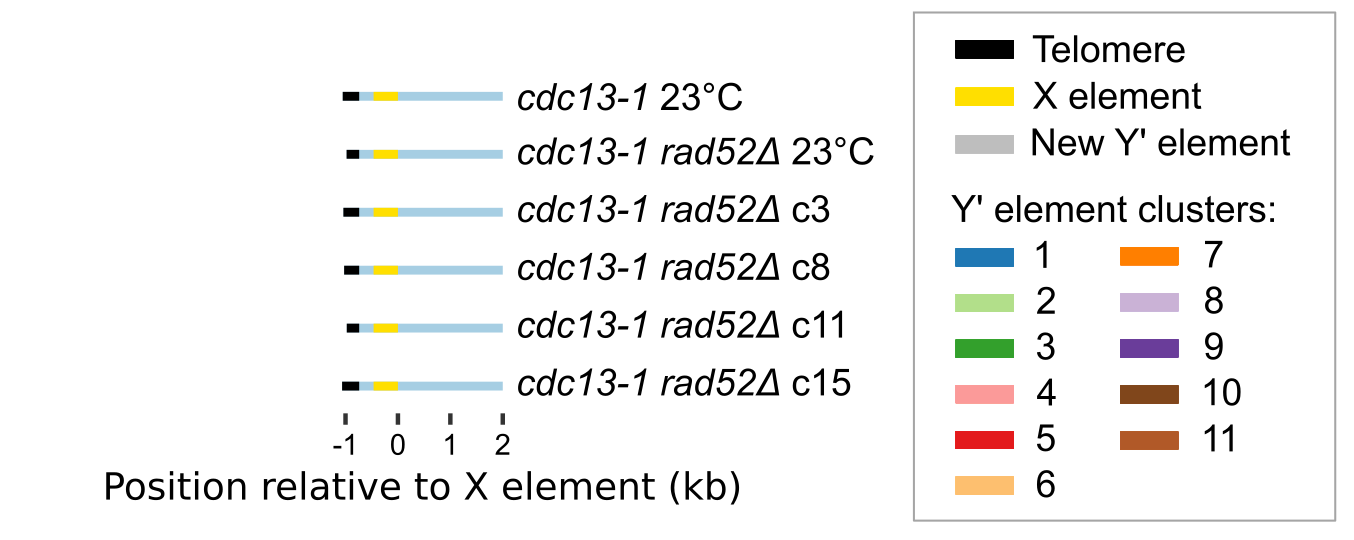

Supplement: Supplementary file 5 — Dataset EV2 [file 44319_2026_717_MOESM5_ESM.zip › Dataset EV2/rad52/Chr_I.left.png]

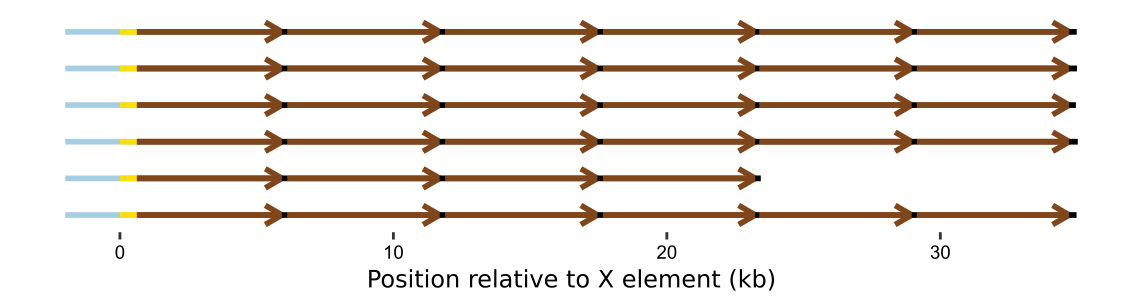

Supplement: Supplementary file 5 — Dataset EV2 [file 44319_2026_717_MOESM5_ESM.zip › Dataset EV2/rad52/Chr_XVI.right.png]

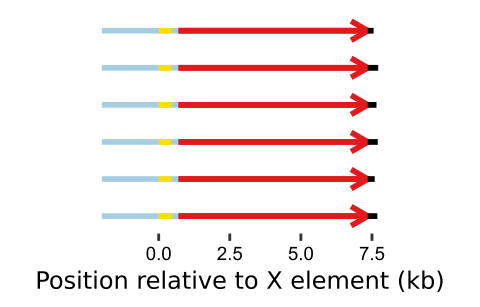

Supplement: Supplementary file 5 — Dataset EV2 [file 44319_2026_717_MOESM5_ESM.zip › Dataset EV2/rad52/Chr_XV.right.png]

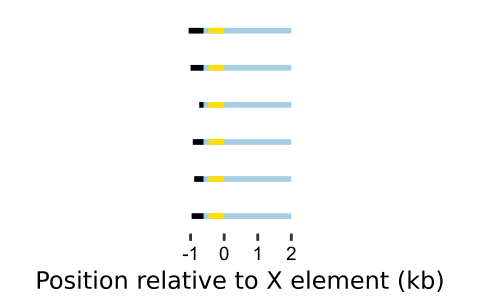

Supplement: Supplementary file 5 — Dataset EV2 [file 44319_2026_717_MOESM5_ESM.zip › Dataset EV2/rad52/Chr_VII.left.png]

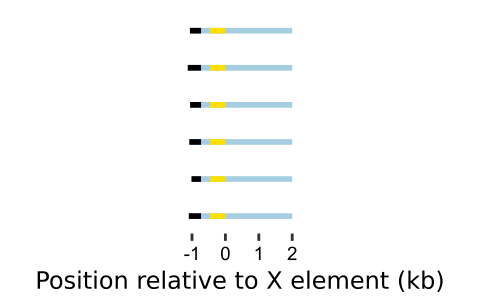

Supplement: Supplementary file 5 — Dataset EV2 [file 44319_2026_717_MOESM5_ESM.zip › Dataset EV2/rad52/Chr_XV.left.png]

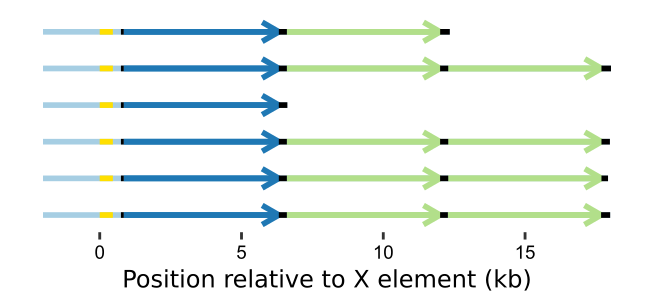

Supplement: Supplementary file 5 — Dataset EV2 [file 44319_2026_717_MOESM5_ESM.zip › Dataset EV2/rad52/Chr_I.right.png]

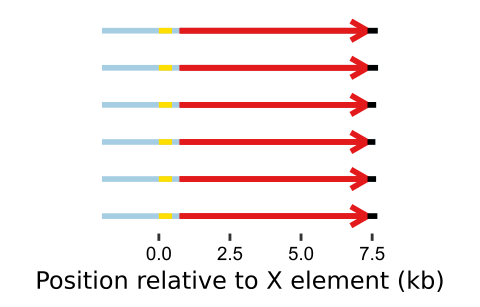

Supplement: Supplementary file 5 — Dataset EV2 [file 44319_2026_717_MOESM5_ESM.zip › Dataset EV2/rad52/Chr_V.right.png]

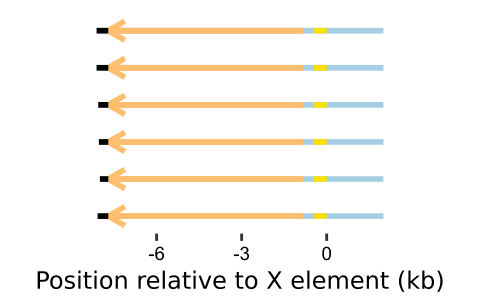

Supplement: Supplementary file 5 — Dataset EV2 [file 44319_2026_717_MOESM5_ESM.zip › Dataset EV2/rad52/Chr_IX.left.png]

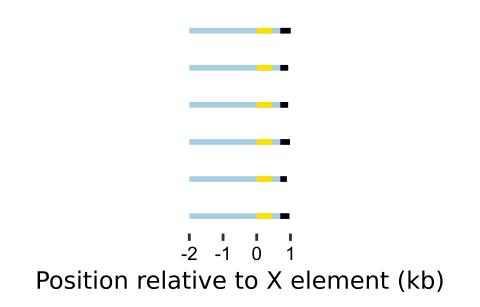

Supplement: Supplementary file 5 — Dataset EV2 [file 44319_2026_717_MOESM5_ESM.zip › Dataset EV2/rad52/Chr_XI.right.png]

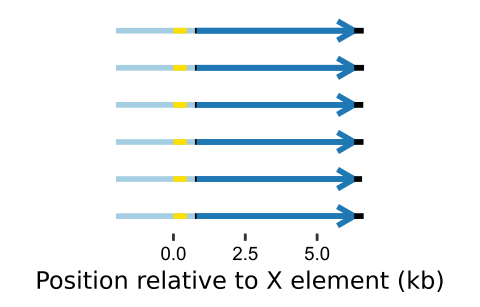

Supplement: Supplementary file 5 — Dataset EV2 [file 44319_2026_717_MOESM5_ESM.zip › Dataset EV2/rad52/Chr_III.right.png]

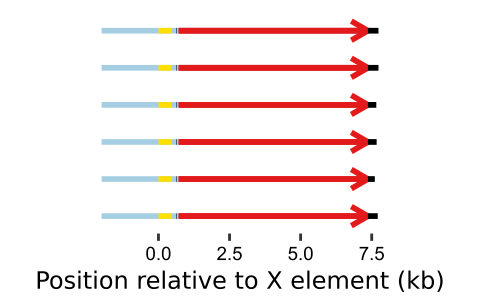

Supplement: Supplementary file 5 — Dataset EV2 [file 44319_2026_717_MOESM5_ESM.zip › Dataset EV2/rad52/Chr_VII.right.png]

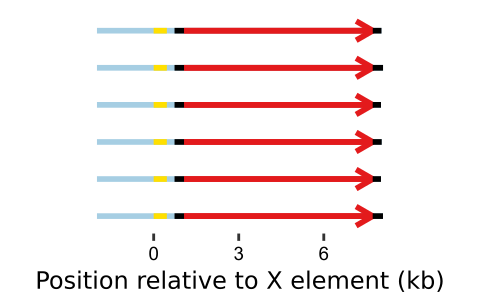

Supplement: Supplementary file 5 — Dataset EV2 [file 44319_2026_717_MOESM5_ESM.zip › Dataset EV2/rad52/Chr_IV.right.png]

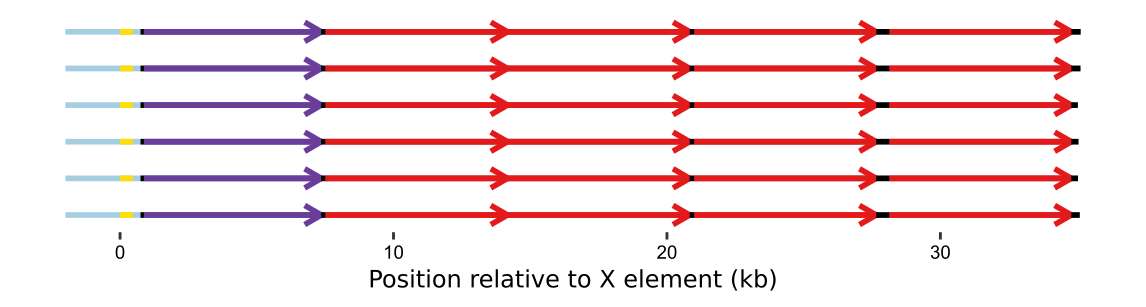

Supplement: Supplementary file 5 — Dataset EV2 [file 44319_2026_717_MOESM5_ESM.zip › Dataset EV2/rad52/Chr_XII.right.png]

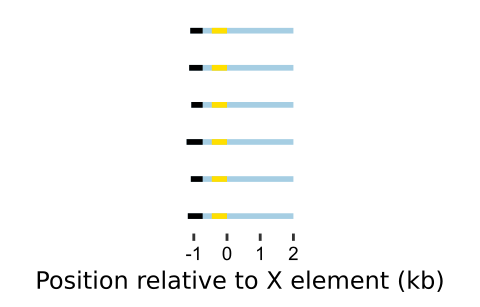

Supplement: Supplementary file 5 — Dataset EV2 [file 44319_2026_717_MOESM5_ESM.zip › Dataset EV2/rad52/Chr_III.left.png]

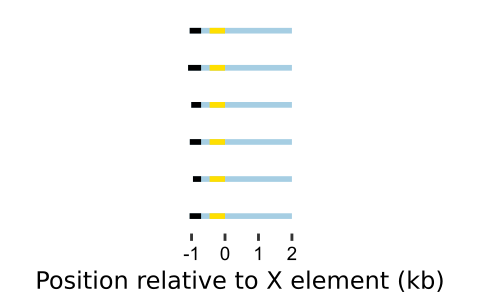

Supplement: Supplementary file 5 — Dataset EV2 [file 44319_2026_717_MOESM5_ESM.zip › Dataset EV2/rad52/Chr_IV.left.png]

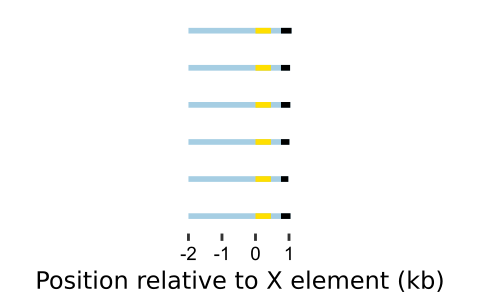

Supplement: Supplementary file 5 — Dataset EV2 [file 44319_2026_717_MOESM5_ESM.zip › Dataset EV2/rad52/Chr_X.right.png]

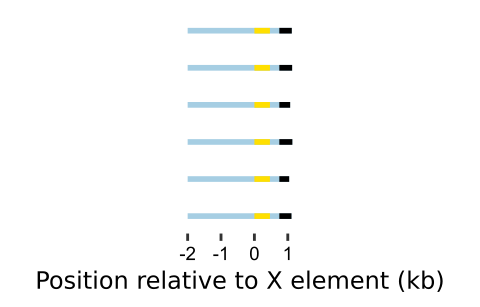

Supplement: Supplementary file 5 — Dataset EV2 [file 44319_2026_717_MOESM5_ESM.zip › Dataset EV2/rad52/Chr_IX.right.png]

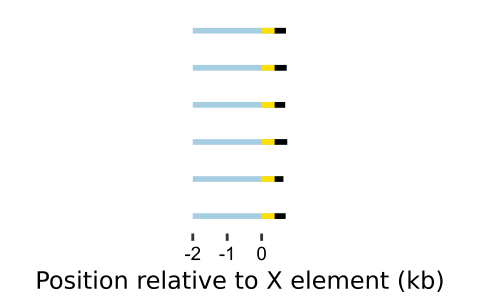

Supplement: Supplementary file 5 — Dataset EV2 [file 44319_2026_717_MOESM5_ESM.zip › Dataset EV2/rad52/Chr_VI.right.png]

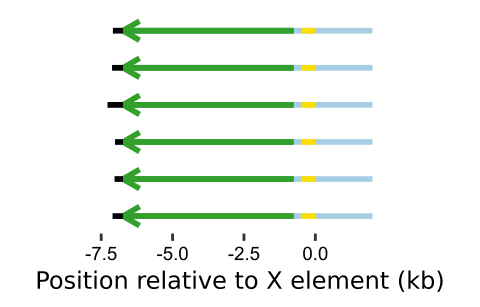

Supplement: Supplementary file 5 — Dataset EV2 [file 44319_2026_717_MOESM5_ESM.zip › Dataset EV2/rad52/Chr_II.left.png]

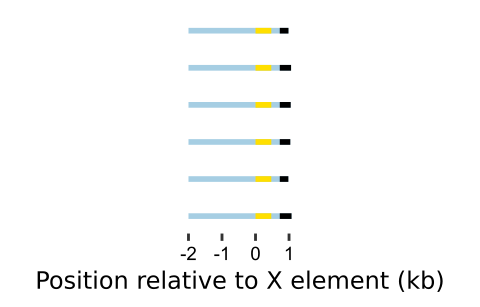

Supplement: Supplementary file 5 — Dataset EV2 [file 44319_2026_717_MOESM5_ESM.zip › Dataset EV2/rad52/Chr_VIII.right.png]

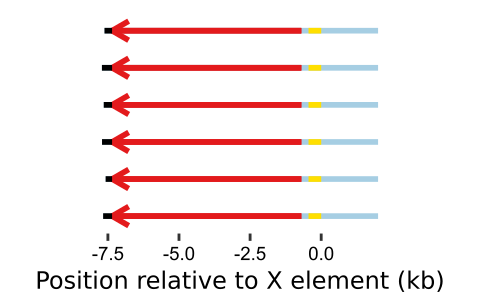

Supplement: Supplementary file 5 — Dataset EV2 [file 44319_2026_717_MOESM5_ESM.zip › Dataset EV2/rad52/Chr_XVI.left.png]

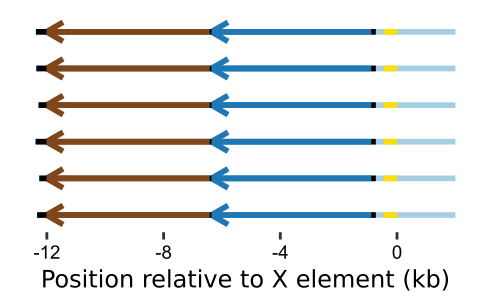

Supplement: Supplementary file 5 — Dataset EV2 [file 44319_2026_717_MOESM5_ESM.zip › Dataset EV2/rad52/Chr_XII.left.png]

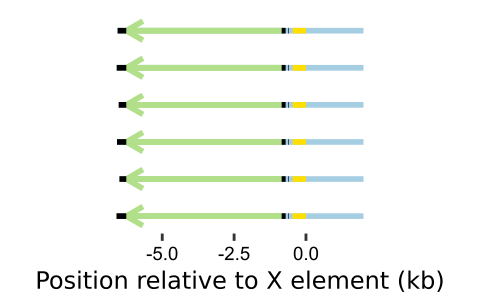

Supplement: Supplementary file 5 — Dataset EV2 [file 44319_2026_717_MOESM5_ESM.zip › Dataset EV2/rad52/Chr_VI.left.png]

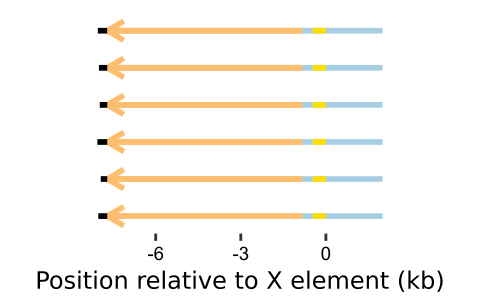

Supplement: Supplementary file 5 — Dataset EV2 [file 44319_2026_717_MOESM5_ESM.zip › Dataset EV2/rad52/Chr_X.left.png]

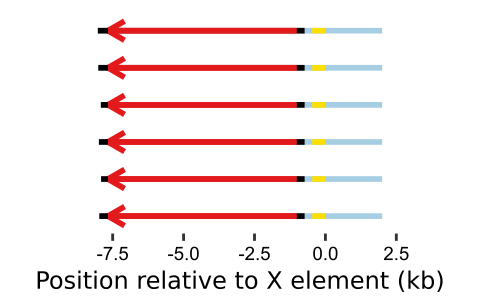

Supplement: Supplementary file 5 — Dataset EV2 [file 44319_2026_717_MOESM5_ESM.zip › Dataset EV2/rad52/Chr_XIV.left.png]

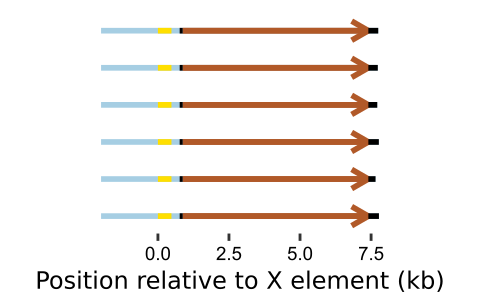

Supplement: Supplementary file 5 — Dataset EV2 [file 44319_2026_717_MOESM5_ESM.zip › Dataset EV2/rad52/Chr_XIV.right.png]

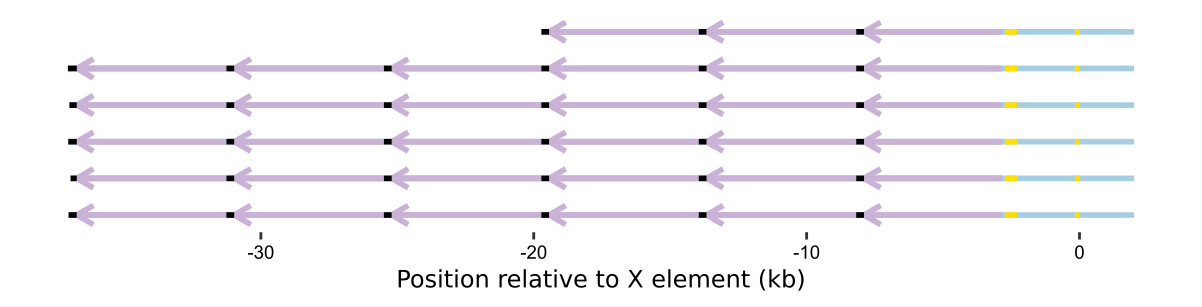

Supplement: Supplementary file 5 — Dataset EV2 [file 44319_2026_717_MOESM5_ESM.zip › Dataset EV2/rad52/Chr_VIII.left.png]

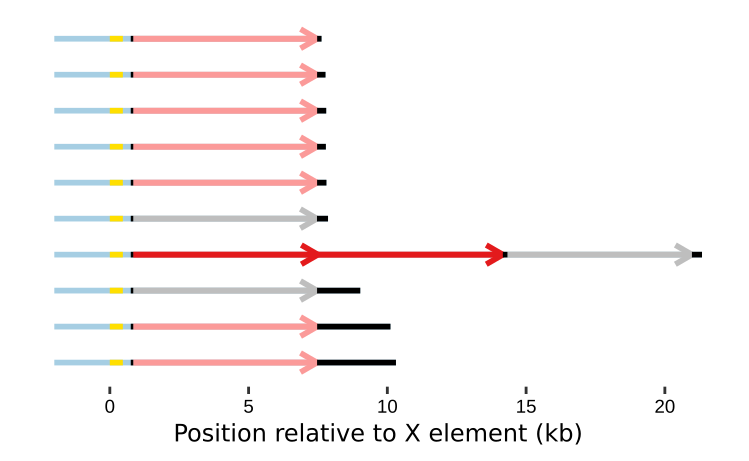

Supplement: Supplementary file 5 — Dataset EV2 [file 44319_2026_717_MOESM5_ESM.zip › Dataset EV2/WT/Chr_II.right.png]

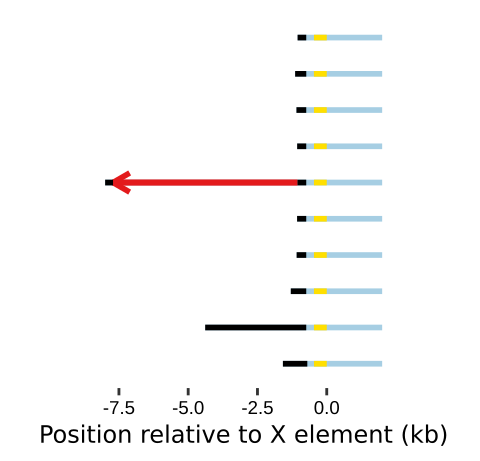

Supplement: Supplementary file 5 — Dataset EV2 [file 44319_2026_717_MOESM5_ESM.zip › Dataset EV2/WT/Chr_XI.left.png]

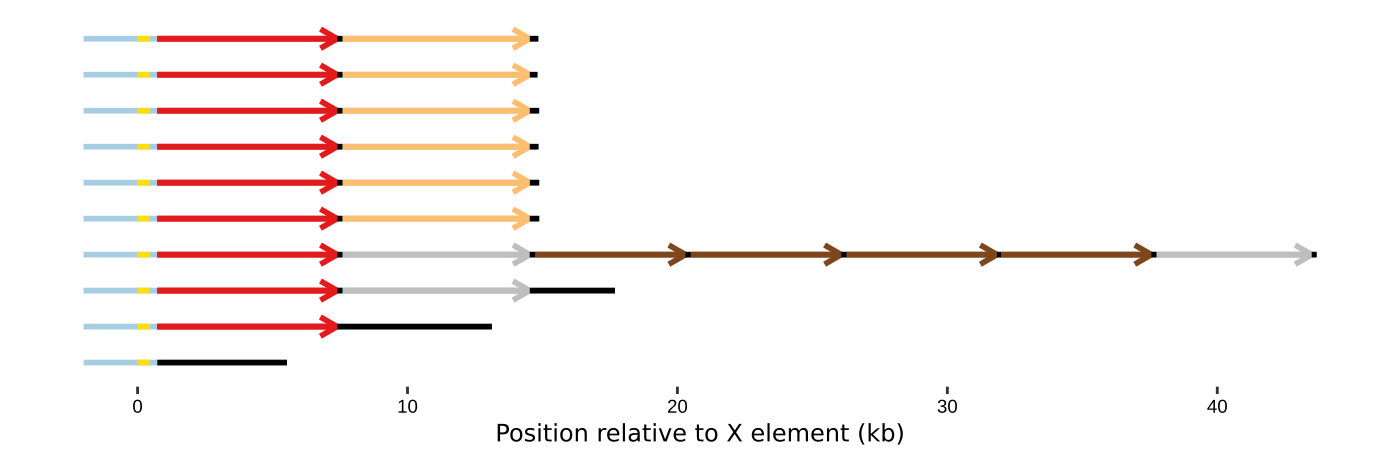

Supplement: Supplementary file 5 — Dataset EV2 [file 44319_2026_717_MOESM5_ESM.zip › Dataset EV2/WT/Chr_XIII.right.png]

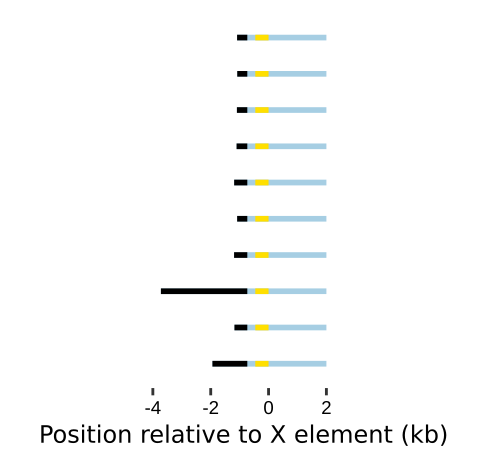

Supplement: Supplementary file 5 — Dataset EV2 [file 44319_2026_717_MOESM5_ESM.zip › Dataset EV2/WT/Chr_XIII.left.png]

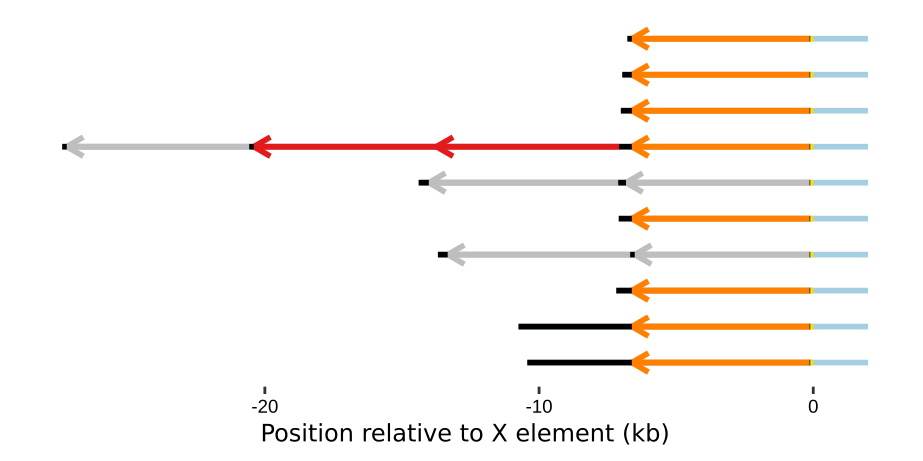

Supplement: Supplementary file 5 — Dataset EV2 [file 44319_2026_717_MOESM5_ESM.zip › Dataset EV2/WT/Chr_V.left.png]

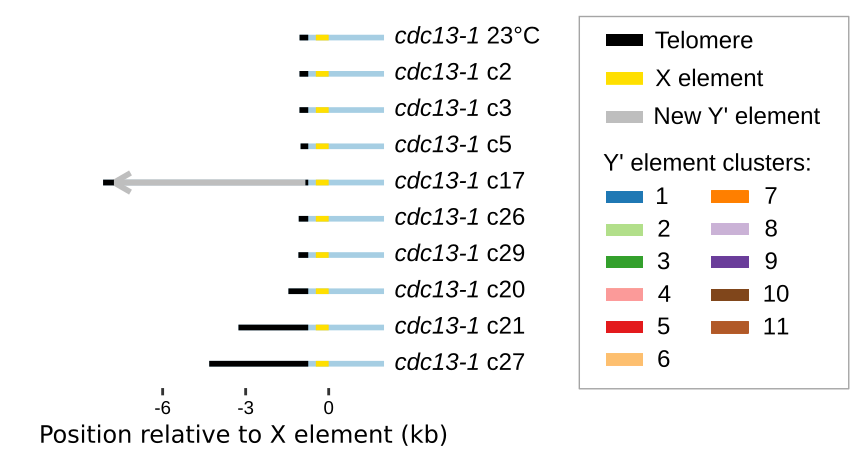

Supplement: Supplementary file 5 — Dataset EV2 [file 44319_2026_717_MOESM5_ESM.zip › Dataset EV2/WT/Chr_I.left.png]

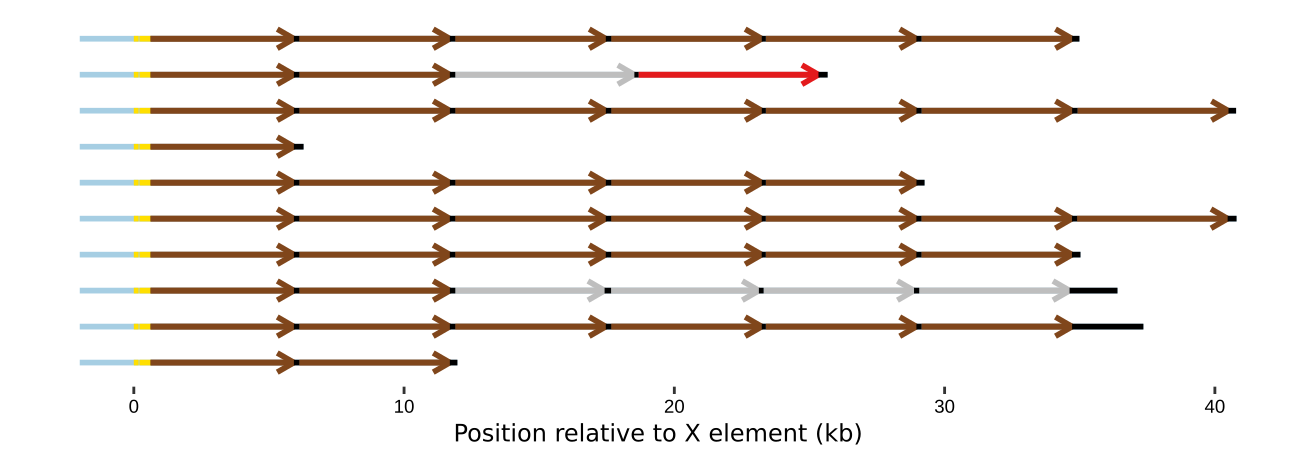

Supplement: Supplementary file 5 — Dataset EV2 [file 44319_2026_717_MOESM5_ESM.zip › Dataset EV2/WT/Chr_XVI.right.png]

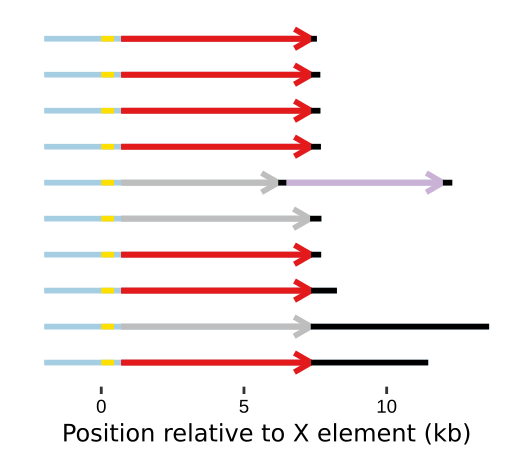

Supplement: Supplementary file 5 — Dataset EV2 [file 44319_2026_717_MOESM5_ESM.zip › Dataset EV2/WT/Chr_XV.right.png]

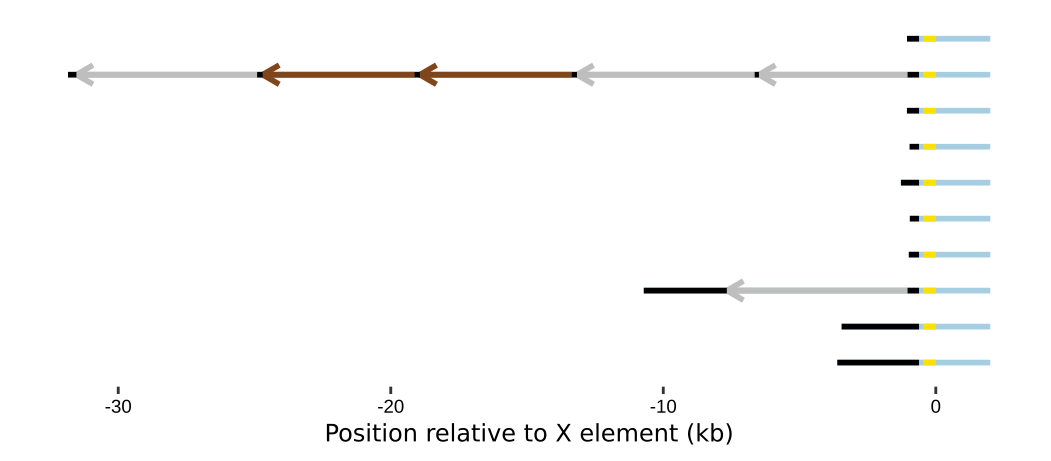

Supplement: Supplementary file 5 — Dataset EV2 [file 44319_2026_717_MOESM5_ESM.zip › Dataset EV2/WT/Chr_VII.left.png]

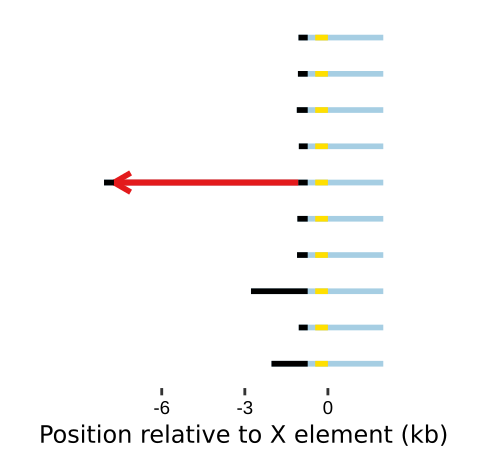

Supplement: Supplementary file 5 — Dataset EV2 [file 44319_2026_717_MOESM5_ESM.zip › Dataset EV2/WT/Chr_XV.left.png]

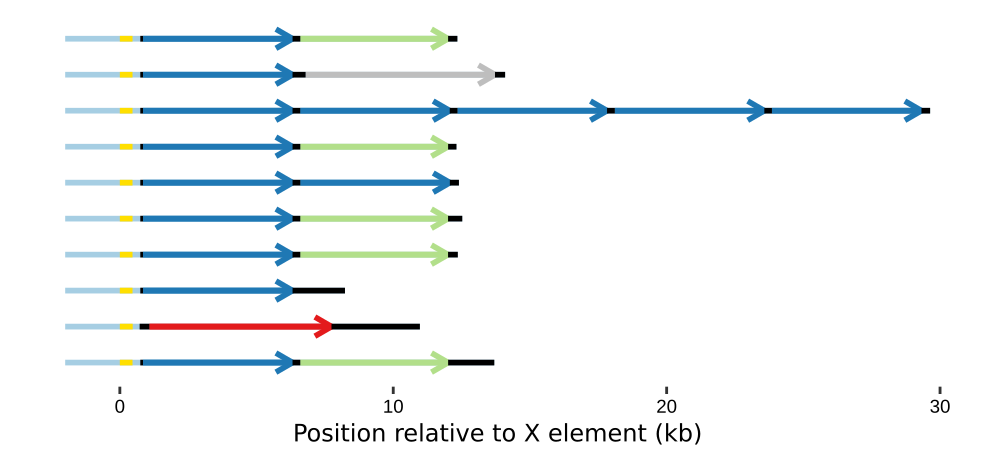

Supplement: Supplementary file 5 — Dataset EV2 [file 44319_2026_717_MOESM5_ESM.zip › Dataset EV2/WT/Chr_I.right.png]

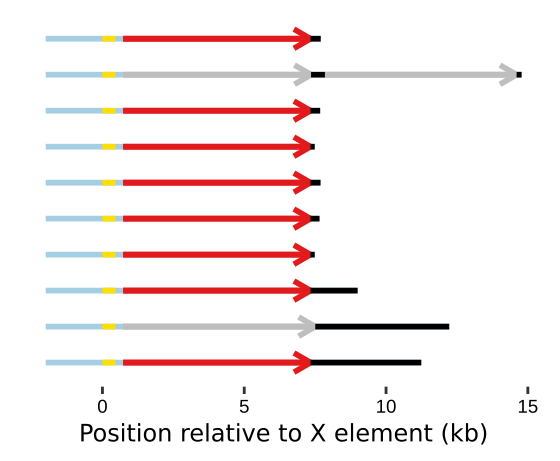

Supplement: Supplementary file 5 — Dataset EV2 [file 44319_2026_717_MOESM5_ESM.zip › Dataset EV2/WT/Chr_V.right.png]

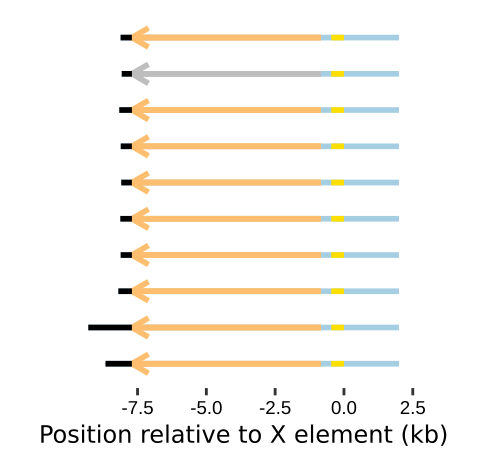

Supplement: Supplementary file 5 — Dataset EV2 [file 44319_2026_717_MOESM5_ESM.zip › Dataset EV2/WT/Chr_IX.left.png]

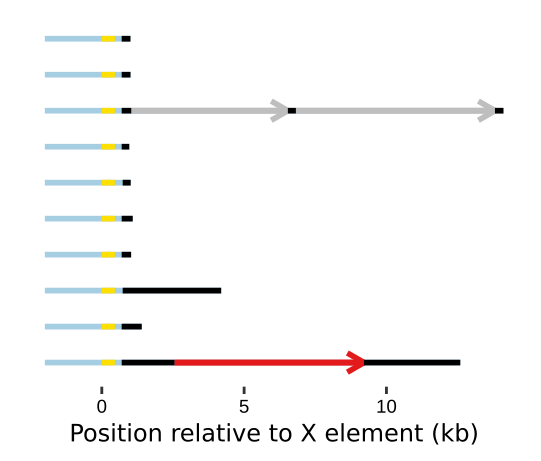

Supplement: Supplementary file 5 — Dataset EV2 [file 44319_2026_717_MOESM5_ESM.zip › Dataset EV2/WT/Chr_XI.right.png]

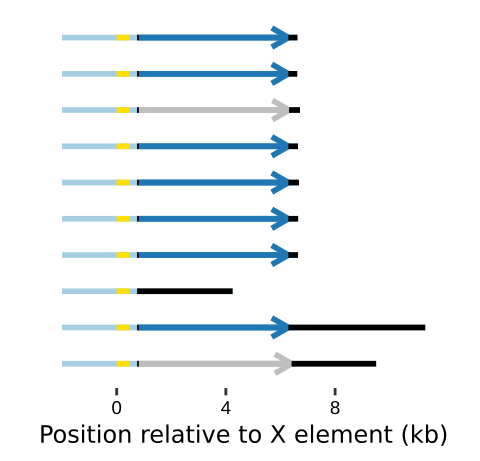

Supplement: Supplementary file 5 — Dataset EV2 [file 44319_2026_717_MOESM5_ESM.zip › Dataset EV2/WT/Chr_III.right.png]

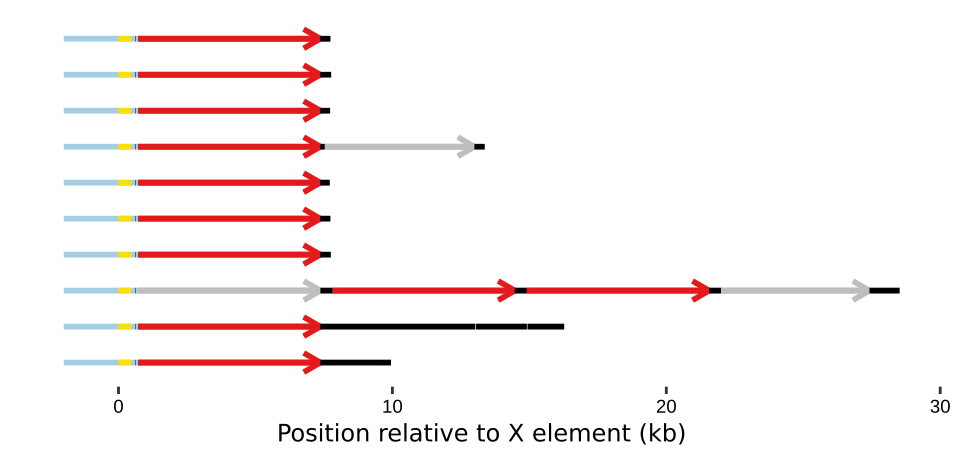

Supplement: Supplementary file 5 — Dataset EV2 [file 44319_2026_717_MOESM5_ESM.zip › Dataset EV2/WT/Chr_VII.right.png]

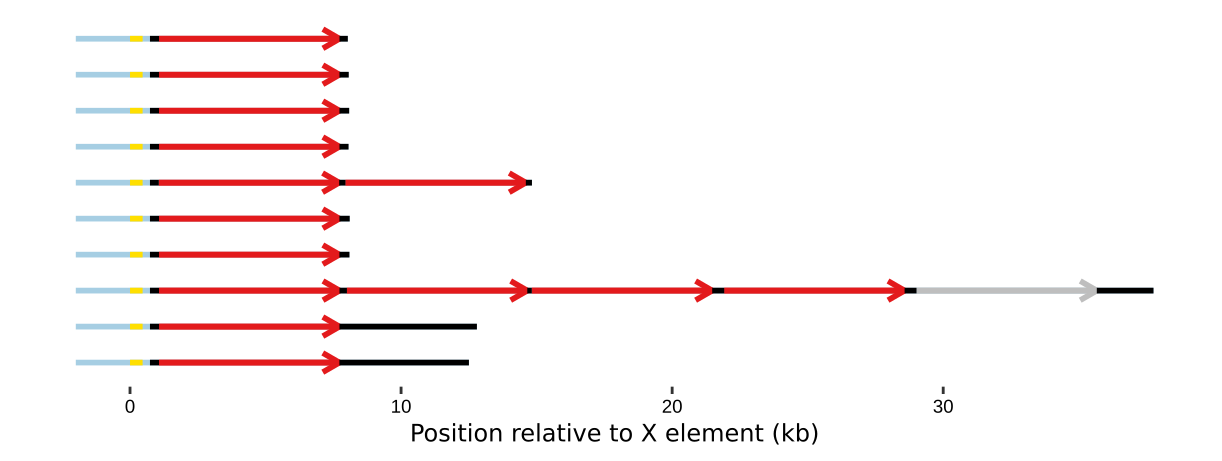

Supplement: Supplementary file 5 — Dataset EV2 [file 44319_2026_717_MOESM5_ESM.zip › Dataset EV2/WT/Chr_IV.right.png]

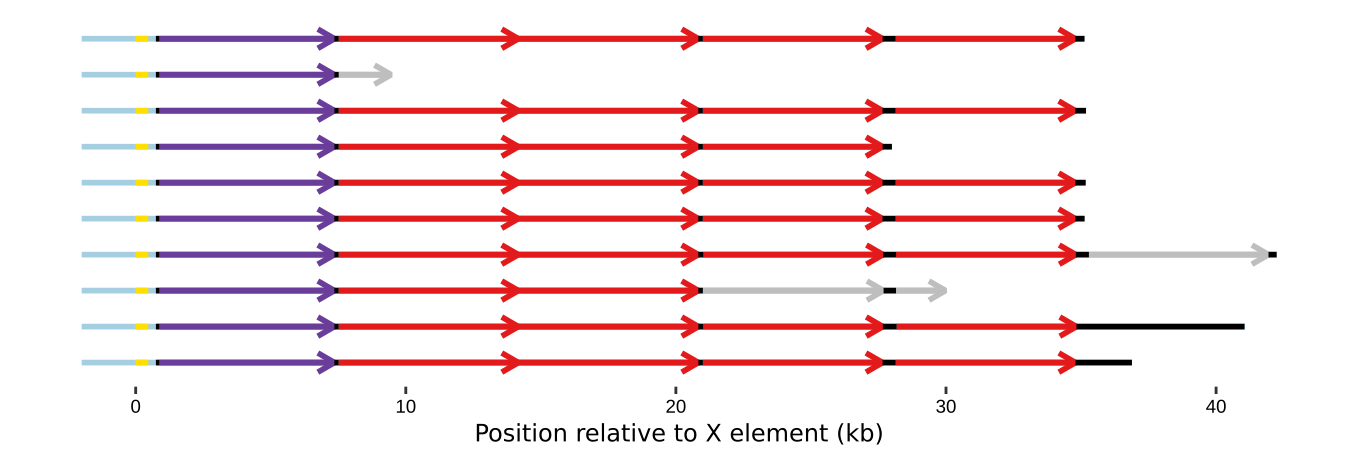

Supplement: Supplementary file 5 — Dataset EV2 [file 44319_2026_717_MOESM5_ESM.zip › Dataset EV2/WT/Chr_XII.right.png]

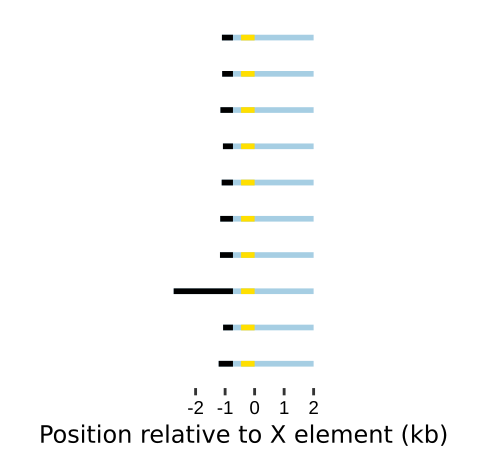

Supplement: Supplementary file 5 — Dataset EV2 [file 44319_2026_717_MOESM5_ESM.zip › Dataset EV2/WT/Chr_III.left.png]

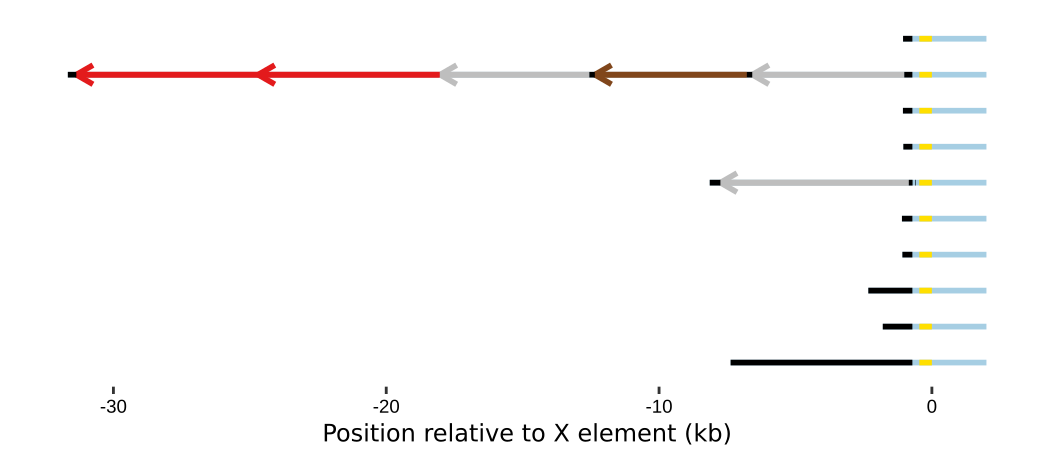

Supplement: Supplementary file 5 — Dataset EV2 [file 44319_2026_717_MOESM5_ESM.zip › Dataset EV2/WT/Chr_IV.left.png]

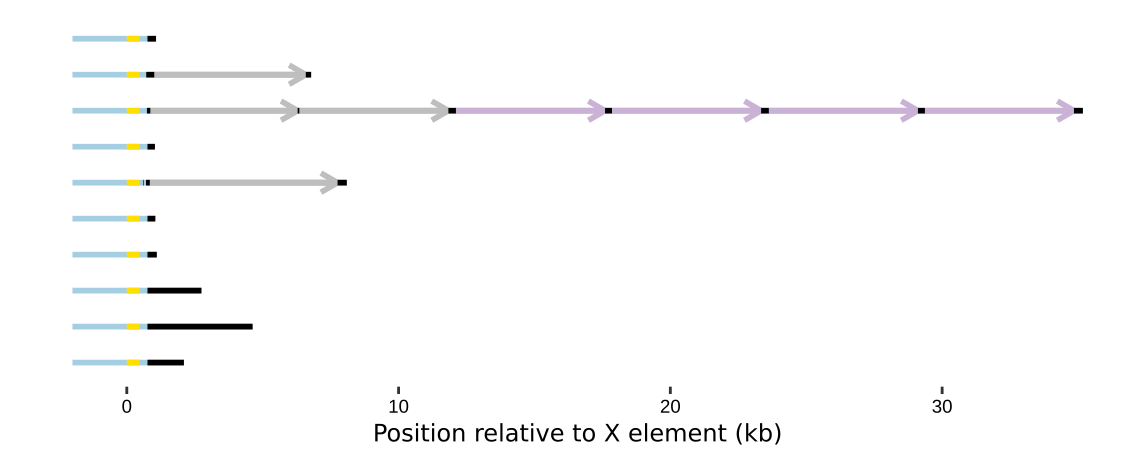

Supplement: Supplementary file 5 — Dataset EV2 [file 44319_2026_717_MOESM5_ESM.zip › Dataset EV2/WT/Chr_X.right.png]

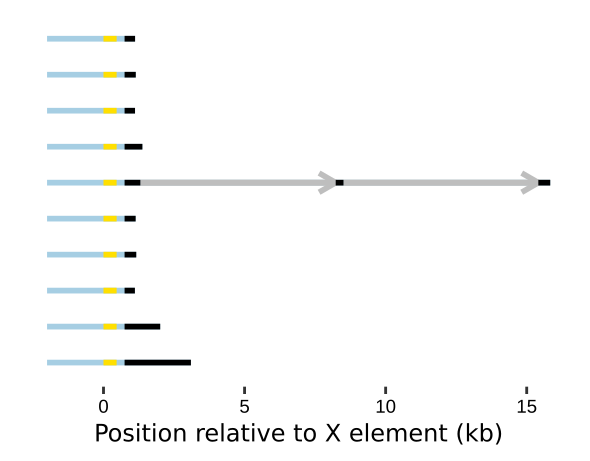

Supplement: Supplementary file 5 — Dataset EV2 [file 44319_2026_717_MOESM5_ESM.zip › Dataset EV2/WT/Chr_IX.right.png]

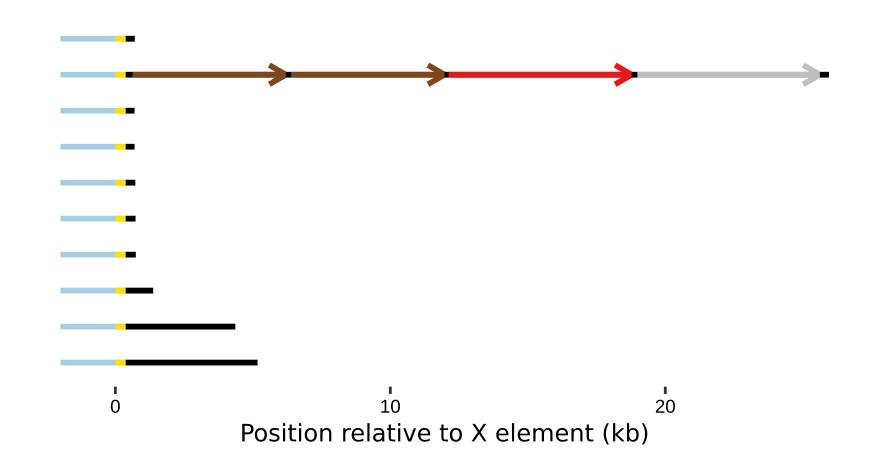

Supplement: Supplementary file 5 — Dataset EV2 [file 44319_2026_717_MOESM5_ESM.zip › Dataset EV2/WT/Chr_VI.right.png]

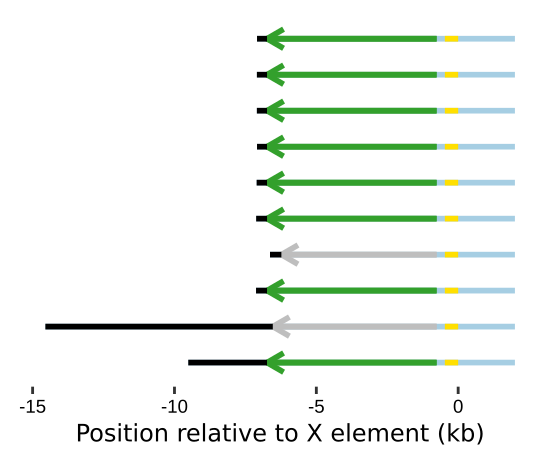

Supplement: Supplementary file 5 — Dataset EV2 [file 44319_2026_717_MOESM5_ESM.zip › Dataset EV2/WT/Chr_II.left.png]

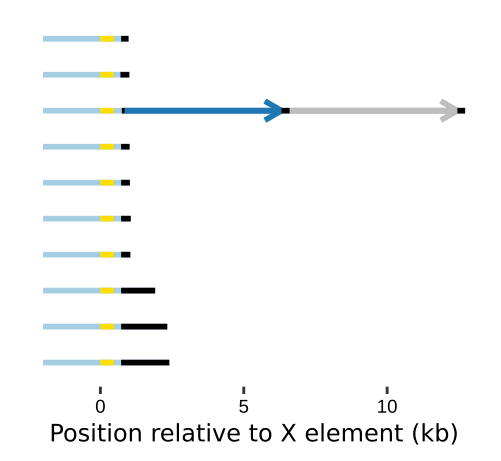

Supplement: Supplementary file 5 — Dataset EV2 [file 44319_2026_717_MOESM5_ESM.zip › Dataset EV2/WT/Chr_VIII.right.png]

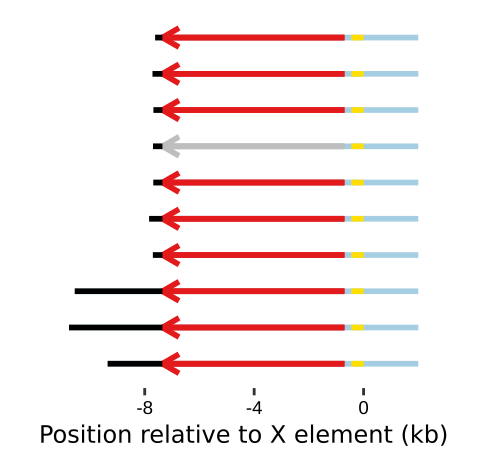

Supplement: Supplementary file 5 — Dataset EV2 [file 44319_2026_717_MOESM5_ESM.zip › Dataset EV2/WT/Chr_XVI.left.png]

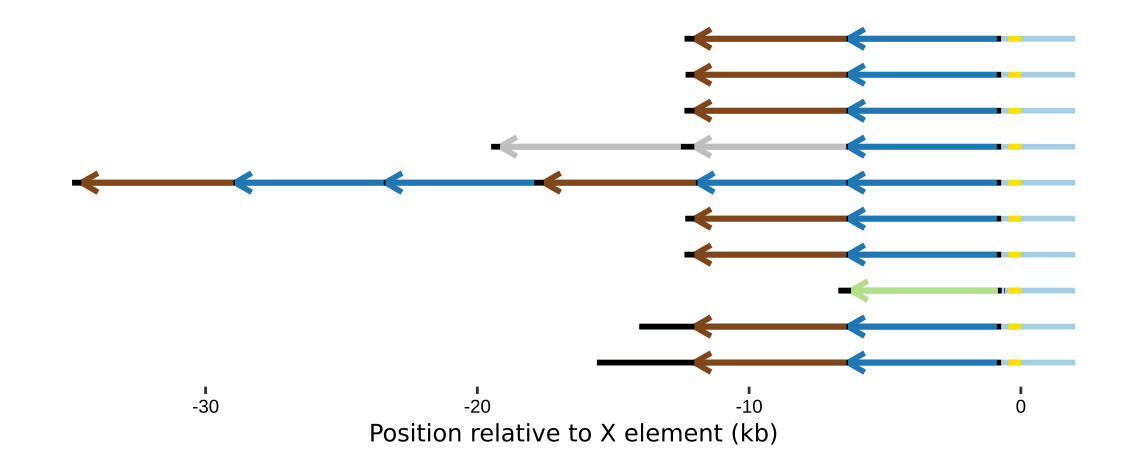

Supplement: Supplementary file 5 — Dataset EV2 [file 44319_2026_717_MOESM5_ESM.zip › Dataset EV2/WT/Chr_XII.left.png]

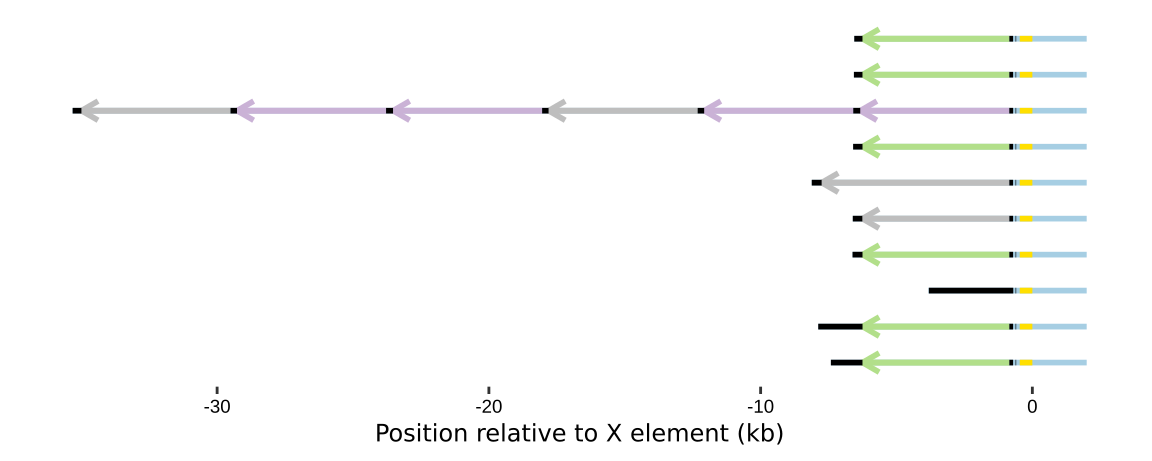

Supplement: Supplementary file 5 — Dataset EV2 [file 44319_2026_717_MOESM5_ESM.zip › Dataset EV2/WT/Chr_VI.left.png]

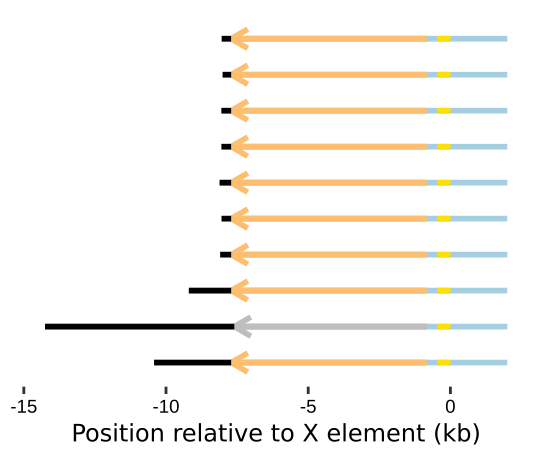

Supplement: Supplementary file 5 — Dataset EV2 [file 44319_2026_717_MOESM5_ESM.zip › Dataset EV2/WT/Chr_X.left.png]

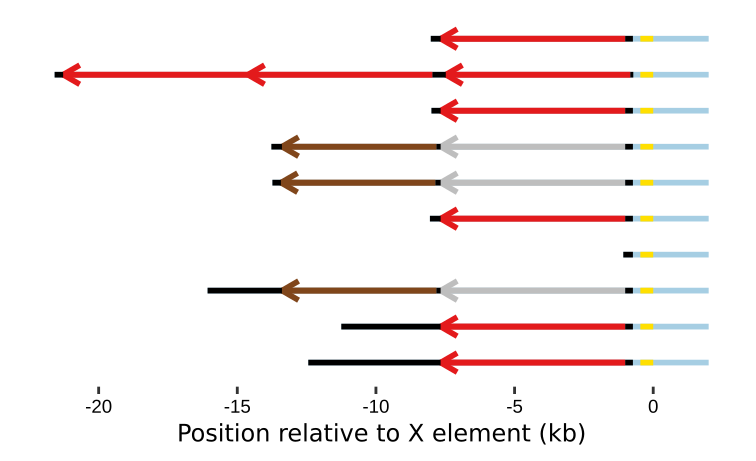

Supplement: Supplementary file 5 — Dataset EV2 [file 44319_2026_717_MOESM5_ESM.zip › Dataset EV2/WT/Chr_XIV.left.png]

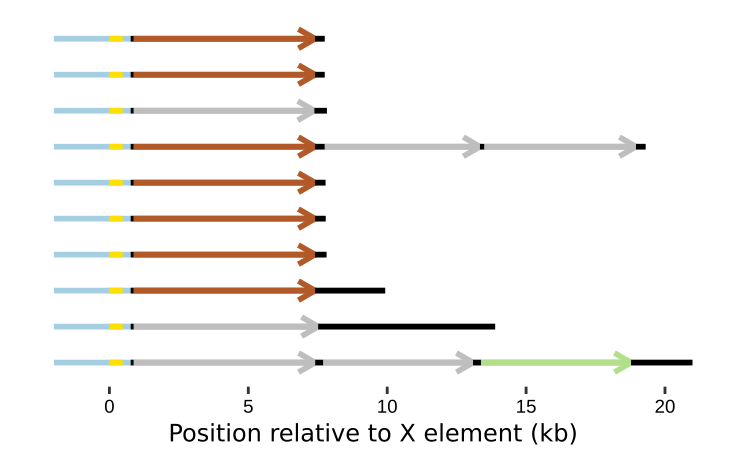

Supplement: Supplementary file 5 — Dataset EV2 [file 44319_2026_717_MOESM5_ESM.zip › Dataset EV2/WT/Chr_XIV.right.png]

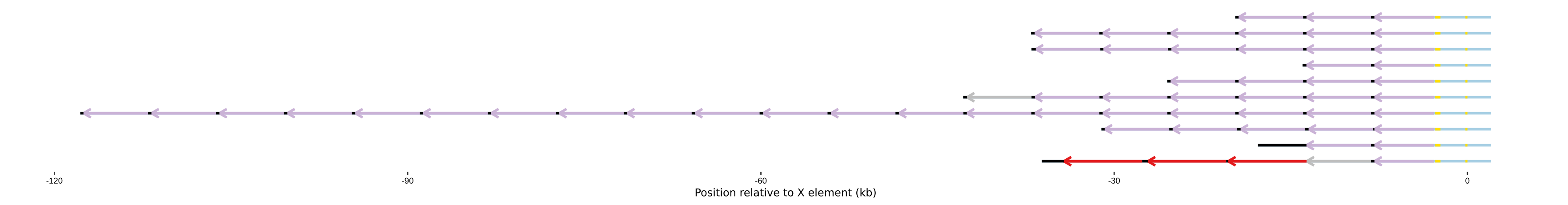

Supplement: Supplementary file 5 — Dataset EV2 [file 44319_2026_717_MOESM5_ESM.zip › Dataset EV2/WT/Chr_VIII.left.png]

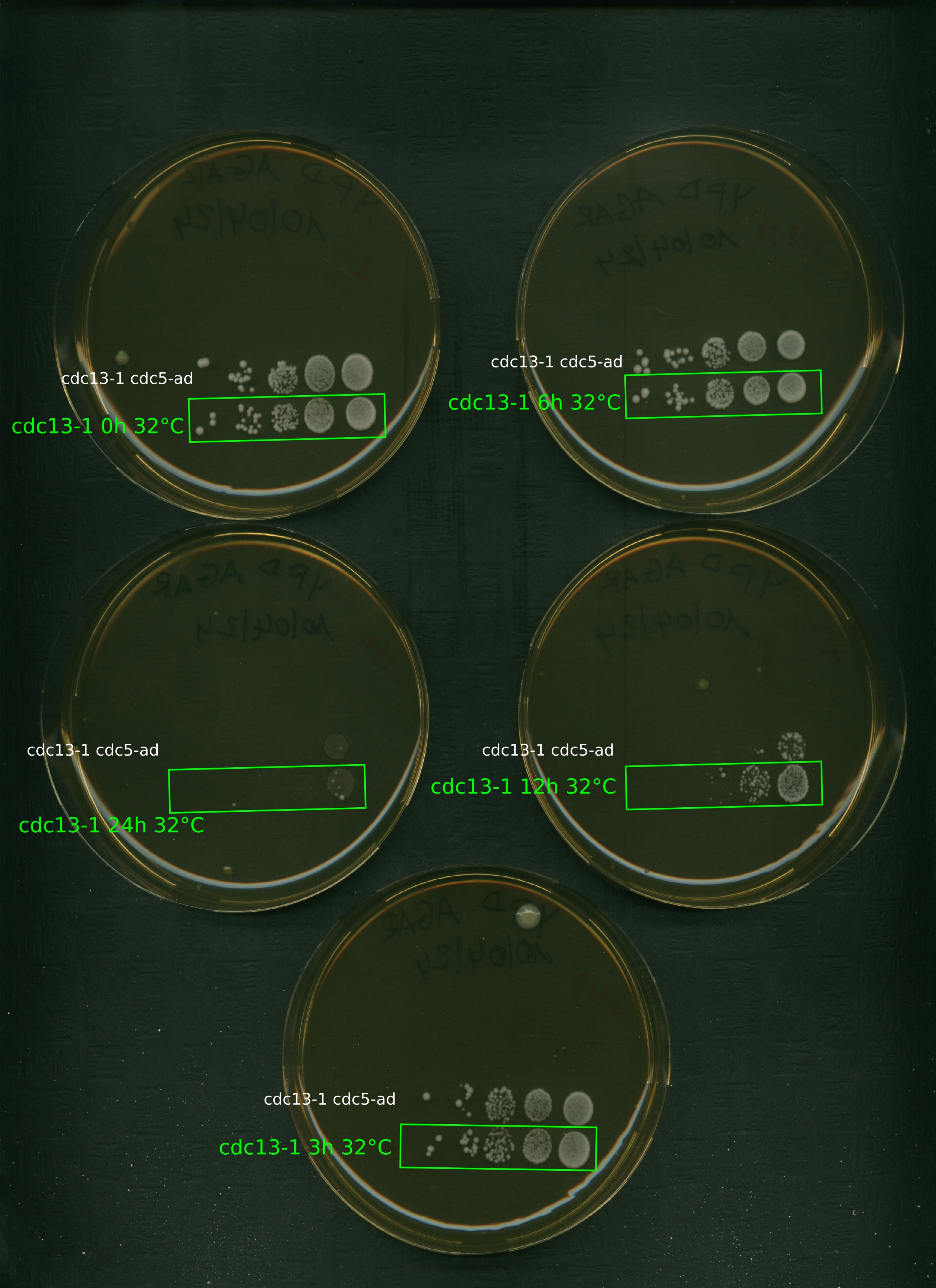

Supplement: Supplementary file 6 — Source data Fig. 1 [file 44319_2026_717_MOESM6_ESM.zip › SourceData_Fig1/1B/rawdata_figure1B.jpg]

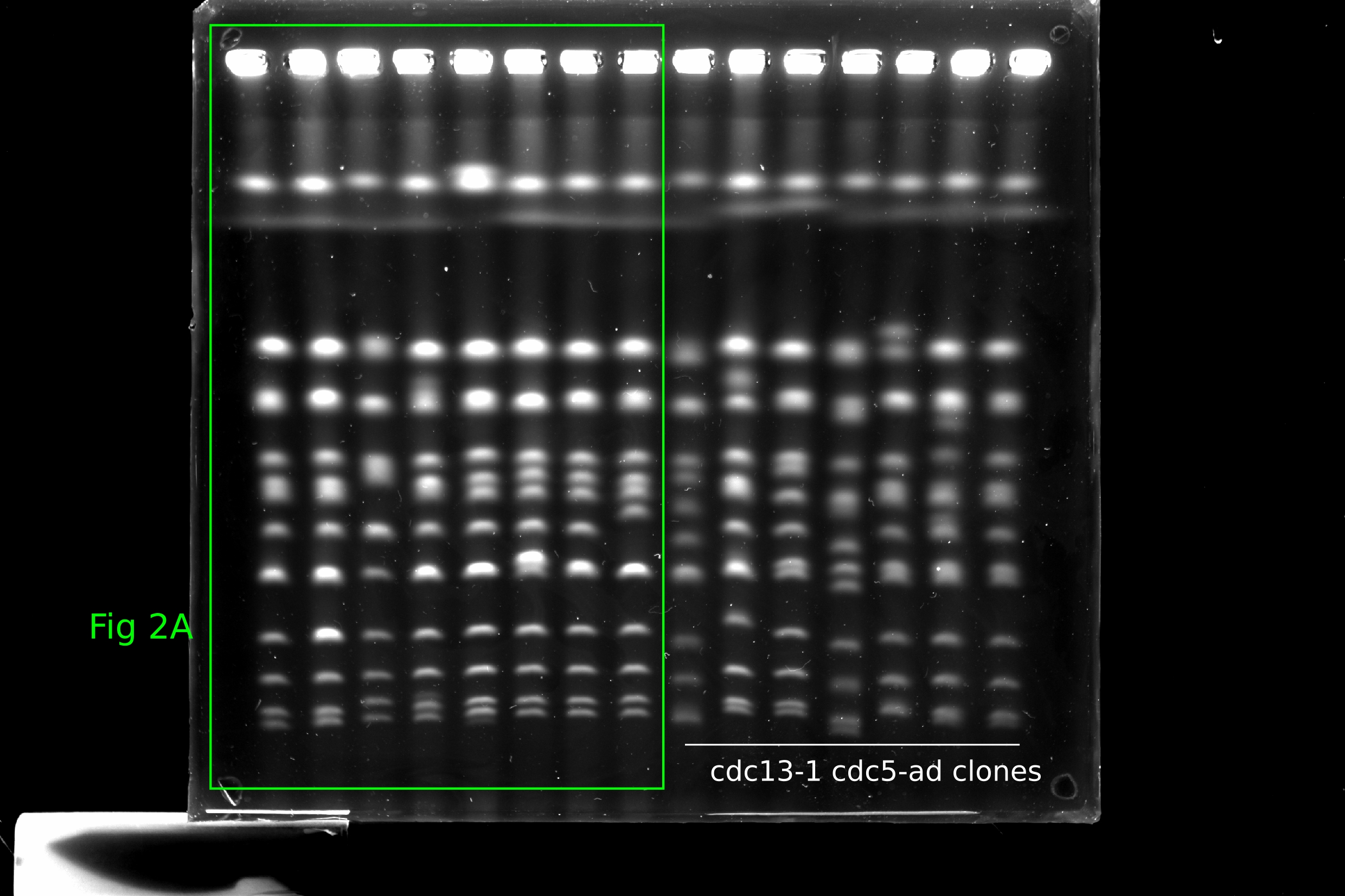

Supplement: Supplementary file 7 — Source data Fig. 2 [file 44319_2026_717_MOESM7_ESM.zip › SourceData_Fig2/2A/rawdata_fig2A.jpg]

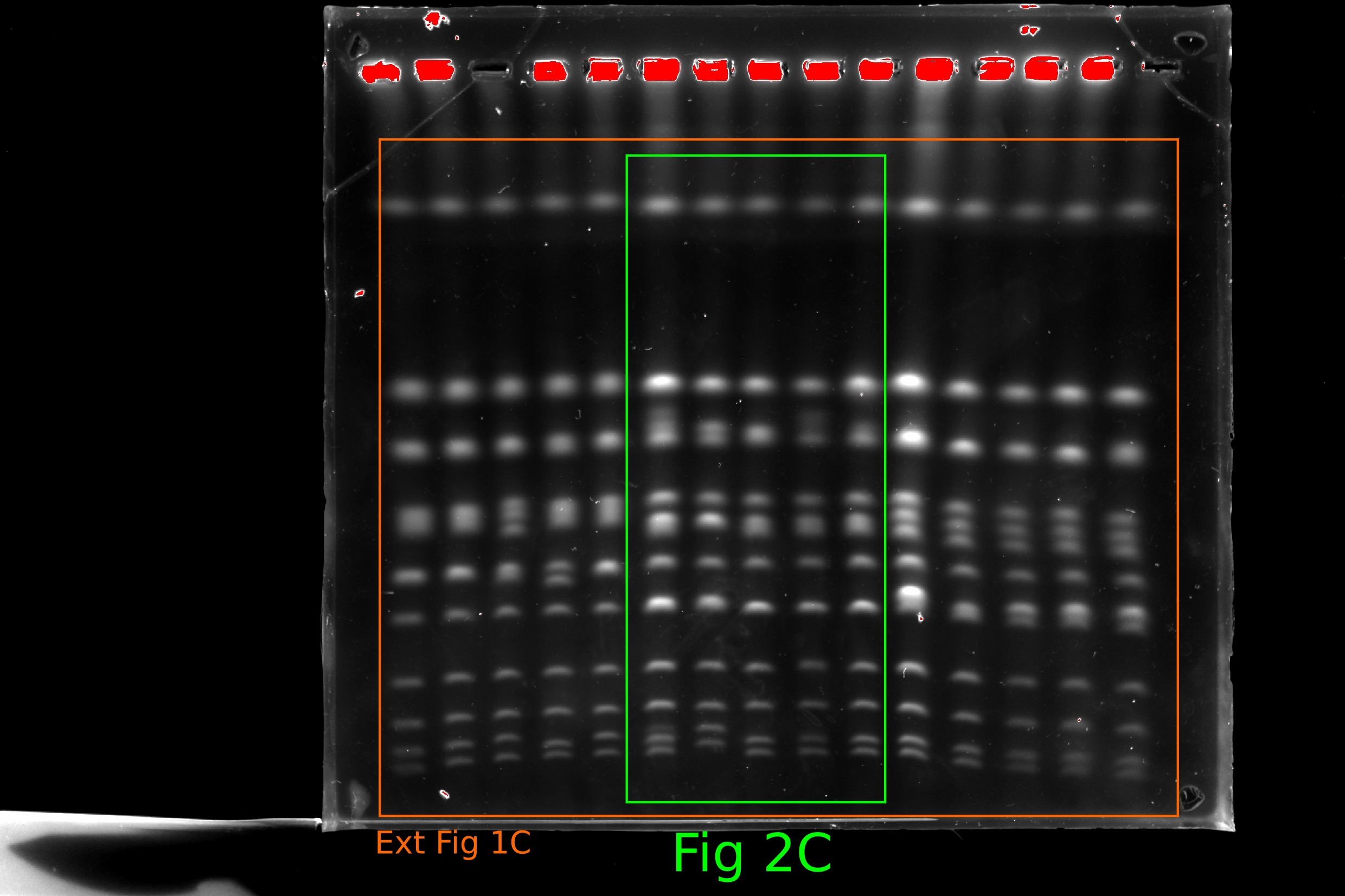

Supplement: Supplementary file 7 — Source data Fig. 2 [file 44319_2026_717_MOESM7_ESM.zip › SourceData_Fig2/2C/rawdata_fig2C.jpg]

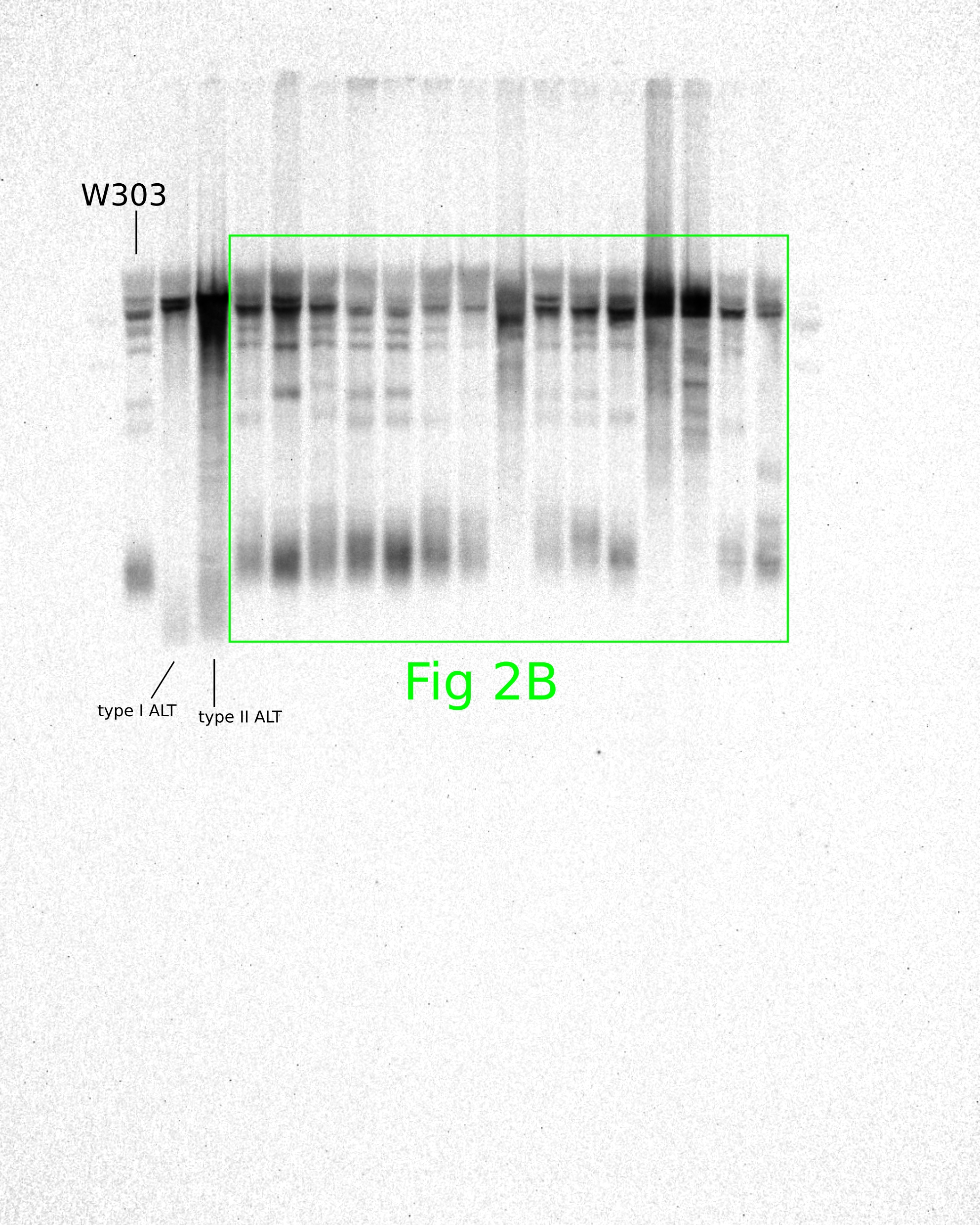

Supplement: Supplementary file 7 — Source data Fig. 2 [file 44319_2026_717_MOESM7_ESM.zip › SourceData_Fig2/2B/rawdata_fig2B.jpg]

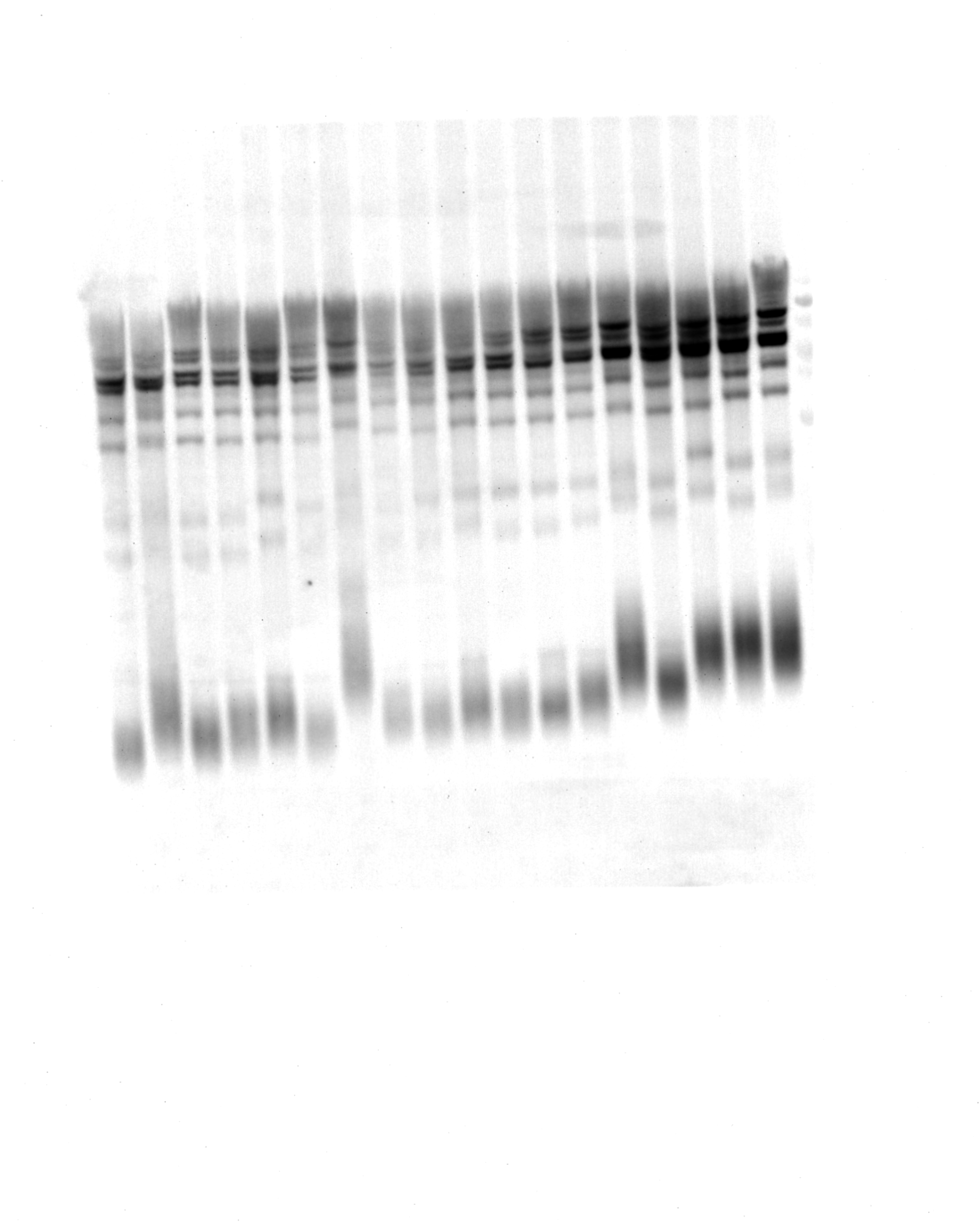

Supplement: Supplementary file 8 — Source data Fig. 5 [file 44319_2026_717_MOESM8_ESM.zip › SourceData_Fig5/5A/rawdata_fig5A.jpg]

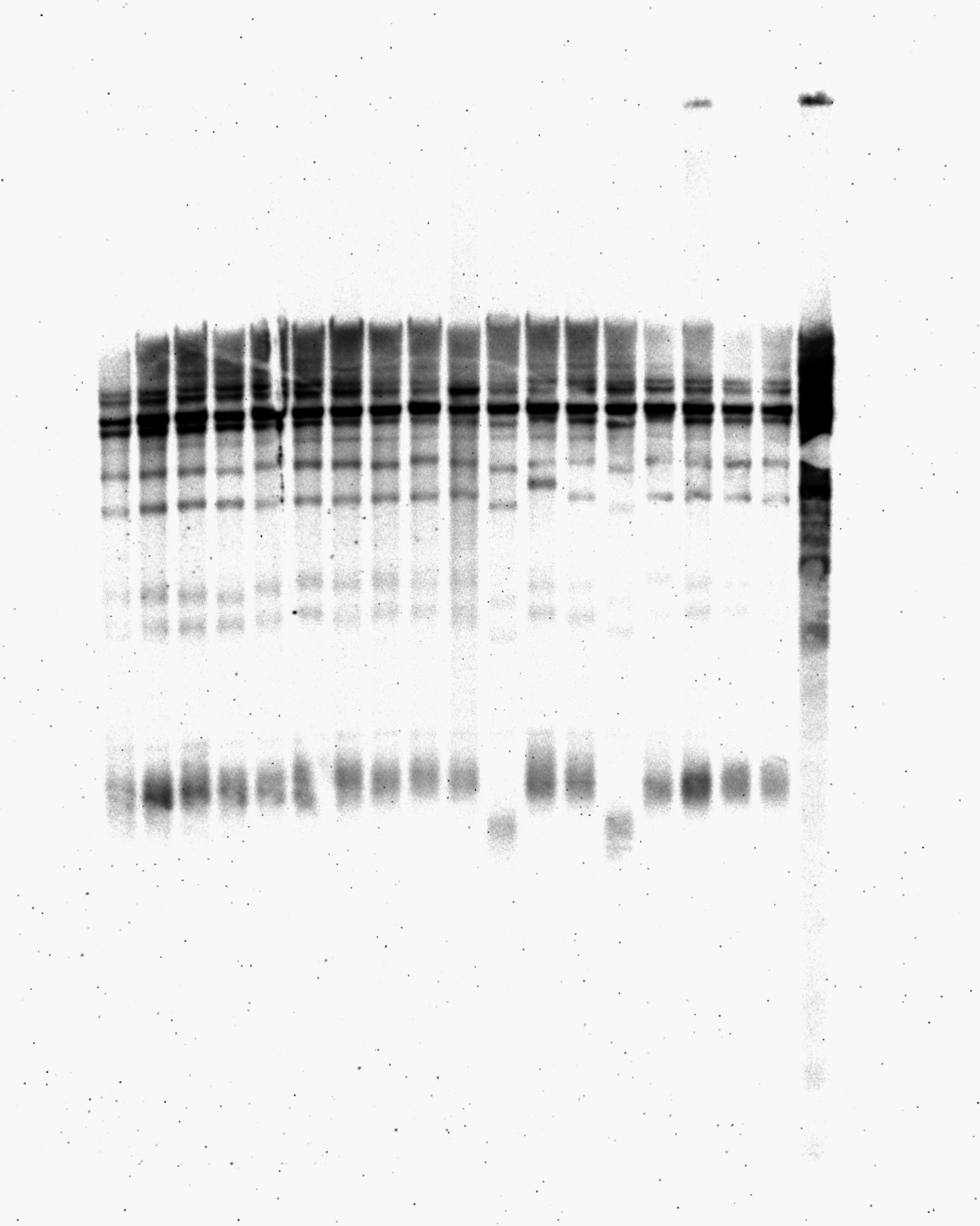

Supplement: Supplementary file 8 — Source data Fig. 5 [file 44319_2026_717_MOESM8_ESM.zip › SourceData_Fig5/5C/rawdata_fig5C.jpg]

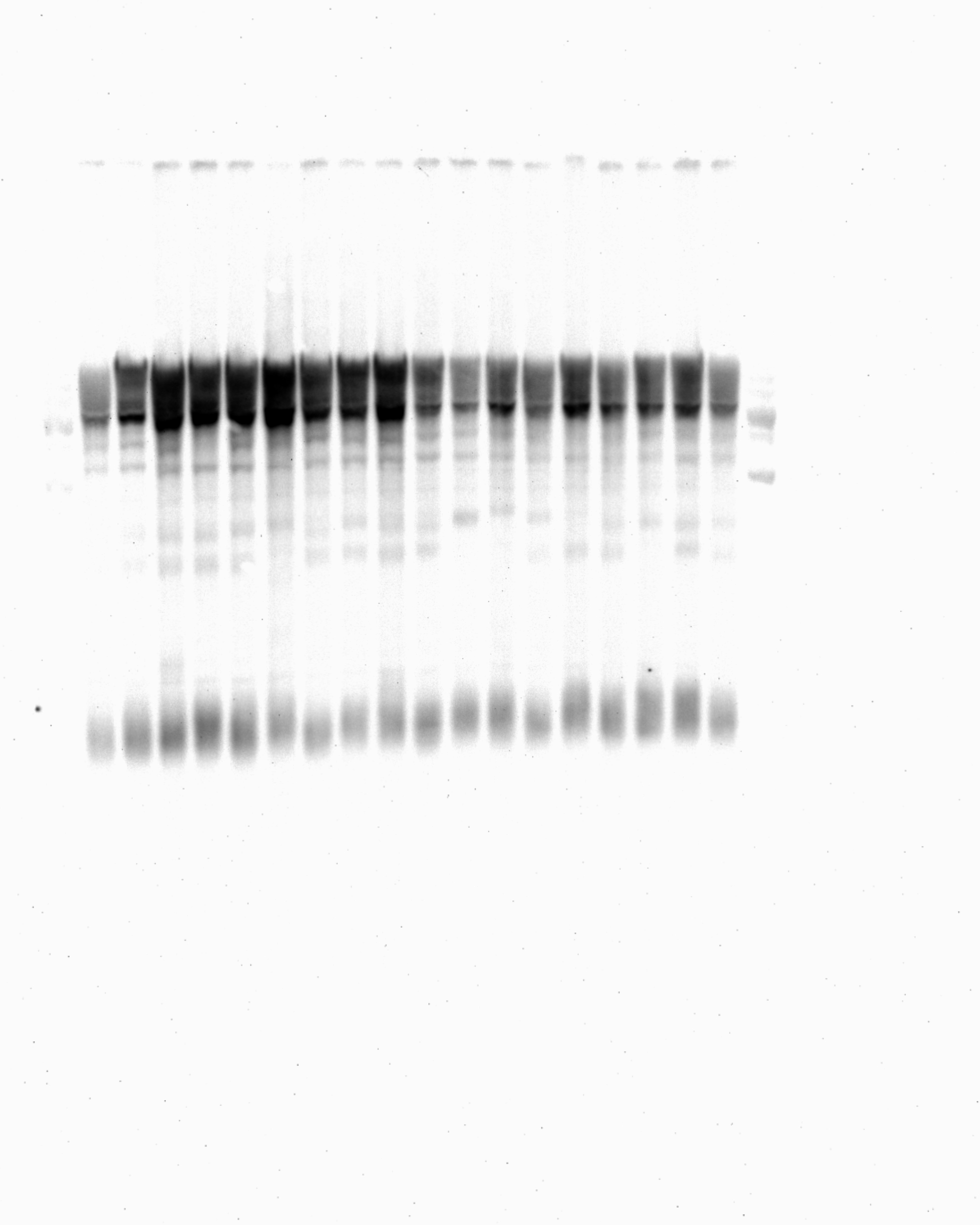

Supplement: Supplementary file 8 — Source data Fig. 5 [file 44319_2026_717_MOESM8_ESM.zip › SourceData_Fig5/5D/rawdata_fig5D.jpg]

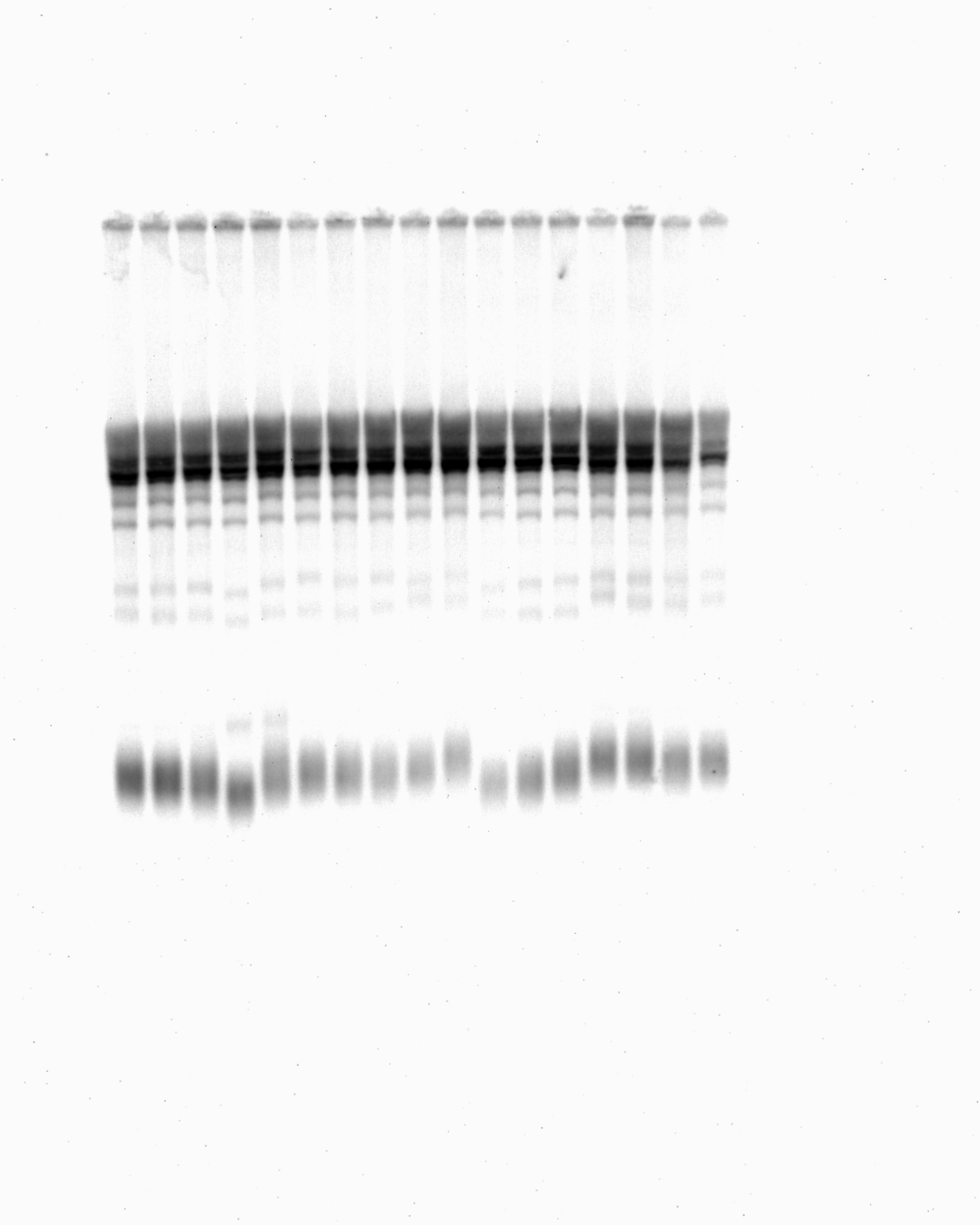

Supplement: Supplementary file 8 — Source data Fig. 5 [file 44319_2026_717_MOESM8_ESM.zip › SourceData_Fig5/5B/rawdata_fig5B.jpg]
