# Supplementary figures and images for: Transient telomere uncapping triggers telomeric and subtelomeric rearrangements (part 1 of 3)
Source: EMBO Rep. 2026 Feb 17;27(6):1607–31. doi: 10.1038/s44319-026-00717-4 (PMC13022453; doi:10.1038/s44319-026-00717-4)

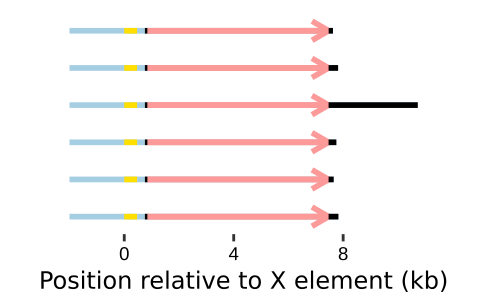

Supplement: Supplementary file 5 — Dataset EV2 [file 44319_2026_717_MOESM5_ESM.zip › Dataset EV2/rad51/Chr_II.right.png]

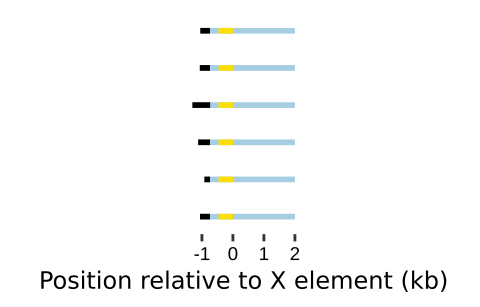

Supplement: Supplementary file 5 — Dataset EV2 [file 44319_2026_717_MOESM5_ESM.zip › Dataset EV2/rad51/Chr_XI.left.png]

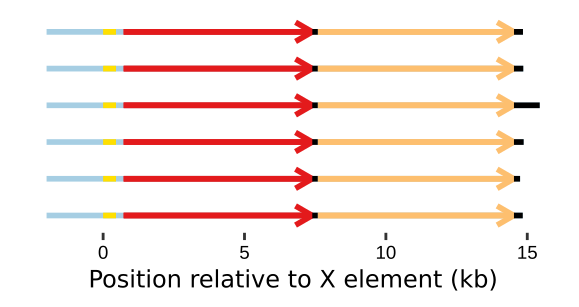

Supplement: Supplementary file 5 — Dataset EV2 [file 44319_2026_717_MOESM5_ESM.zip › Dataset EV2/rad51/Chr_XIII.right.png]

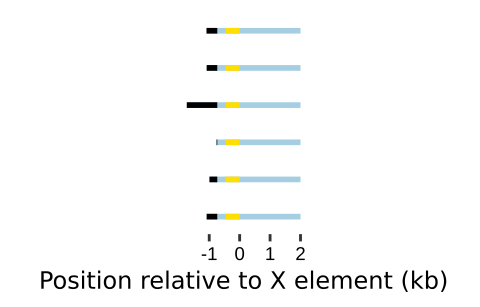

Supplement: Supplementary file 5 — Dataset EV2 [file 44319_2026_717_MOESM5_ESM.zip › Dataset EV2/rad51/Chr_XIII.left.png]

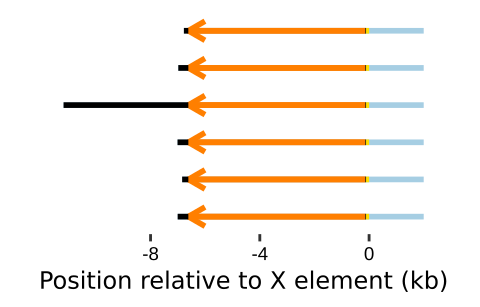

Supplement: Supplementary file 5 — Dataset EV2 [file 44319_2026_717_MOESM5_ESM.zip › Dataset EV2/rad51/Chr_V.left.png]

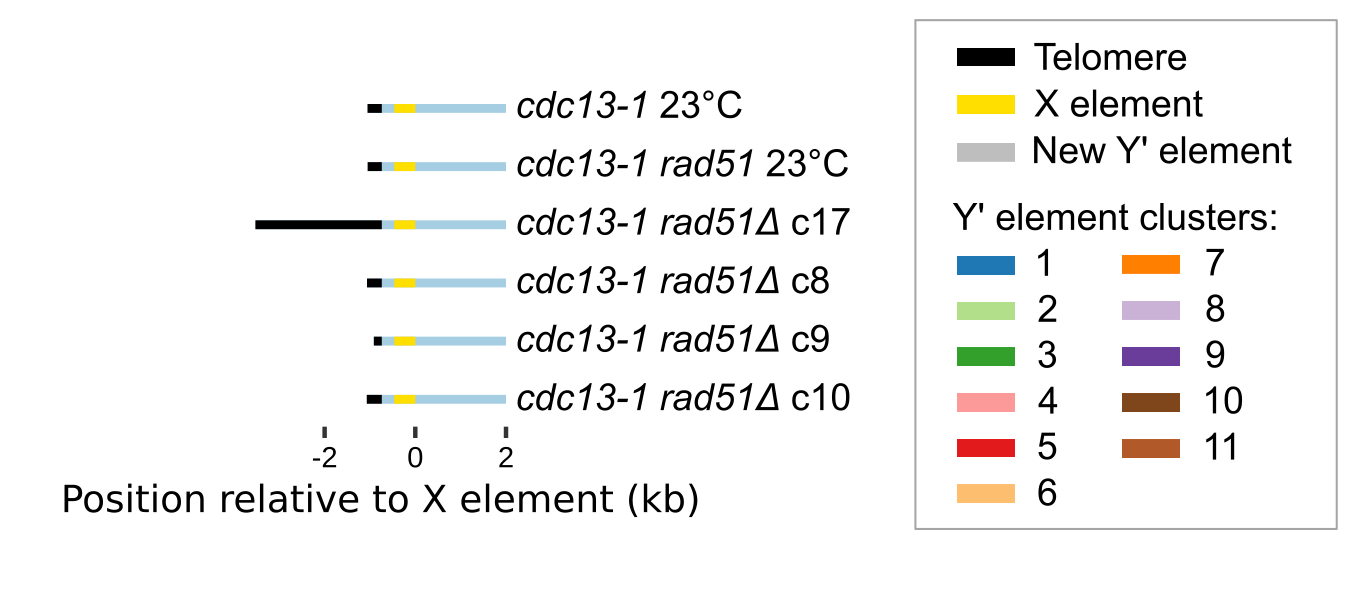

Supplement: Supplementary file 5 — Dataset EV2 [file 44319_2026_717_MOESM5_ESM.zip › Dataset EV2/rad51/Chr_I.left.png]

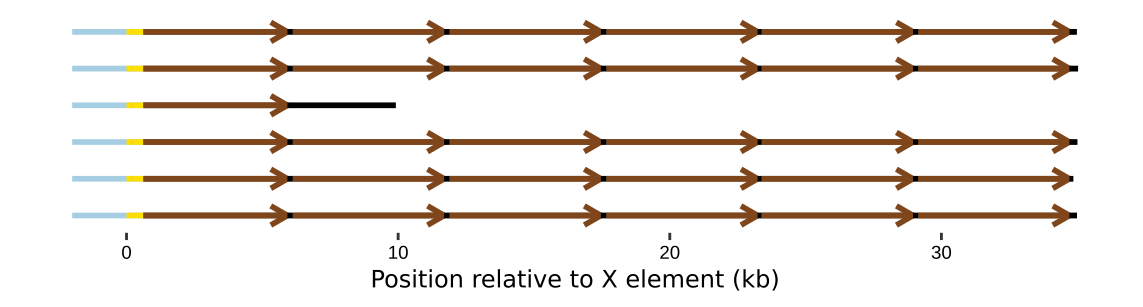

Supplement: Supplementary file 5 — Dataset EV2 [file 44319_2026_717_MOESM5_ESM.zip › Dataset EV2/rad51/Chr_XVI.right.png]

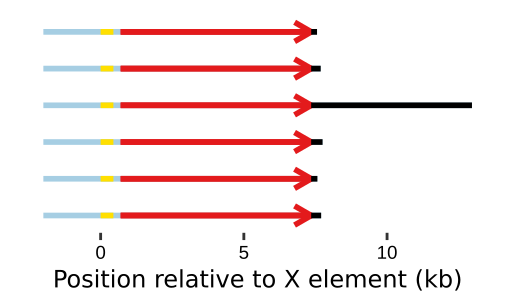

Supplement: Supplementary file 5 — Dataset EV2 [file 44319_2026_717_MOESM5_ESM.zip › Dataset EV2/rad51/Chr_XV.right.png]

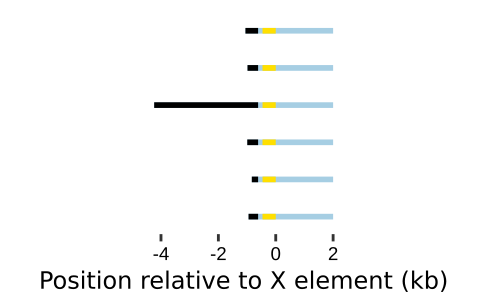

Supplement: Supplementary file 5 — Dataset EV2 [file 44319_2026_717_MOESM5_ESM.zip › Dataset EV2/rad51/Chr_VII.left.png]

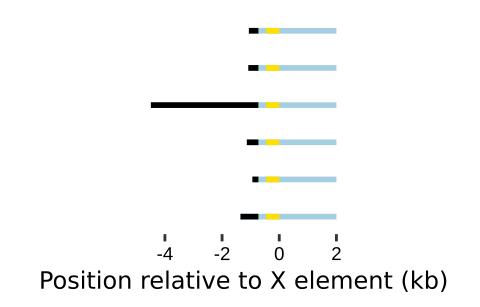

Supplement: Supplementary file 5 — Dataset EV2 [file 44319_2026_717_MOESM5_ESM.zip › Dataset EV2/rad51/Chr_XV.left.png]

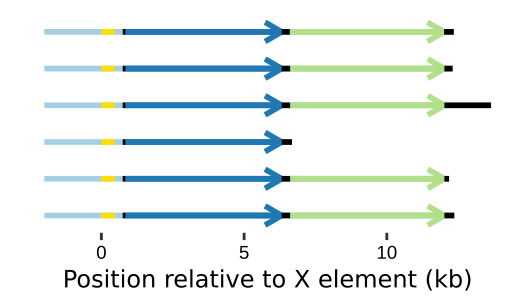

Supplement: Supplementary file 5 — Dataset EV2 [file 44319_2026_717_MOESM5_ESM.zip › Dataset EV2/rad51/Chr_I.right.png]

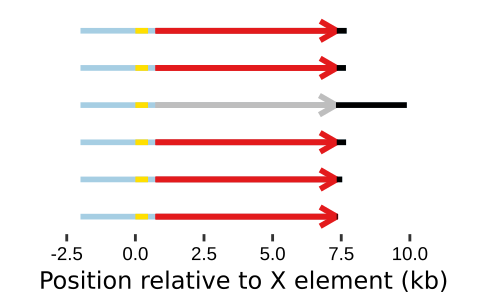

Supplement: Supplementary file 5 — Dataset EV2 [file 44319_2026_717_MOESM5_ESM.zip › Dataset EV2/rad51/Chr_V.right.png]

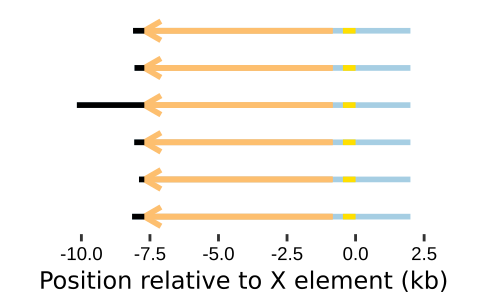

Supplement: Supplementary file 5 — Dataset EV2 [file 44319_2026_717_MOESM5_ESM.zip › Dataset EV2/rad51/Chr_IX.left.png]

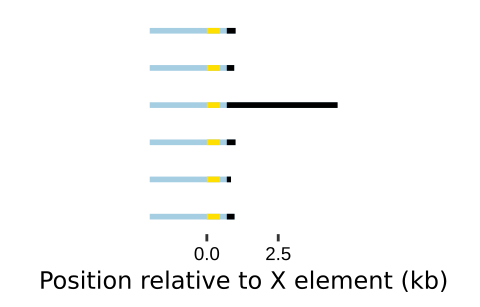

Supplement: Supplementary file 5 — Dataset EV2 [file 44319_2026_717_MOESM5_ESM.zip › Dataset EV2/rad51/Chr_XI.right.png]

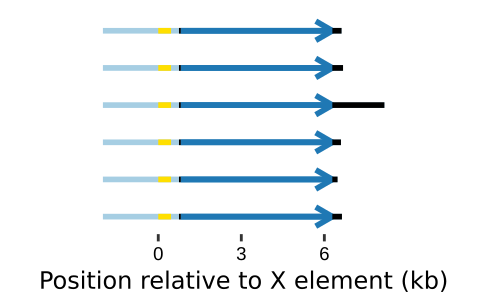

Supplement: Supplementary file 5 — Dataset EV2 [file 44319_2026_717_MOESM5_ESM.zip › Dataset EV2/rad51/Chr_III.right.png]

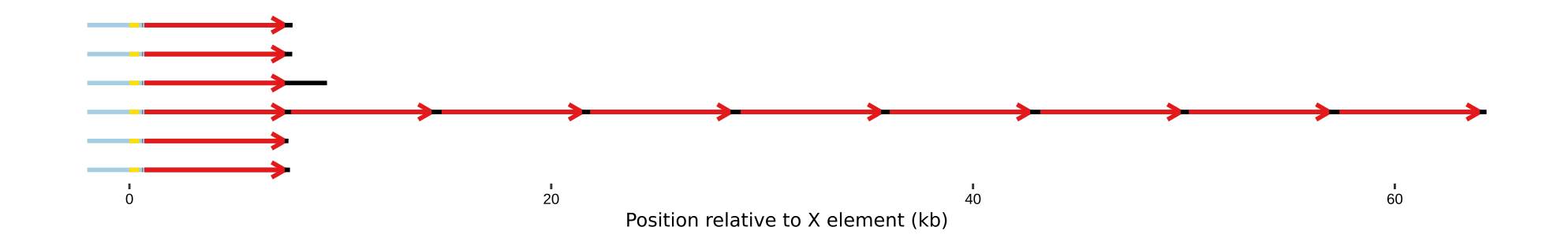

Supplement: Supplementary file 5 — Dataset EV2 [file 44319_2026_717_MOESM5_ESM.zip › Dataset EV2/rad51/Chr_VII.right.png]

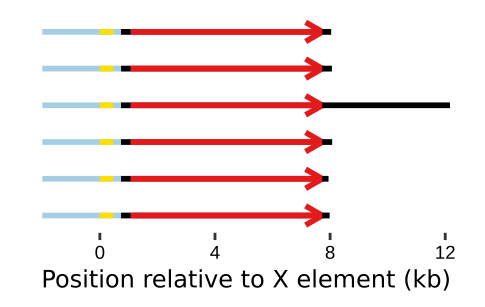

Supplement: Supplementary file 5 — Dataset EV2 [file 44319_2026_717_MOESM5_ESM.zip › Dataset EV2/rad51/Chr_IV.right.png]

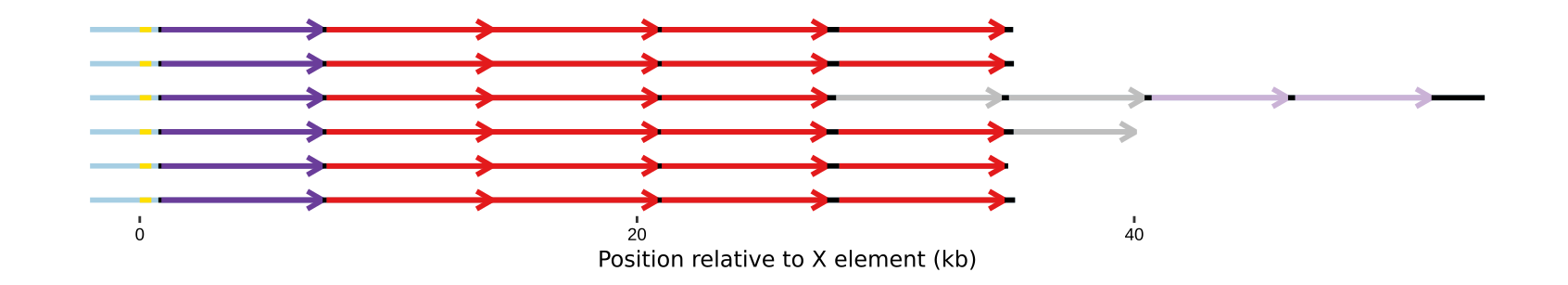

Supplement: Supplementary file 5 — Dataset EV2 [file 44319_2026_717_MOESM5_ESM.zip › Dataset EV2/rad51/Chr_XII.right.png]

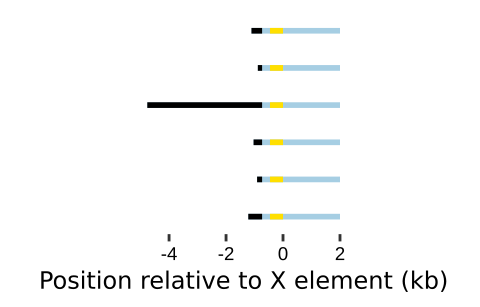

Supplement: Supplementary file 5 — Dataset EV2 [file 44319_2026_717_MOESM5_ESM.zip › Dataset EV2/rad51/Chr_III.left.png]

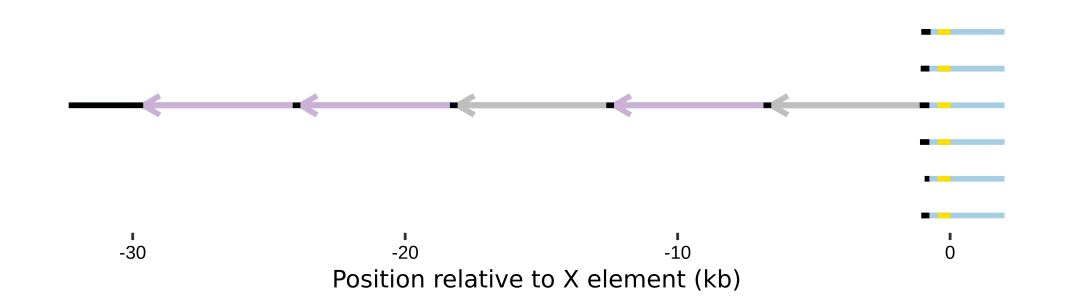

Supplement: Supplementary file 5 — Dataset EV2 [file 44319_2026_717_MOESM5_ESM.zip › Dataset EV2/rad51/Chr_IV.left.png]

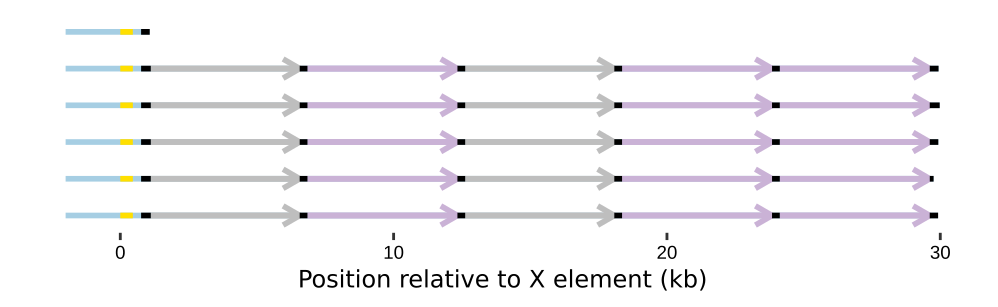

Supplement: Supplementary file 5 — Dataset EV2 [file 44319_2026_717_MOESM5_ESM.zip › Dataset EV2/rad51/Chr_X.right.png]

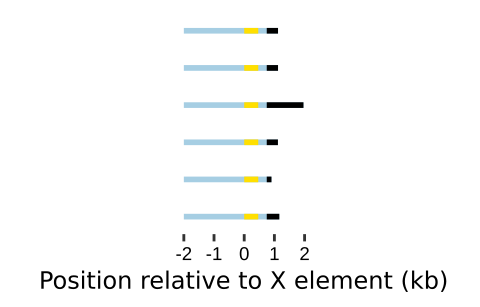

Supplement: Supplementary file 5 — Dataset EV2 [file 44319_2026_717_MOESM5_ESM.zip › Dataset EV2/rad51/Chr_IX.right.png]

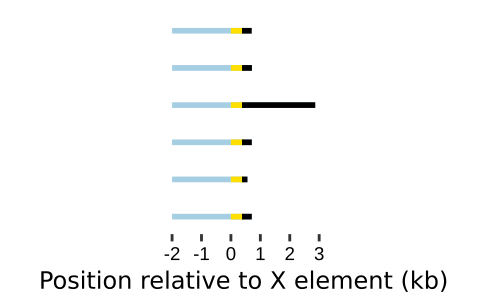

Supplement: Supplementary file 5 — Dataset EV2 [file 44319_2026_717_MOESM5_ESM.zip › Dataset EV2/rad51/Chr_VI.right.png]

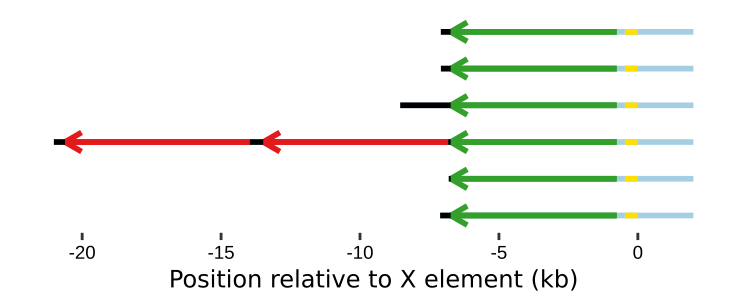

Supplement: Supplementary file 5 — Dataset EV2 [file 44319_2026_717_MOESM5_ESM.zip › Dataset EV2/rad51/Chr_II.left.png]

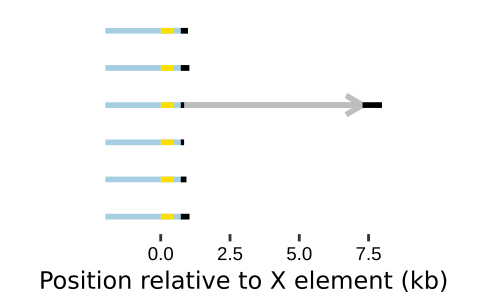

Supplement: Supplementary file 5 — Dataset EV2 [file 44319_2026_717_MOESM5_ESM.zip › Dataset EV2/rad51/Chr_VIII.right.png]

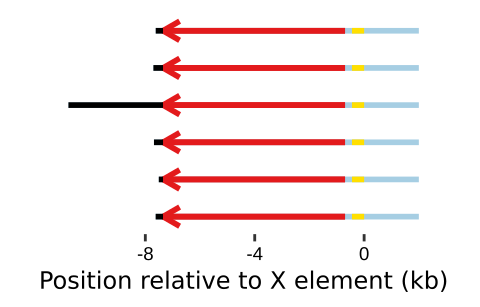

Supplement: Supplementary file 5 — Dataset EV2 [file 44319_2026_717_MOESM5_ESM.zip › Dataset EV2/rad51/Chr_XVI.left.png]

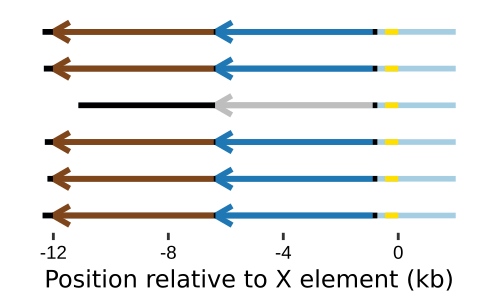

Supplement: Supplementary file 5 — Dataset EV2 [file 44319_2026_717_MOESM5_ESM.zip › Dataset EV2/rad51/Chr_XII.left.png]

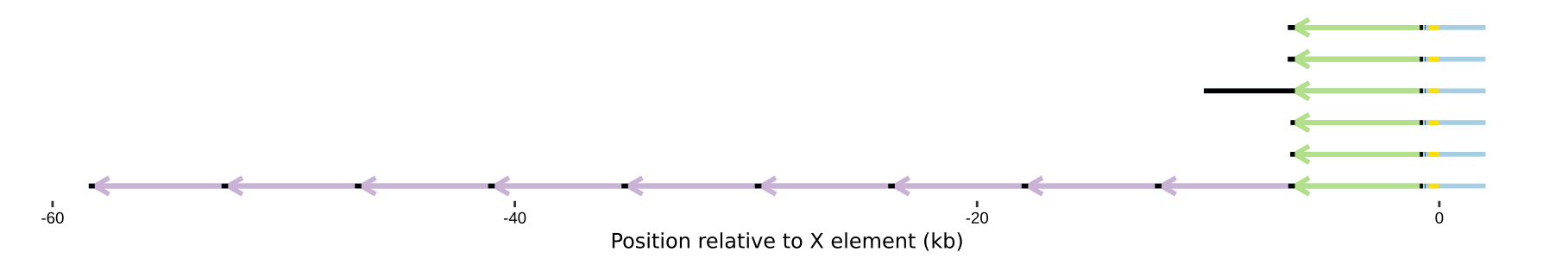

Supplement: Supplementary file 5 — Dataset EV2 [file 44319_2026_717_MOESM5_ESM.zip › Dataset EV2/rad51/Chr_VI.left.png]

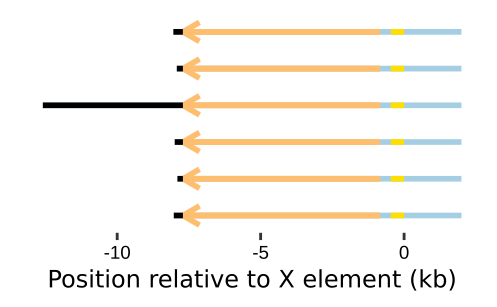

Supplement: Supplementary file 5 — Dataset EV2 [file 44319_2026_717_MOESM5_ESM.zip › Dataset EV2/rad51/Chr_X.left.png]

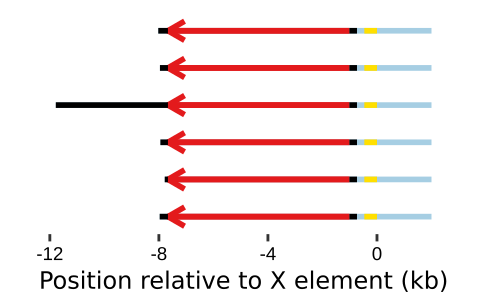

Supplement: Supplementary file 5 — Dataset EV2 [file 44319_2026_717_MOESM5_ESM.zip › Dataset EV2/rad51/Chr_XIV.left.png]

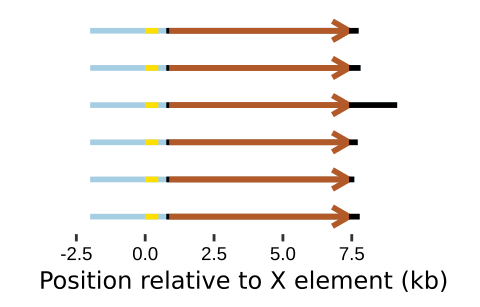

Supplement: Supplementary file 5 — Dataset EV2 [file 44319_2026_717_MOESM5_ESM.zip › Dataset EV2/rad51/Chr_XIV.right.png]

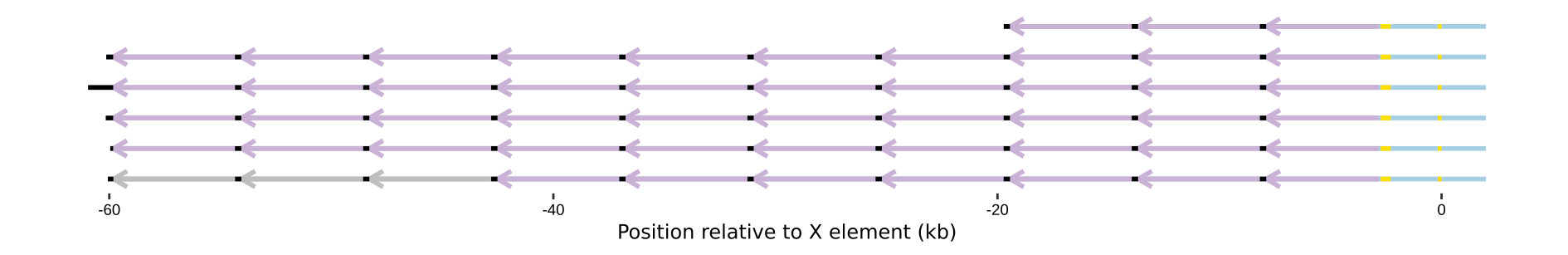

Supplement: Supplementary file 5 — Dataset EV2 [file 44319_2026_717_MOESM5_ESM.zip › Dataset EV2/rad51/Chr_VIII.left.png]

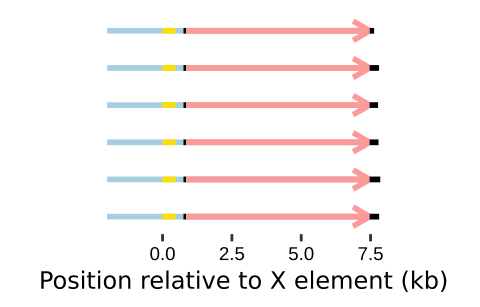

Supplement: Supplementary file 5 — Dataset EV2 [file 44319_2026_717_MOESM5_ESM.zip › Dataset EV2/rad59/Chr_II.right.png]

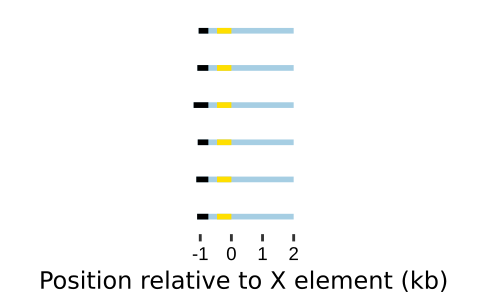

Supplement: Supplementary file 5 — Dataset EV2 [file 44319_2026_717_MOESM5_ESM.zip › Dataset EV2/rad59/Chr_XI.left.png]

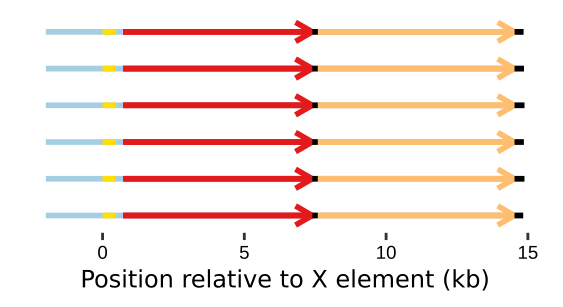

Supplement: Supplementary file 5 — Dataset EV2 [file 44319_2026_717_MOESM5_ESM.zip › Dataset EV2/rad59/Chr_XIII.right.png]

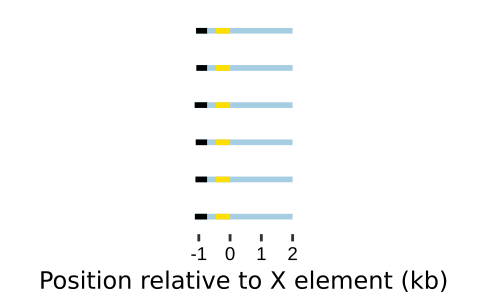

Supplement: Supplementary file 5 — Dataset EV2 [file 44319_2026_717_MOESM5_ESM.zip › Dataset EV2/rad59/Chr_XIII.left.png]

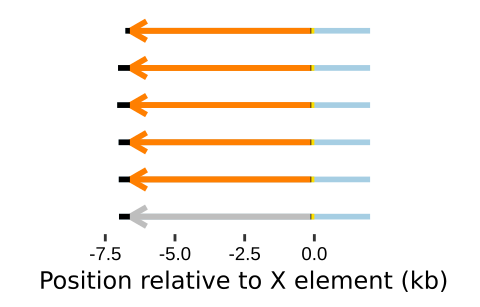

Supplement: Supplementary file 5 — Dataset EV2 [file 44319_2026_717_MOESM5_ESM.zip › Dataset EV2/rad59/Chr_V.left.png]

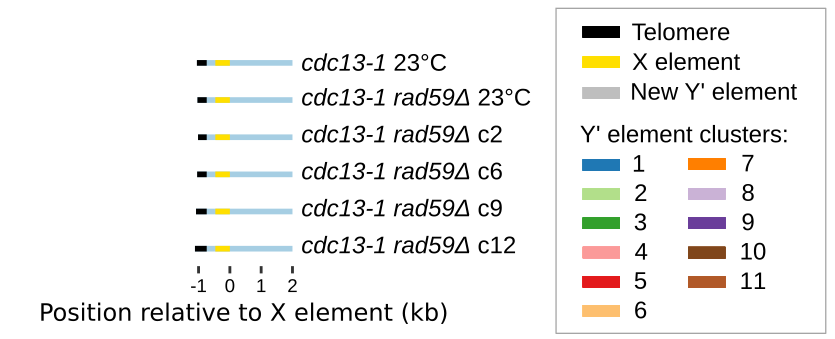

Supplement: Supplementary file 5 — Dataset EV2 [file 44319_2026_717_MOESM5_ESM.zip › Dataset EV2/rad59/Chr_I.left.png]

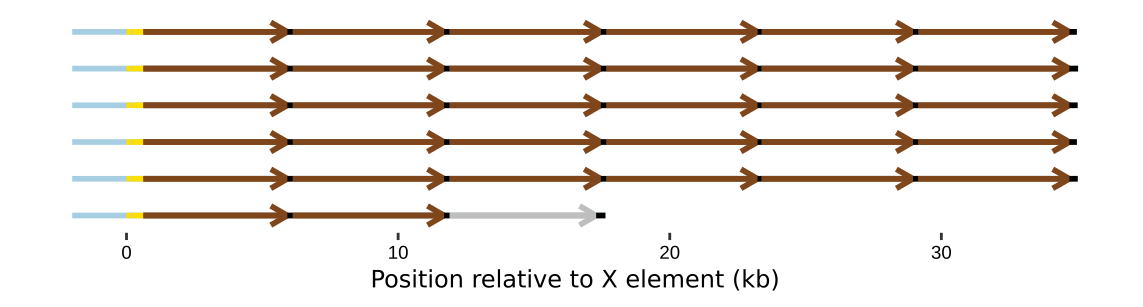

Supplement: Supplementary file 5 — Dataset EV2 [file 44319_2026_717_MOESM5_ESM.zip › Dataset EV2/rad59/Chr_XVI.right.png]

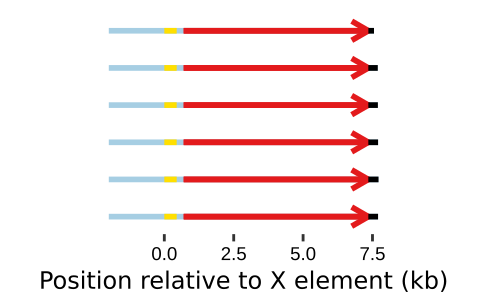

Supplement: Supplementary file 5 — Dataset EV2 [file 44319_2026_717_MOESM5_ESM.zip › Dataset EV2/rad59/Chr_XV.right.png]

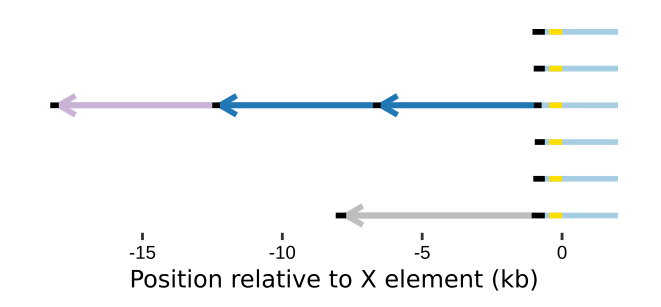

Supplement: Supplementary file 5 — Dataset EV2 [file 44319_2026_717_MOESM5_ESM.zip › Dataset EV2/rad59/Chr_VII.left.png]

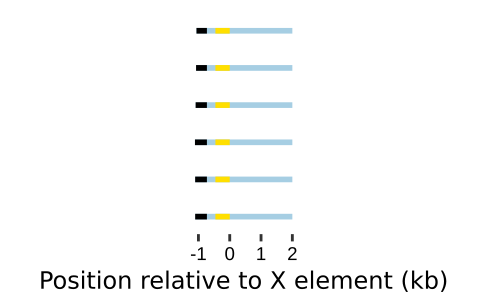

Supplement: Supplementary file 5 — Dataset EV2 [file 44319_2026_717_MOESM5_ESM.zip › Dataset EV2/rad59/Chr_XV.left.png]

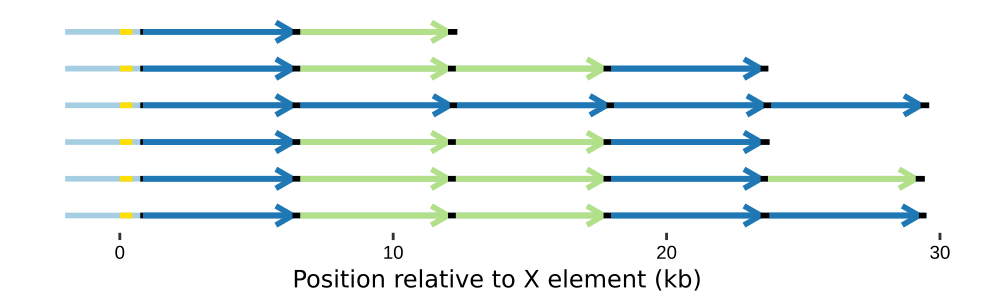

Supplement: Supplementary file 5 — Dataset EV2 [file 44319_2026_717_MOESM5_ESM.zip › Dataset EV2/rad59/Chr_I.right.png]

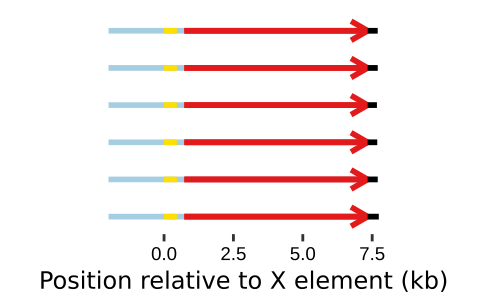

Supplement: Supplementary file 5 — Dataset EV2 [file 44319_2026_717_MOESM5_ESM.zip › Dataset EV2/rad59/Chr_V.right.png]

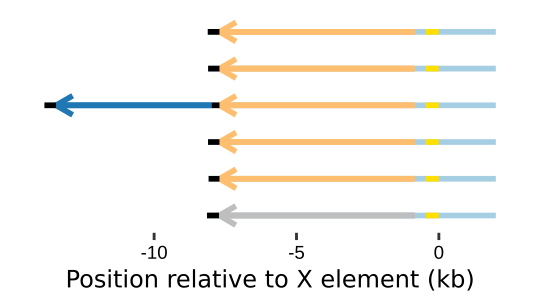

Supplement: Supplementary file 5 — Dataset EV2 [file 44319_2026_717_MOESM5_ESM.zip › Dataset EV2/rad59/Chr_IX.left.png]

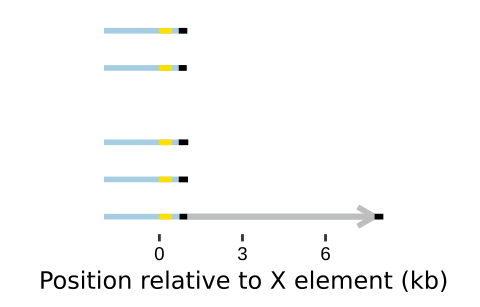

Supplement: Supplementary file 5 — Dataset EV2 [file 44319_2026_717_MOESM5_ESM.zip › Dataset EV2/rad59/Chr_XI.right.png]

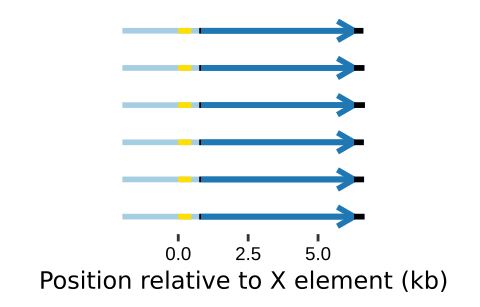

Supplement: Supplementary file 5 — Dataset EV2 [file 44319_2026_717_MOESM5_ESM.zip › Dataset EV2/rad59/Chr_III.right.png]

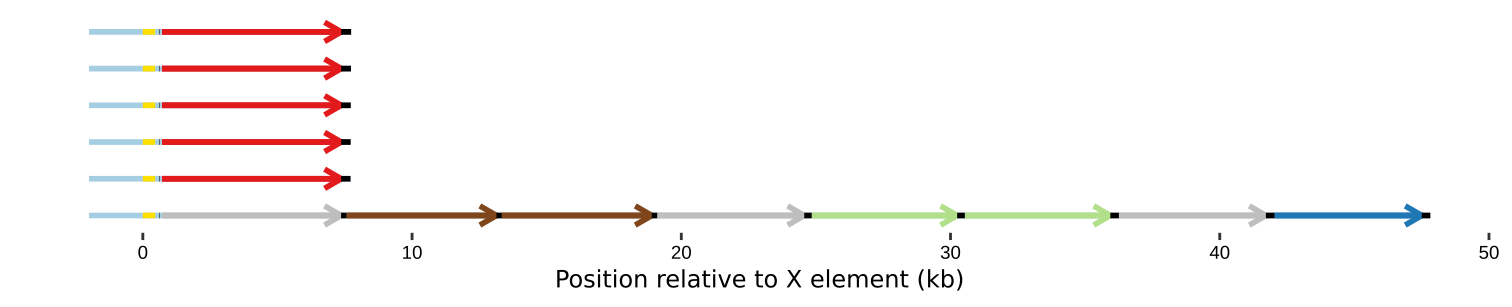

Supplement: Supplementary file 5 — Dataset EV2 [file 44319_2026_717_MOESM5_ESM.zip › Dataset EV2/rad59/Chr_VII.right.png]

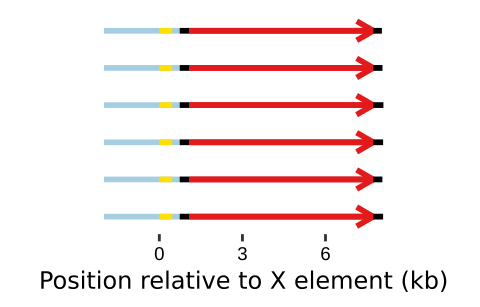

Supplement: Supplementary file 5 — Dataset EV2 [file 44319_2026_717_MOESM5_ESM.zip › Dataset EV2/rad59/Chr_IV.right.png]

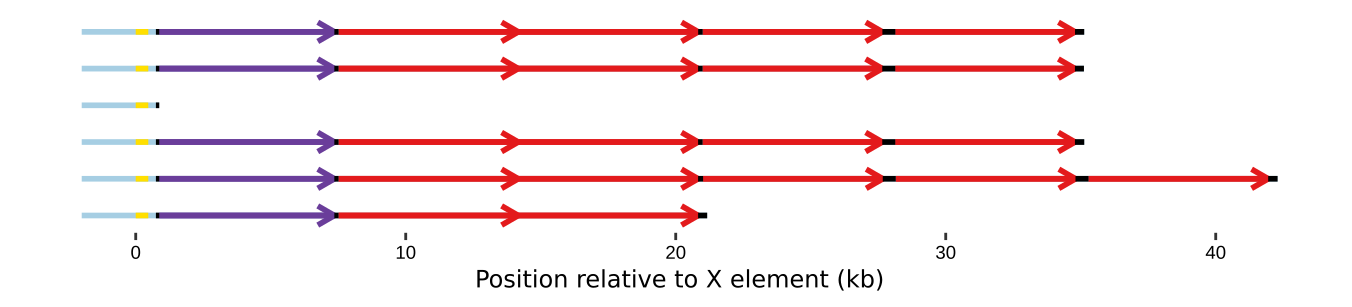

Supplement: Supplementary file 5 — Dataset EV2 [file 44319_2026_717_MOESM5_ESM.zip › Dataset EV2/rad59/Chr_XII.right.png]

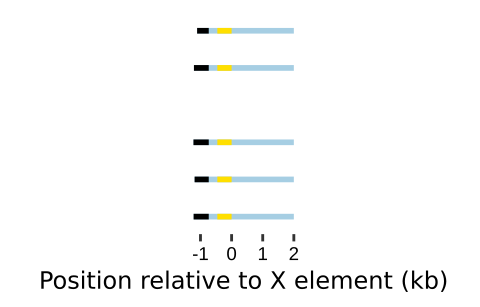

Supplement: Supplementary file 5 — Dataset EV2 [file 44319_2026_717_MOESM5_ESM.zip › Dataset EV2/rad59/Chr_III.left.png]

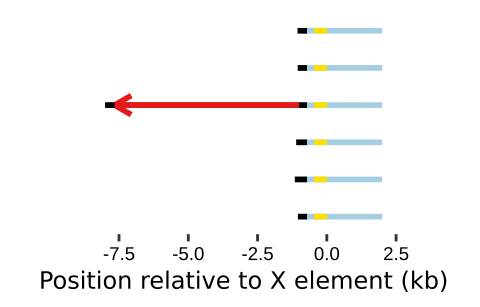

Supplement: Supplementary file 5 — Dataset EV2 [file 44319_2026_717_MOESM5_ESM.zip › Dataset EV2/rad59/Chr_IV.left.png]

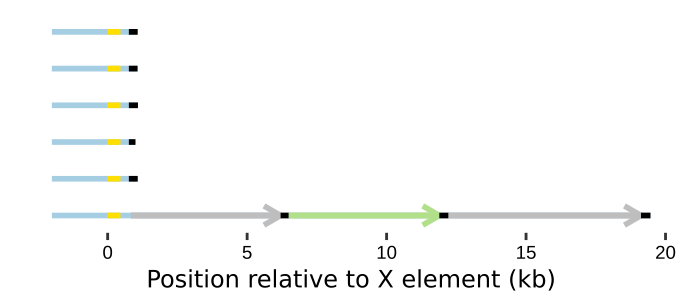

Supplement: Supplementary file 5 — Dataset EV2 [file 44319_2026_717_MOESM5_ESM.zip › Dataset EV2/rad59/Chr_X.right.png]

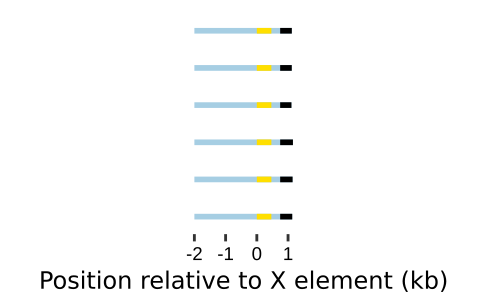

Supplement: Supplementary file 5 — Dataset EV2 [file 44319_2026_717_MOESM5_ESM.zip › Dataset EV2/rad59/Chr_IX.right.png]

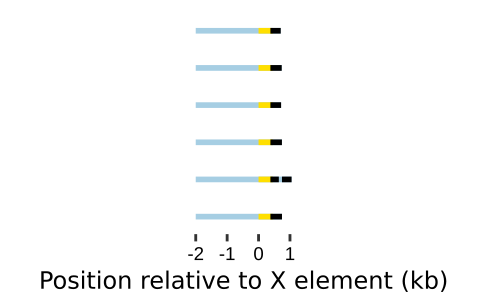

Supplement: Supplementary file 5 — Dataset EV2 [file 44319_2026_717_MOESM5_ESM.zip › Dataset EV2/rad59/Chr_VI.right.png]

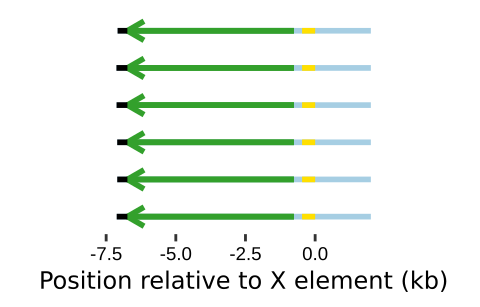

Supplement: Supplementary file 5 — Dataset EV2 [file 44319_2026_717_MOESM5_ESM.zip › Dataset EV2/rad59/Chr_II.left.png]

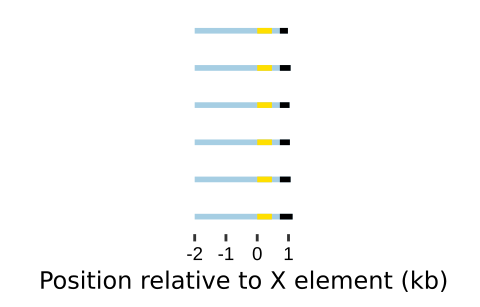

Supplement: Supplementary file 5 — Dataset EV2 [file 44319_2026_717_MOESM5_ESM.zip › Dataset EV2/rad59/Chr_VIII.right.png]

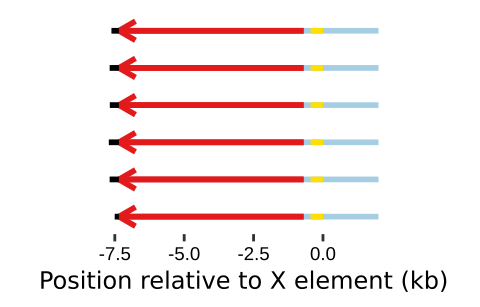

Supplement: Supplementary file 5 — Dataset EV2 [file 44319_2026_717_MOESM5_ESM.zip › Dataset EV2/rad59/Chr_XVI.left.png]

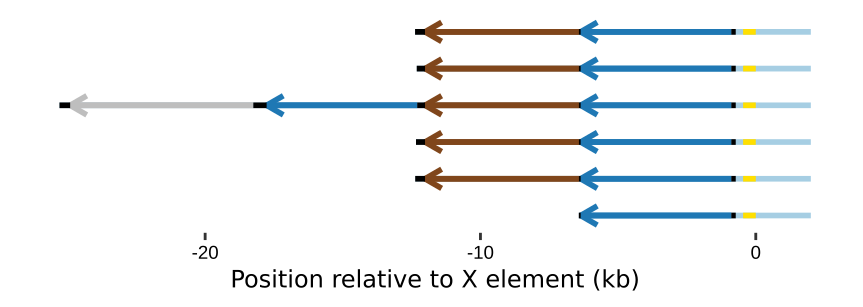

Supplement: Supplementary file 5 — Dataset EV2 [file 44319_2026_717_MOESM5_ESM.zip › Dataset EV2/rad59/Chr_XII.left.png]

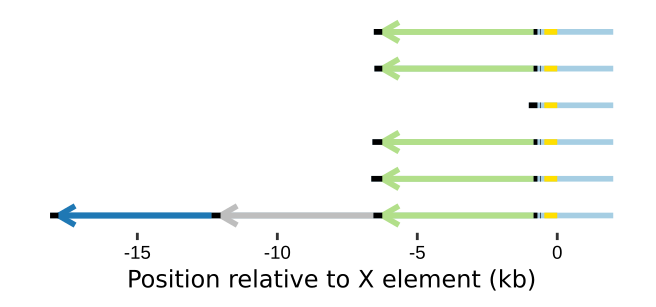

Supplement: Supplementary file 5 — Dataset EV2 [file 44319_2026_717_MOESM5_ESM.zip › Dataset EV2/rad59/Chr_VI.left.png]

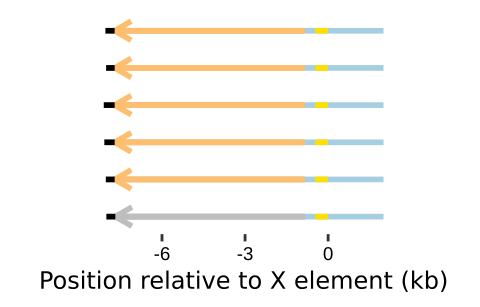

Supplement: Supplementary file 5 — Dataset EV2 [file 44319_2026_717_MOESM5_ESM.zip › Dataset EV2/rad59/Chr_X.left.png]

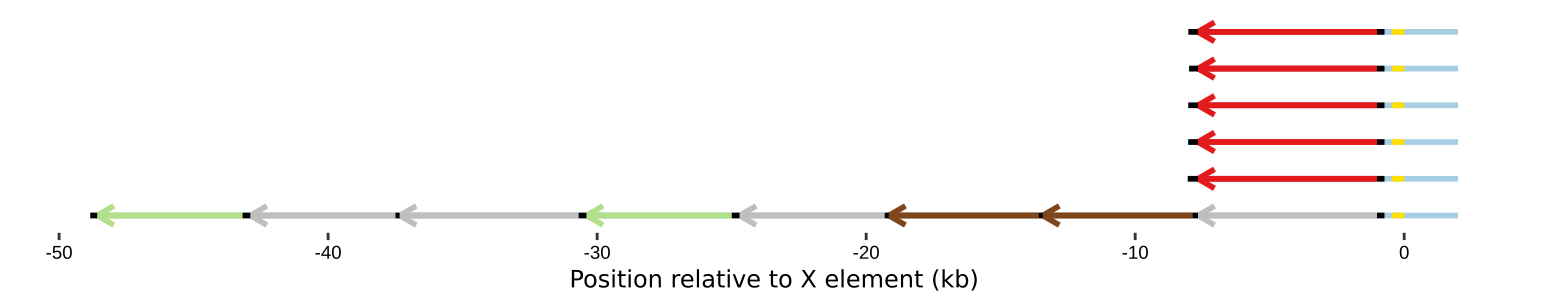

Supplement: Supplementary file 5 — Dataset EV2 [file 44319_2026_717_MOESM5_ESM.zip › Dataset EV2/rad59/Chr_XIV.left.png]

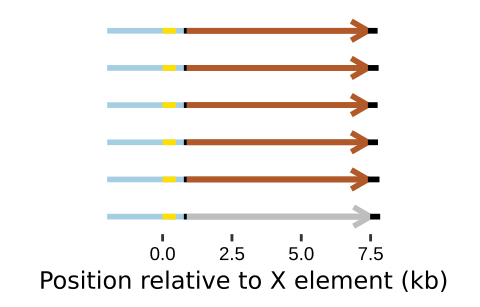

Supplement: Supplementary file 5 — Dataset EV2 [file 44319_2026_717_MOESM5_ESM.zip › Dataset EV2/rad59/Chr_XIV.right.png]

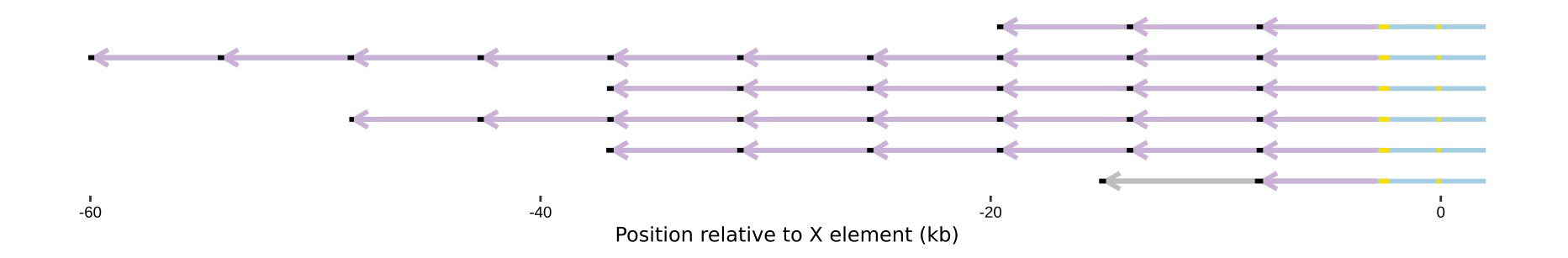

Supplement: Supplementary file 5 — Dataset EV2 [file 44319_2026_717_MOESM5_ESM.zip › Dataset EV2/rad59/Chr_VIII.left.png]

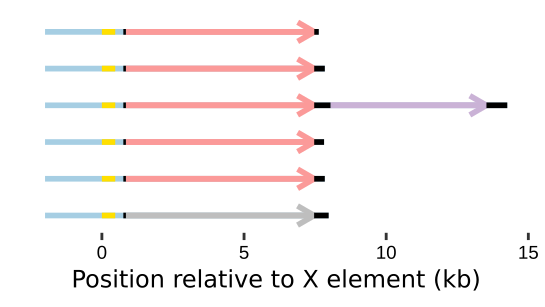

Supplement: Supplementary file 5 — Dataset EV2 [file 44319_2026_717_MOESM5_ESM.zip › Dataset EV2/pol32/Chr_II.right.png]

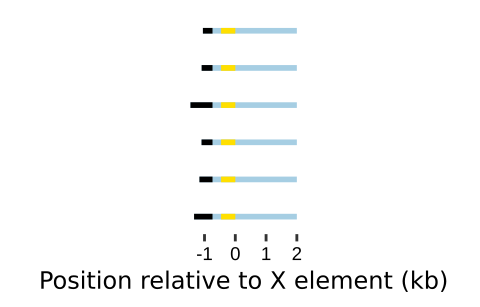

Supplement: Supplementary file 5 — Dataset EV2 [file 44319_2026_717_MOESM5_ESM.zip › Dataset EV2/pol32/Chr_XI.left.png]

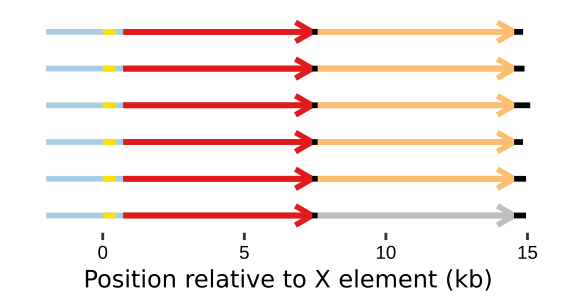

Supplement: Supplementary file 5 — Dataset EV2 [file 44319_2026_717_MOESM5_ESM.zip › Dataset EV2/pol32/Chr_XIII.right.png]

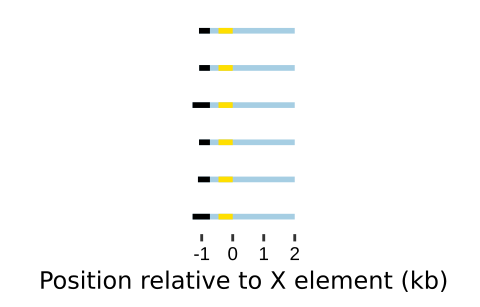

Supplement: Supplementary file 5 — Dataset EV2 [file 44319_2026_717_MOESM5_ESM.zip › Dataset EV2/pol32/Chr_XIII.left.png]

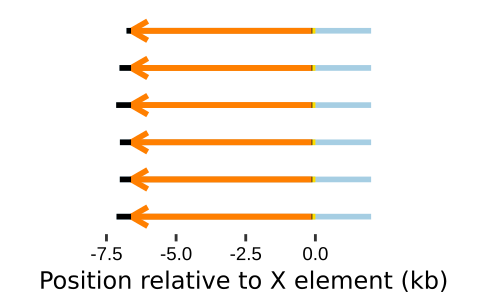

Supplement: Supplementary file 5 — Dataset EV2 [file 44319_2026_717_MOESM5_ESM.zip › Dataset EV2/pol32/Chr_V.left.png]

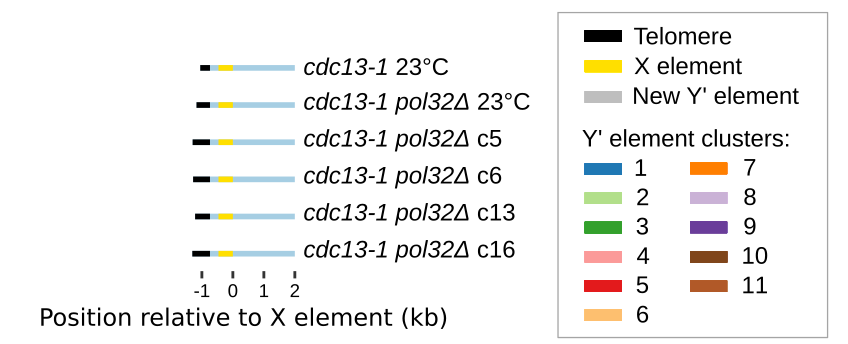

Supplement: Supplementary file 5 — Dataset EV2 [file 44319_2026_717_MOESM5_ESM.zip › Dataset EV2/pol32/Chr_I.left.png]

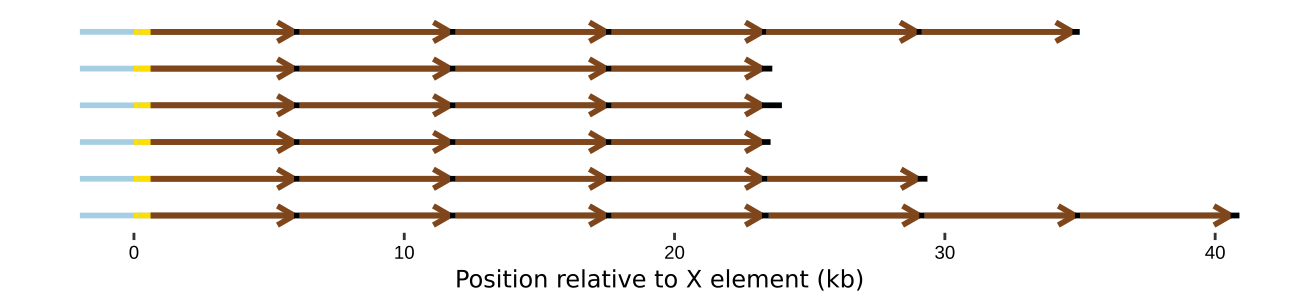

Supplement: Supplementary file 5 — Dataset EV2 [file 44319_2026_717_MOESM5_ESM.zip › Dataset EV2/pol32/Chr_XVI.right.png]

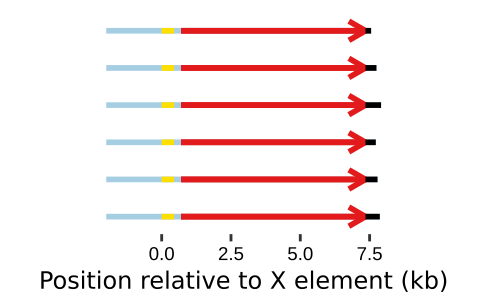

Supplement: Supplementary file 5 — Dataset EV2 [file 44319_2026_717_MOESM5_ESM.zip › Dataset EV2/pol32/Chr_XV.right.png]

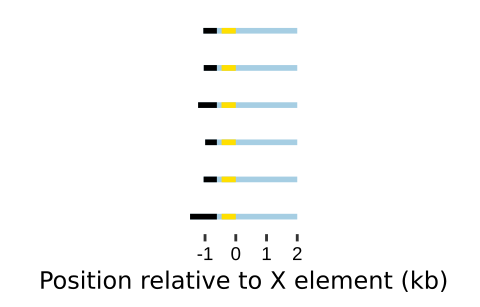

Supplement: Supplementary file 5 — Dataset EV2 [file 44319_2026_717_MOESM5_ESM.zip › Dataset EV2/pol32/Chr_VII.left.png]

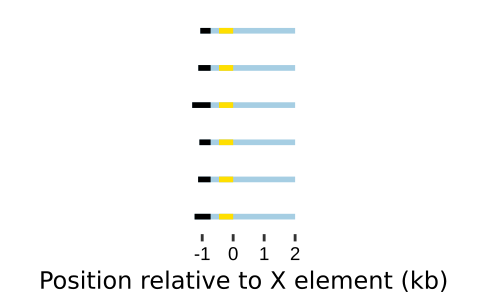

Supplement: Supplementary file 5 — Dataset EV2 [file 44319_2026_717_MOESM5_ESM.zip › Dataset EV2/pol32/Chr_XV.left.png]

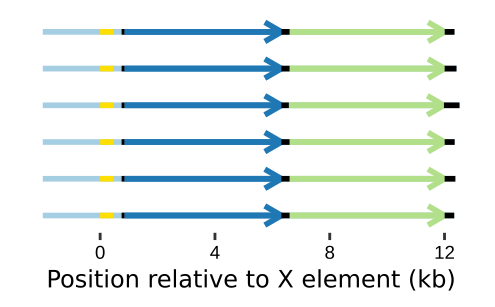

Supplement: Supplementary file 5 — Dataset EV2 [file 44319_2026_717_MOESM5_ESM.zip › Dataset EV2/pol32/Chr_I.right.png]

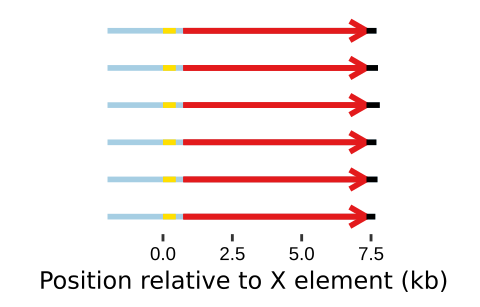

Supplement: Supplementary file 5 — Dataset EV2 [file 44319_2026_717_MOESM5_ESM.zip › Dataset EV2/pol32/Chr_V.right.png]

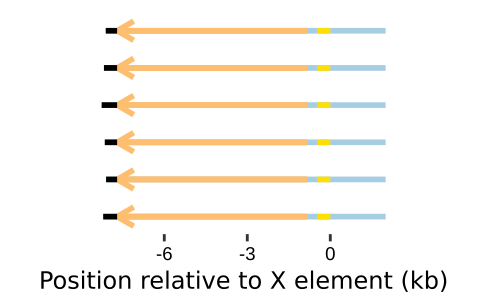

Supplement: Supplementary file 5 — Dataset EV2 [file 44319_2026_717_MOESM5_ESM.zip › Dataset EV2/pol32/Chr_IX.left.png]

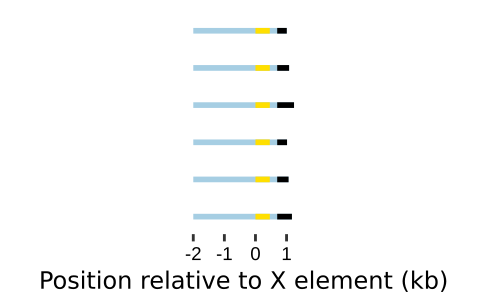

Supplement: Supplementary file 5 — Dataset EV2 [file 44319_2026_717_MOESM5_ESM.zip › Dataset EV2/pol32/Chr_XI.right.png]

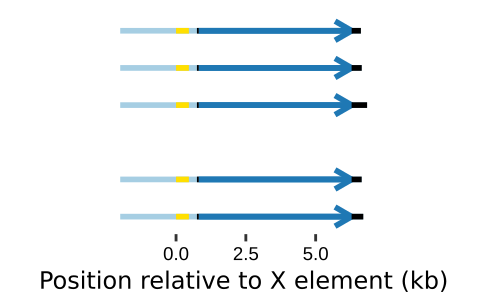

Supplement: Supplementary file 5 — Dataset EV2 [file 44319_2026_717_MOESM5_ESM.zip › Dataset EV2/pol32/Chr_III.right.png]

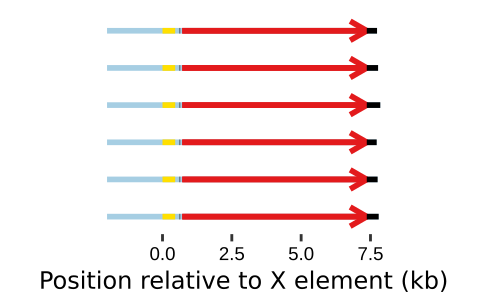

Supplement: Supplementary file 5 — Dataset EV2 [file 44319_2026_717_MOESM5_ESM.zip › Dataset EV2/pol32/Chr_VII.right.png]

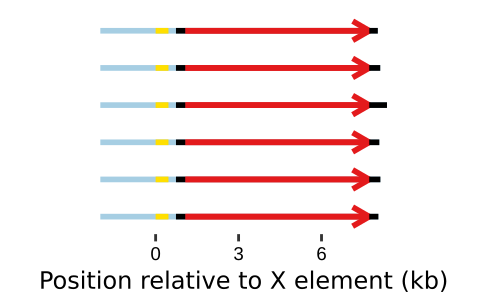

Supplement: Supplementary file 5 — Dataset EV2 [file 44319_2026_717_MOESM5_ESM.zip › Dataset EV2/pol32/Chr_IV.right.png]

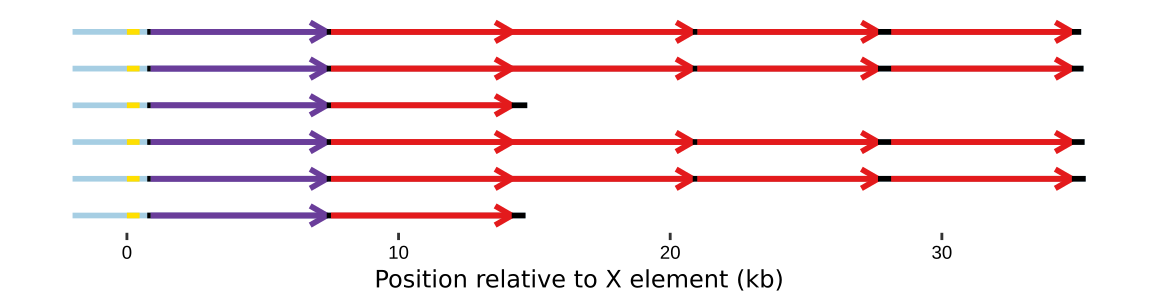

Supplement: Supplementary file 5 — Dataset EV2 [file 44319_2026_717_MOESM5_ESM.zip › Dataset EV2/pol32/Chr_XII.right.png]

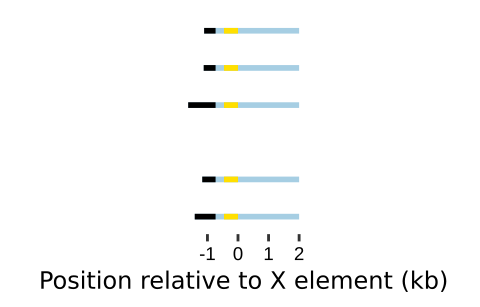

Supplement: Supplementary file 5 — Dataset EV2 [file 44319_2026_717_MOESM5_ESM.zip › Dataset EV2/pol32/Chr_III.left.png]

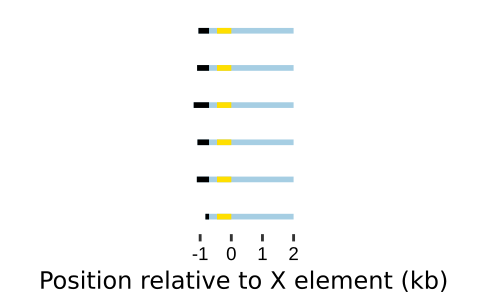

Supplement: Supplementary file 5 — Dataset EV2 [file 44319_2026_717_MOESM5_ESM.zip › Dataset EV2/pol32/Chr_IV.left.png]

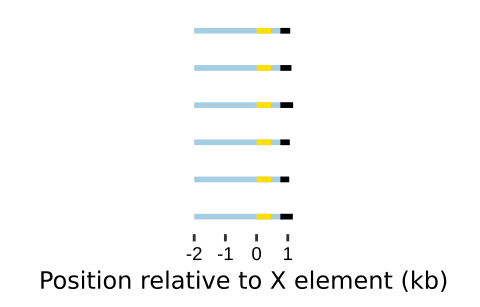

Supplement: Supplementary file 5 — Dataset EV2 [file 44319_2026_717_MOESM5_ESM.zip › Dataset EV2/pol32/Chr_X.right.png]

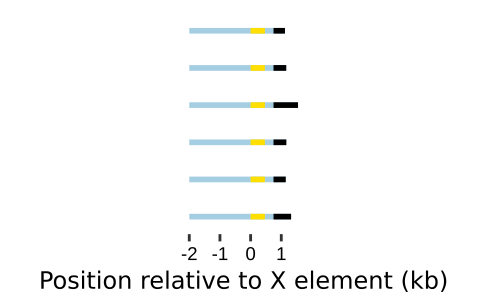

Supplement: Supplementary file 5 — Dataset EV2 [file 44319_2026_717_MOESM5_ESM.zip › Dataset EV2/pol32/Chr_IX.right.png]

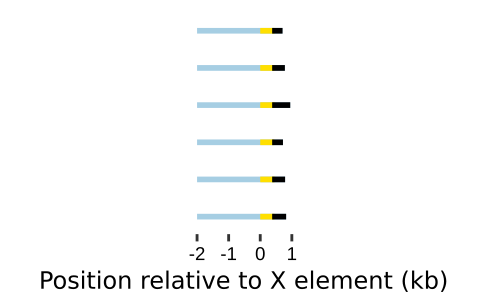

Supplement: Supplementary file 5 — Dataset EV2 [file 44319_2026_717_MOESM5_ESM.zip › Dataset EV2/pol32/Chr_VI.right.png]

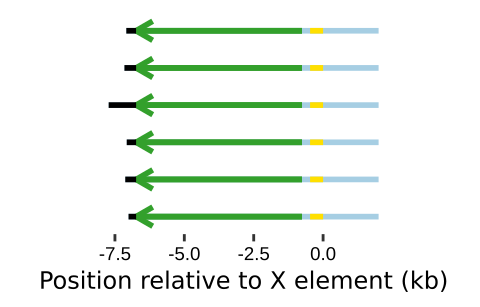

Supplement: Supplementary file 5 — Dataset EV2 [file 44319_2026_717_MOESM5_ESM.zip › Dataset EV2/pol32/Chr_II.left.png]

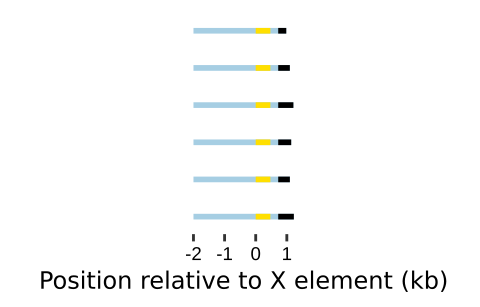

Supplement: Supplementary file 5 — Dataset EV2 [file 44319_2026_717_MOESM5_ESM.zip › Dataset EV2/pol32/Chr_VIII.right.png]

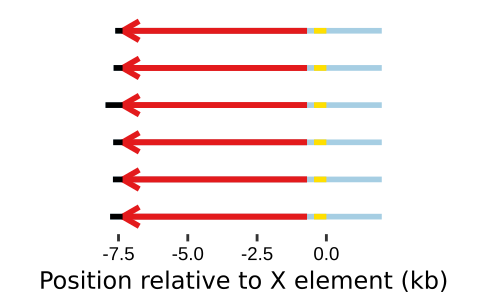

Supplement: Supplementary file 5 — Dataset EV2 [file 44319_2026_717_MOESM5_ESM.zip › Dataset EV2/pol32/Chr_XVI.left.png]

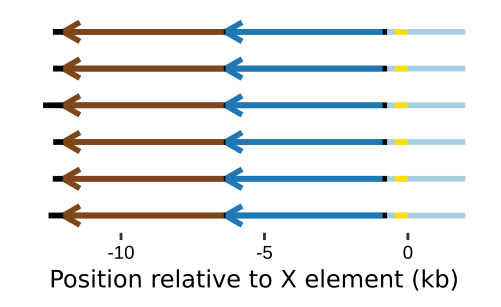

Supplement: Supplementary file 5 — Dataset EV2 [file 44319_2026_717_MOESM5_ESM.zip › Dataset EV2/pol32/Chr_XII.left.png]

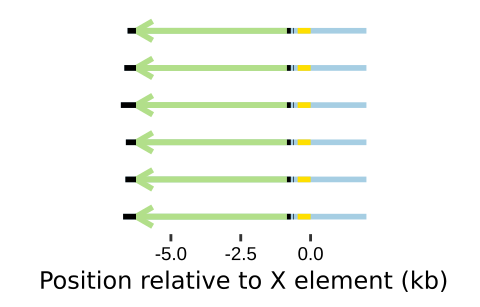

Supplement: Supplementary file 5 — Dataset EV2 [file 44319_2026_717_MOESM5_ESM.zip › Dataset EV2/pol32/Chr_VI.left.png]

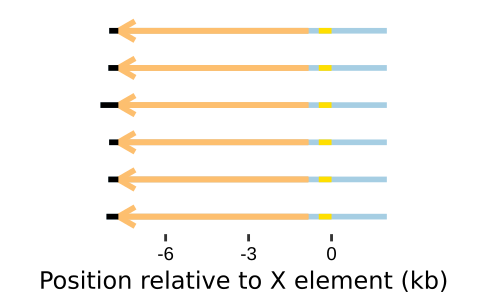

Supplement: Supplementary file 5 — Dataset EV2 [file 44319_2026_717_MOESM5_ESM.zip › Dataset EV2/pol32/Chr_X.left.png]

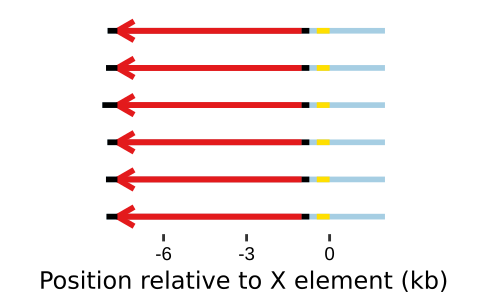

Supplement: Supplementary file 5 — Dataset EV2 [file 44319_2026_717_MOESM5_ESM.zip › Dataset EV2/pol32/Chr_XIV.left.png]

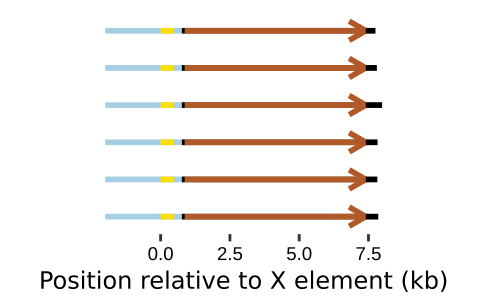

Supplement: Supplementary file 5 — Dataset EV2 [file 44319_2026_717_MOESM5_ESM.zip › Dataset EV2/pol32/Chr_XIV.right.png]

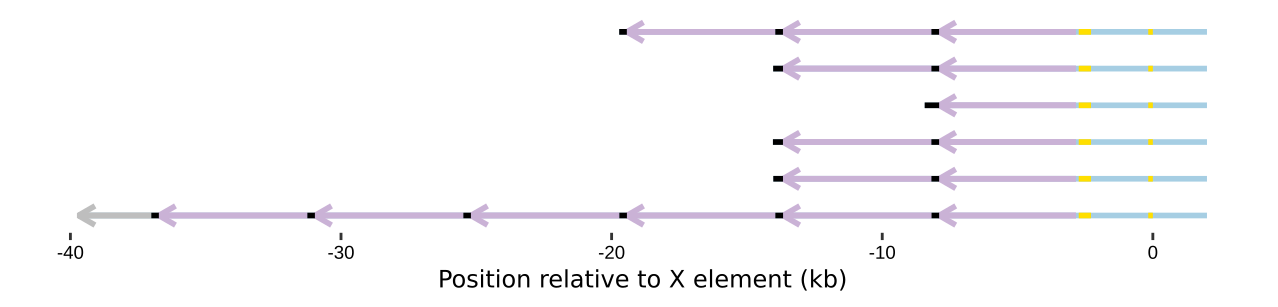

Supplement: Supplementary file 5 — Dataset EV2 [file 44319_2026_717_MOESM5_ESM.zip › Dataset EV2/pol32/Chr_VIII.left.png]

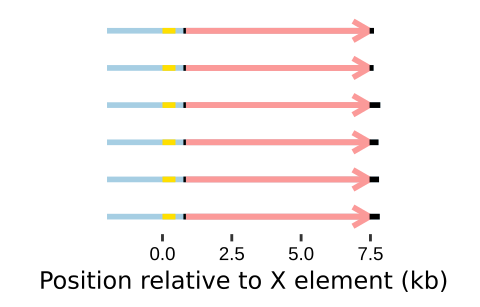

Supplement: Supplementary file 5 — Dataset EV2 [file 44319_2026_717_MOESM5_ESM.zip › Dataset EV2/rad51_rad59/Chr_II.right.png]

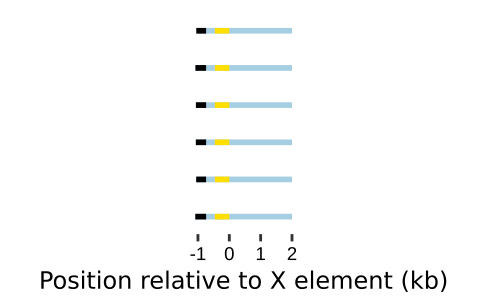

Supplement: Supplementary file 5 — Dataset EV2 [file 44319_2026_717_MOESM5_ESM.zip › Dataset EV2/rad51_rad59/Chr_XI.left.png]

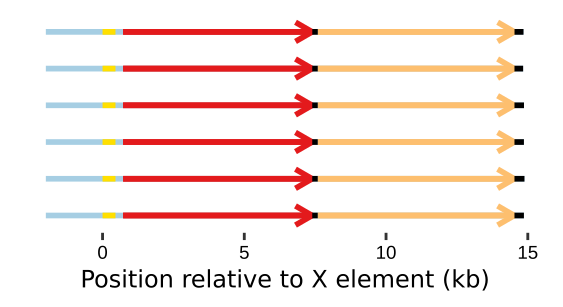

Supplement: Supplementary file 5 — Dataset EV2 [file 44319_2026_717_MOESM5_ESM.zip › Dataset EV2/rad51_rad59/Chr_XIII.right.png]

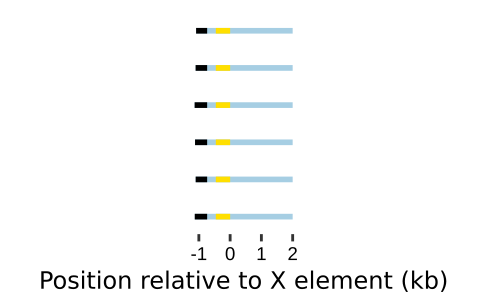

Supplement: Supplementary file 5 — Dataset EV2 [file 44319_2026_717_MOESM5_ESM.zip › Dataset EV2/rad51_rad59/Chr_XIII.left.png]
