# Supplementary figures and images for: Transient telomere uncapping triggers telomeric and subtelomeric rearrangements (part 3 of 3)
Source: EMBO Rep. 2026 Feb 17;27(6):1607–31. doi: 10.1038/s44319-026-00717-4 (PMC13022453; doi:10.1038/s44319-026-00717-4)

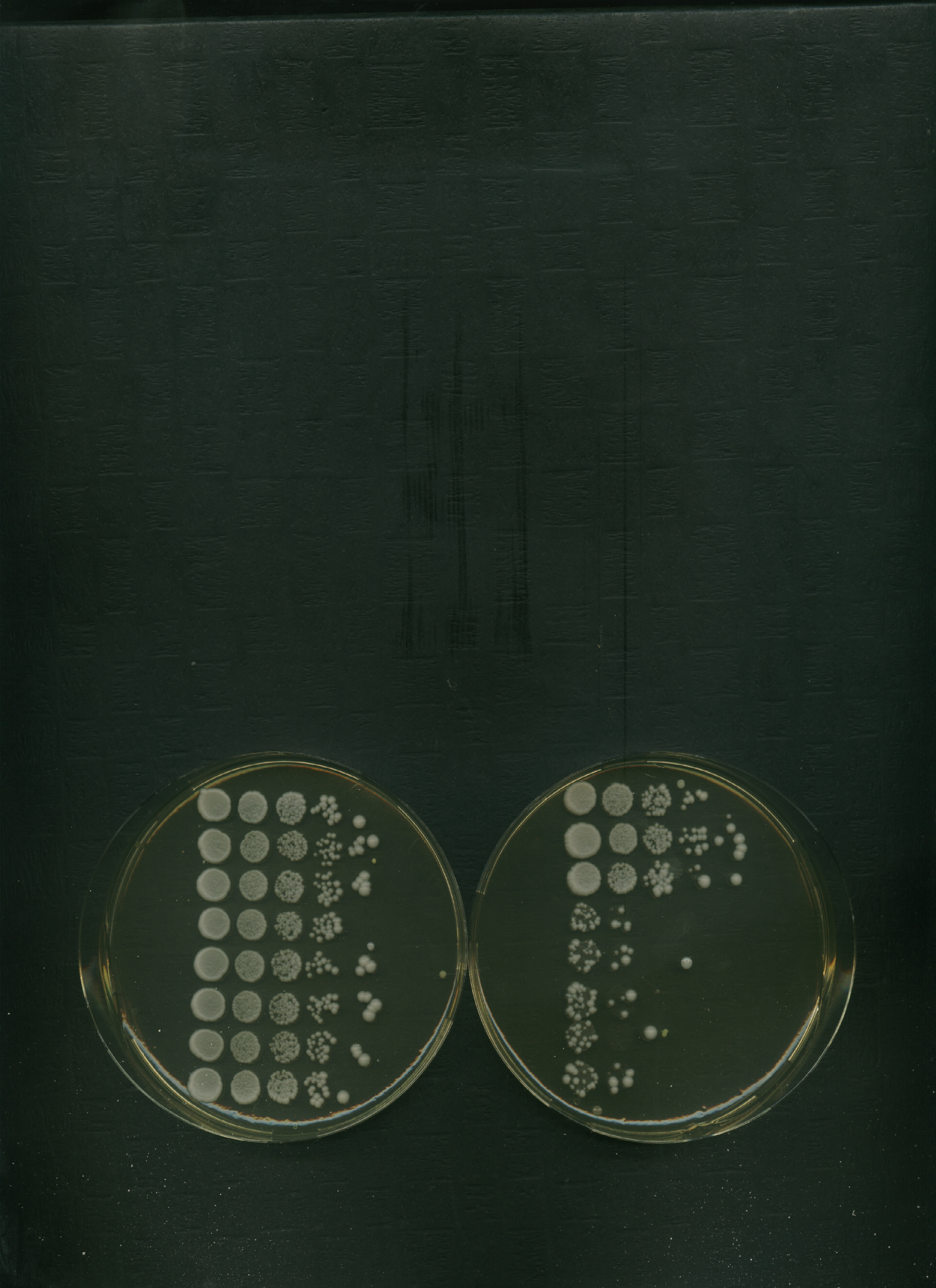

Supplement: Supplementary file 9 — Source data Fig. 6 [file 44319_2026_717_MOESM9_ESM.zip › SourceData_Fig6/6A/rawdata_fig6A.jpg]

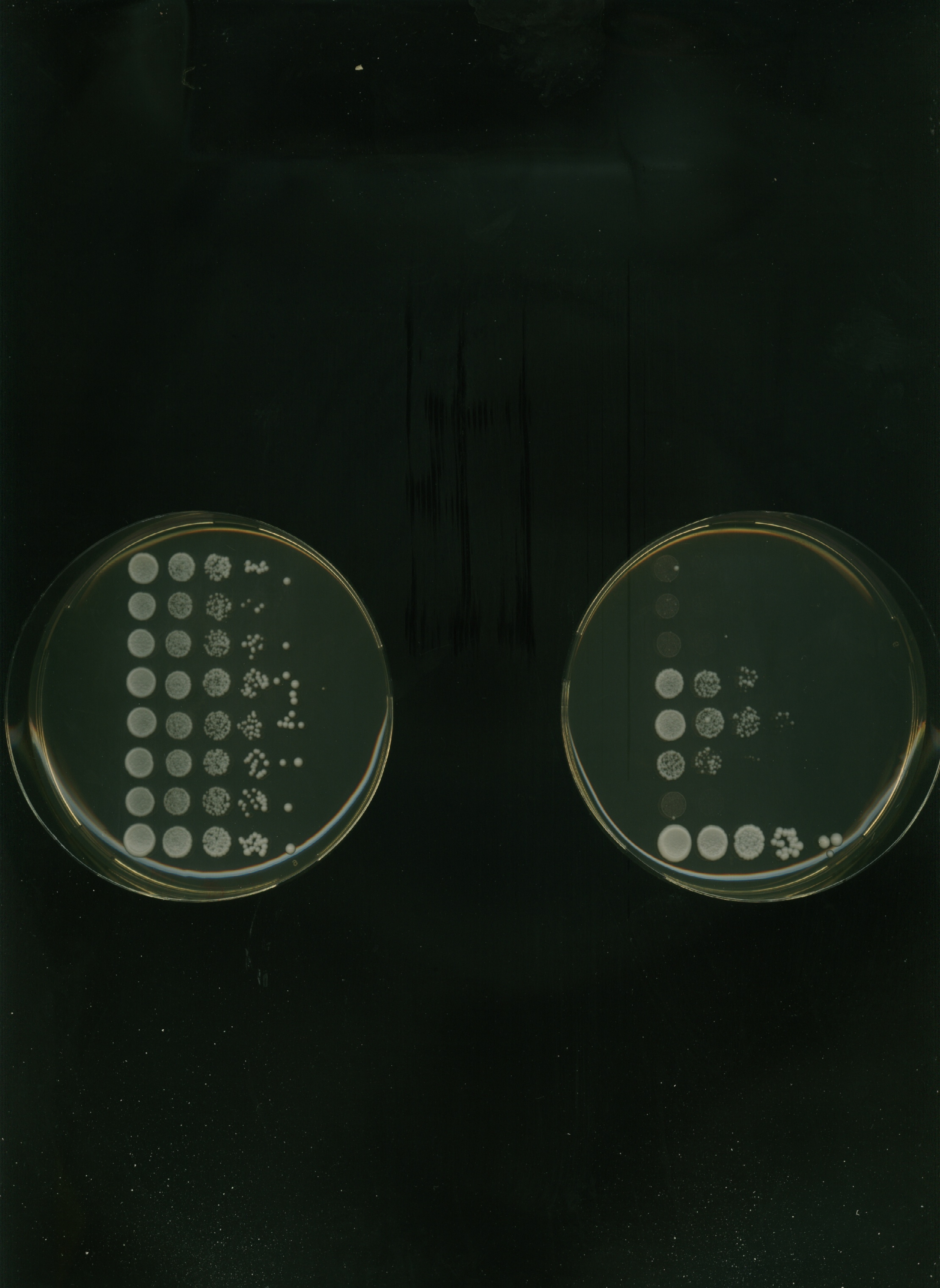

Supplement: Supplementary file 9 — Source data Fig. 6 [file 44319_2026_717_MOESM9_ESM.zip › SourceData_Fig6/6B/rawdata_fig6B.jpg]

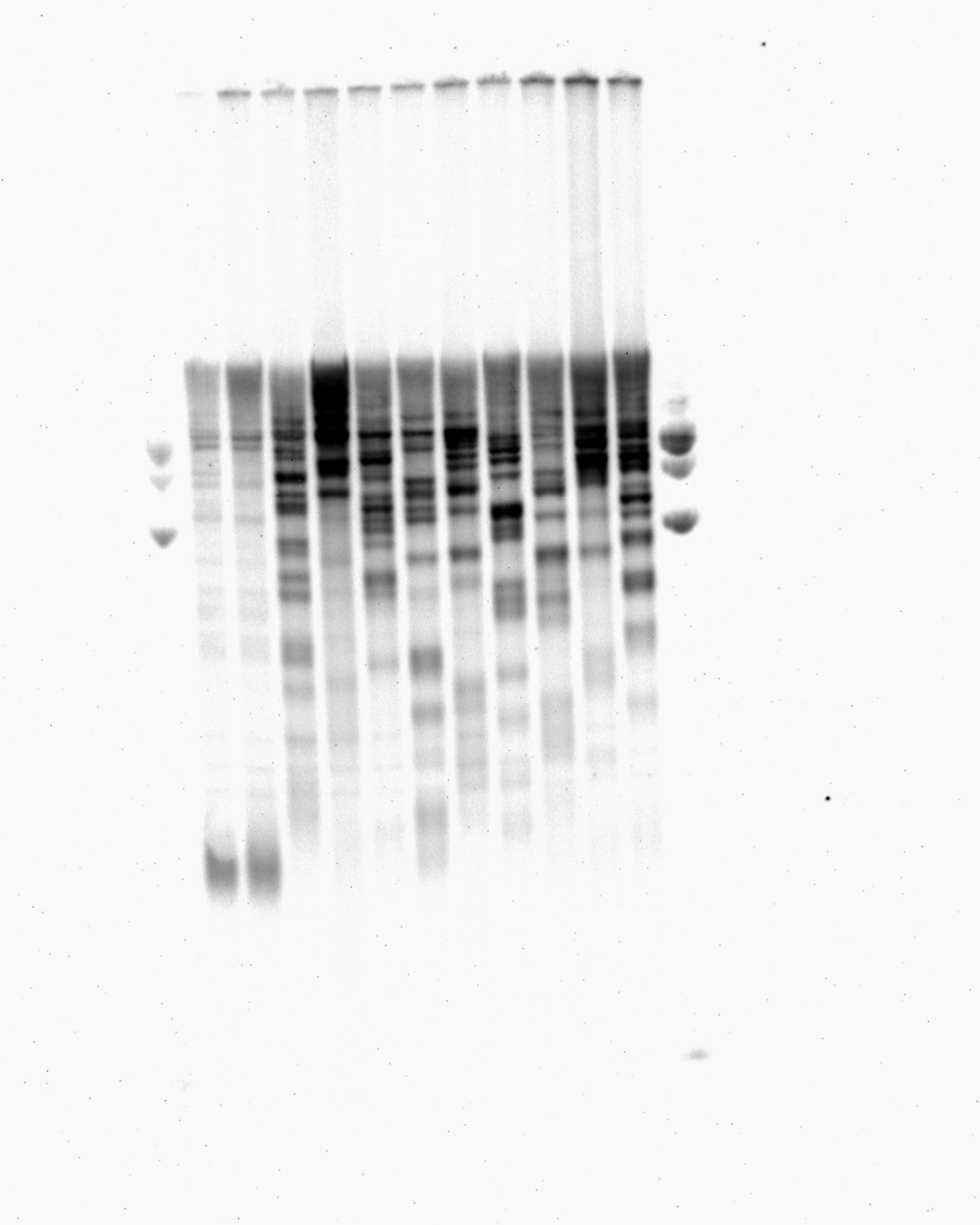

Supplement: Supplementary file 9 — Source data Fig. 6 [file 44319_2026_717_MOESM9_ESM.zip › SourceData_Fig6/6C/rawdata_fig6C.jpg]
