# Supplementary material for: Transient telomere uncapping triggers telomeric and subtelomeric rearrangements
Source: EMBO Rep. 2026 Feb 17;27(6):1607–31. doi: 10.1038/s44319-026-00717-4 (PMC13022453; doi:10.1038/s44319-026-00717-4)
Supplement: Supplementary file 10 — Expanded View Figures [file 44319_2026_717_MOESM10_ESM.pdf]

Expanded View Figures

Figure EV1. Heterogeneous genome rearrangements at telomeres and subtelomeres.

(**A**) TRF Southern blot analysis of 16 *cdc13-1* survivor clones in addition to those shown in Fig. 2B. The control lane for *cdc13-1* 23 °C is taken from a different Southern blot. (**B**) Southern blot performed on the same membrane as in Fig. 1B, hybridized with a Y′-specific probe. The bottom panel shows ethidium bromide staining of the gel showing total DNA loading prior to transfer. (**C**) PFGE of four subclones of clones c2, c3 (also shown in Fig. 2C), and c5. The orange boxes indicate bands with distinct migration patterns in the four subclones. (**D**) PFGE of 7 *cdc13-1 cdc5-ad* transient uncapping survivor clones, as well as a *cdc13-1* control strain grown constantly at 23 °C (same control as in Fig. 2A). Compared to the control strain, six out of seven survivor clones exhibited apparent chromosome size shifts, marked by orange arrows. (**E**) Same as (**C**) in a *cdc13-1 cdc5-ad* strain.

© The Author(s)

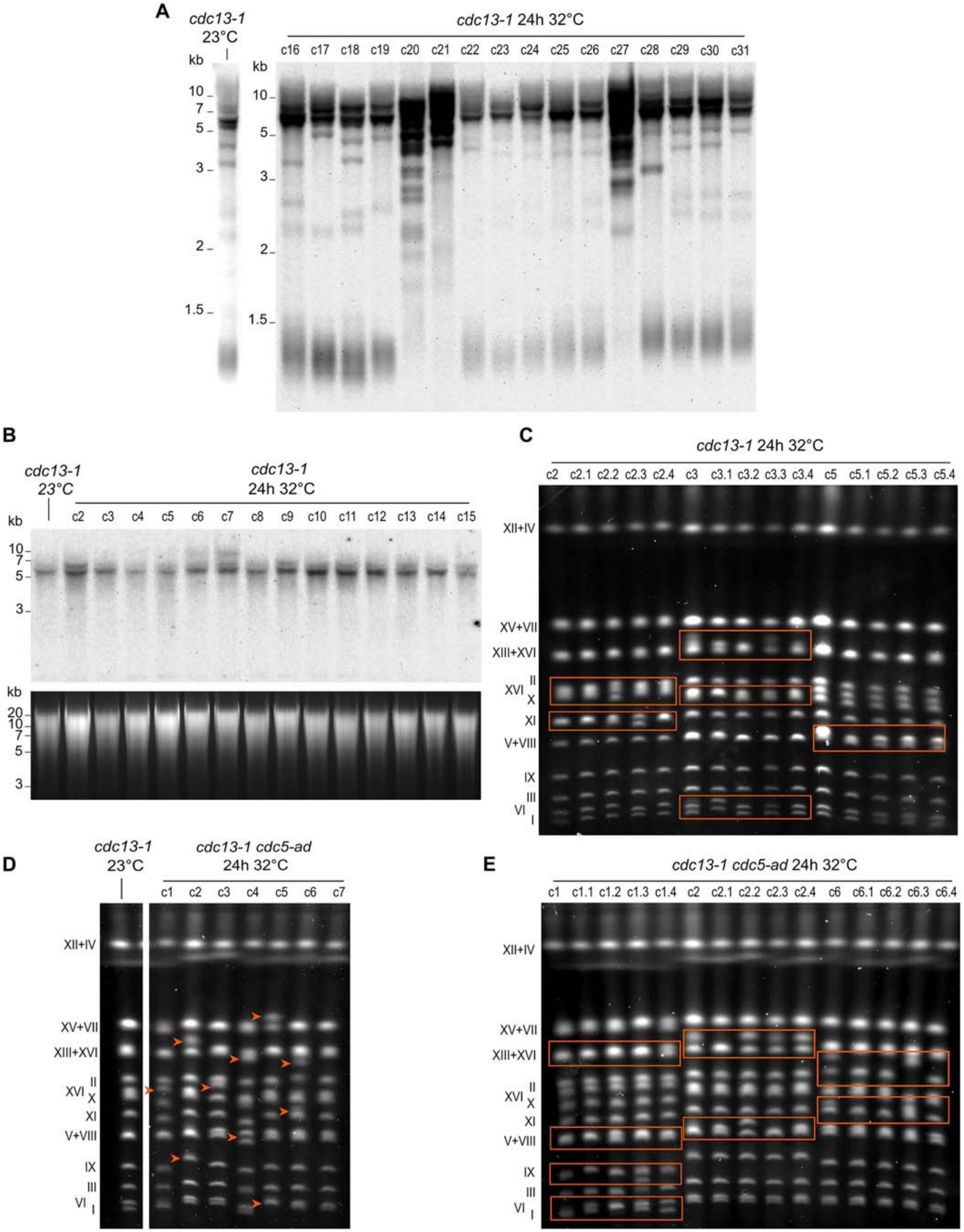
A
cdc13-1 23°C
cdc13-1 24h 32°C
c16 c17 c18 c19 c20 c21 c22 c23 c24 c25 c26 c27 c28 c29 c30 c31
kb
10 7 5 3 2 1.5
B
cdc13-1 23°C
cdc13-1 24h 32°C
c2 c3 c4 c5 c6 c7 c8 c9 c10 c11 c12 c13 c14 c15
20 10 7 5 3
C
cdc13-1 24h 32°C
c2 c2.1 c2.2 c2.3 c2.4 c3 c3.1 c3.2 c3.3 c3.4 c5 c5.1 c5.2 c5.3 c5.4
XII+IV
XV+VII
XIII+XVI
II
XVI X
XI
V+VIII
IX
III
VI I
D
cdc13-1 23°C
cdc13-1 cdc5-ad 24h 32°C
c1 c2 c3 c4 c5 c6 c7
E
cdc13-1 cdc5-ad 24h 32°C
c1 c1.1 c1.2 c1.3 c1.4 c2 c2.1 c2.2 c2.3 c2.4 c6 c6.1 c6.2 c6.3 c6.4

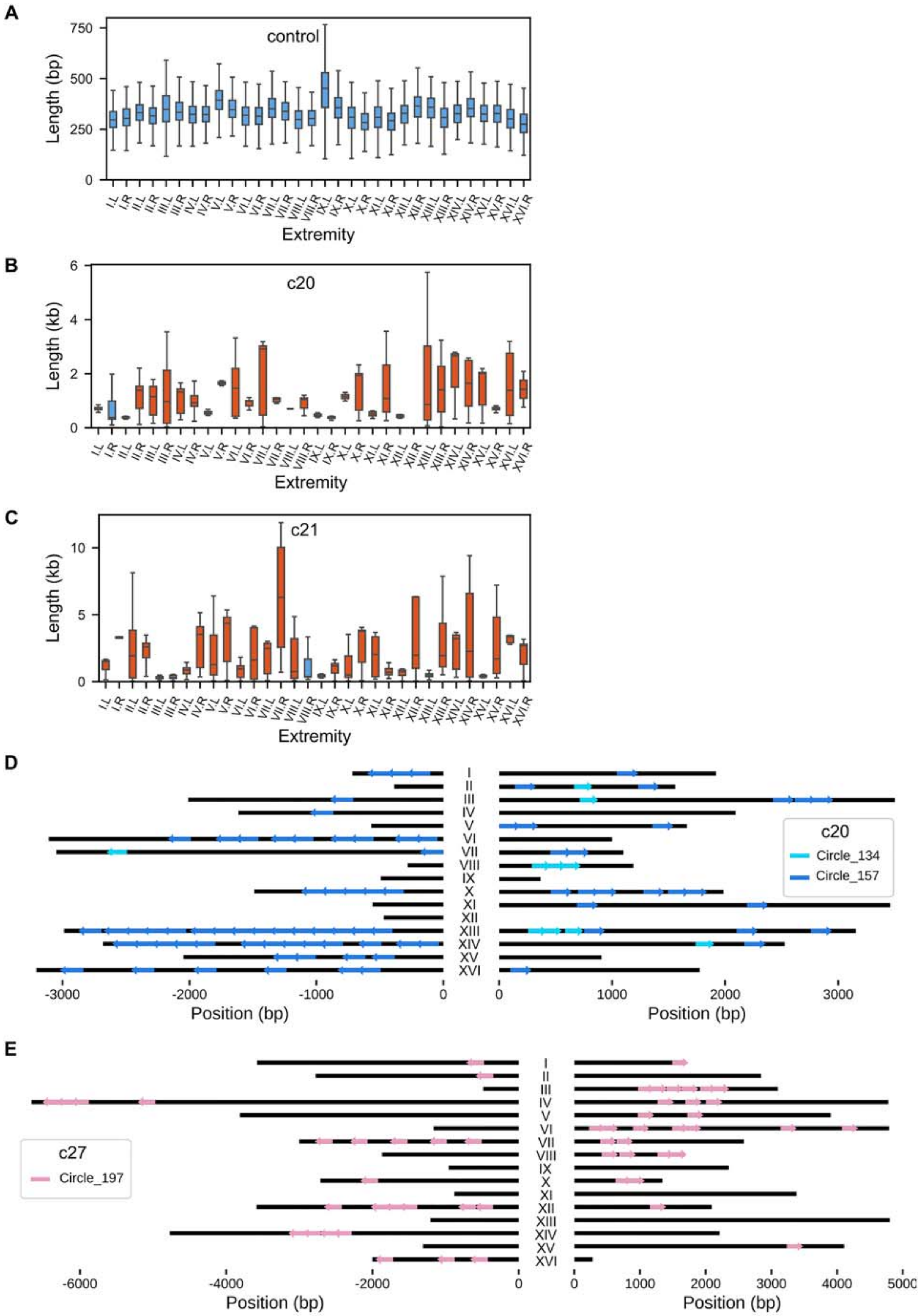
A
control
Length (bp)
Extremity
B
c20
Length (kb)
Extremity
C
c21
Length (kb)
Extremity
D
c20
Circle_134
Circle_157
Position (bp)
Position (bp)
E
c27
Circle_197
Position (bp)
Position (bp)

Figure EV2. Telomeres are elongated heterogeneously in T-II-L survivors and contain large perfect tandem repeats.

(**A**) Boxplots representing the telomere length distribution at each chromosome extremity of the control *cdc13-1* strain grown at 23 °C. Same data as in Fig. 4C left with a different scale on the y-axis. The boxplots follow the same specifications as in Fig. 4C. (**B**, **C**) Same as (**A**) for type-II-like survivor clones c20 and c21. The blue boxplots correspond to distributions with a mean telomere length <500 bp. The boxplots follow the same specifications as in Fig. 4C. The number of reads used to draw the boxplots are comprised between $n = 3$ and $n = 31$ for (**B**), except for XII.R and VIII.L, which have $n = 0$ (no boxplot) and $n = 1$ (single line), respectively, and are comprised between $n = 4$ and $n = 32$ for (**C**). (**D**, **E**) Visualization of putative circle-originating sequences detected in the left and right telomeres of the 16 chromosomes from clones c20 and c27. The circle number corresponds to the length of the circle sequence. Black horizontal lines represent the assembled telomeric sequences.

© The Author(s)

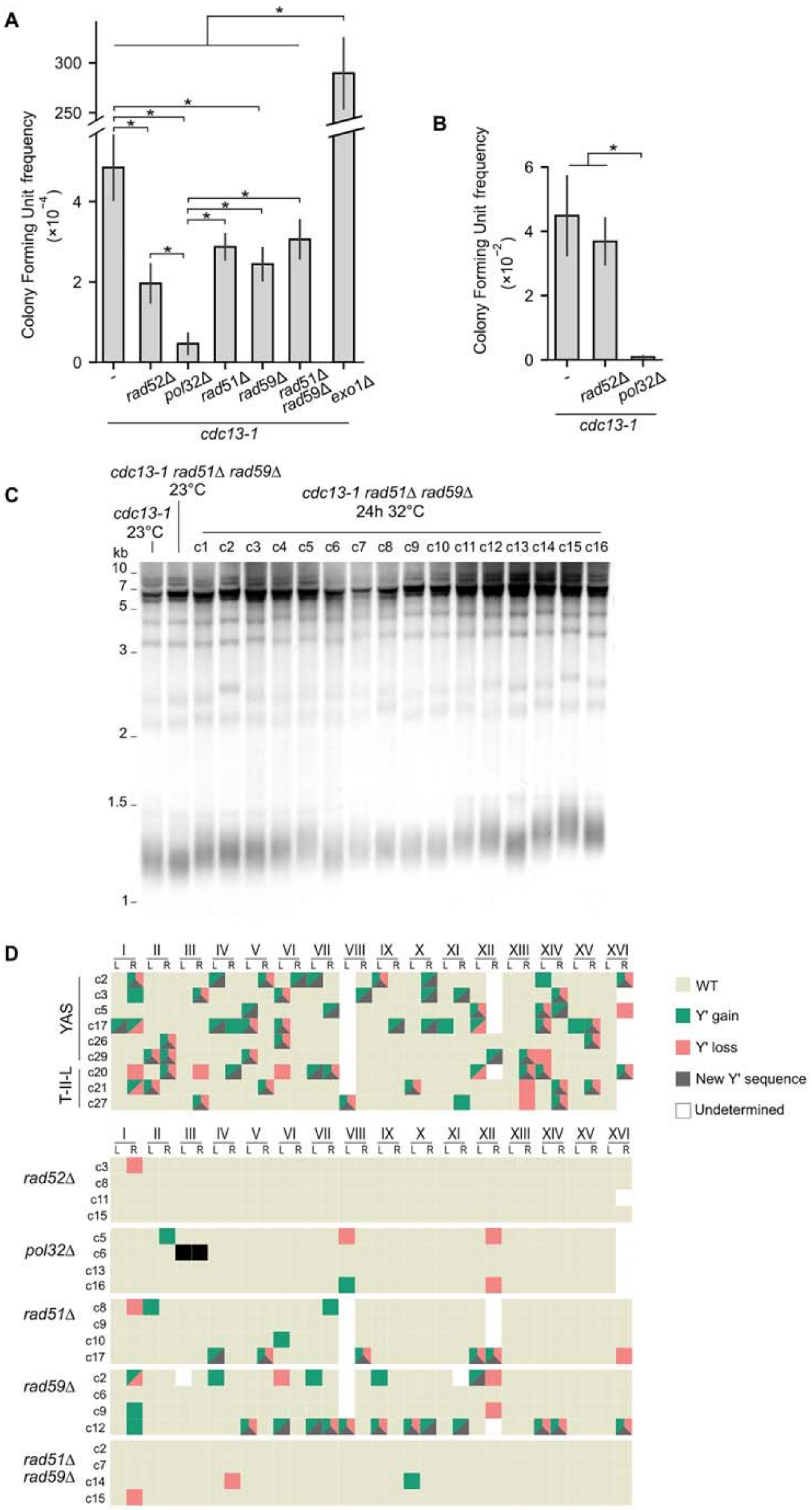
A
Colony Forming Unit frequency (×10^-4)
300
250
4
2
0
-
rad52Δ
pol32Δ
rad51Δ
rad59Δ
rad51Δ rad59Δ
exo1Δ
cdc13-1
B
Colony Forming Unit frequency (×10^-2)
6
4
2
0
-
rad52Δ
pol32Δ
cdc13-1
C
cdc13-1 rad51Δ rad59Δ 23°C
cdc13-1 23°C
cdc13-1 rad51Δ rad59Δ 24h 32°C
c1 c2 c3 c4 c5 c6 c7 c8 c9 c10 c11 c12 c13 c14 c15 c16
kb
10
7
5
3
2
1.5
1
D
I II III IV V VI VII VIII IX X XI XII XIII XIV XV XVI
L R
YAS
c2 c3 c5 c17 c26 c29
T-II-L
c20 c21 c27
WT
Y' gain
Y' loss
New Y' sequence
Undetermined
rad52Δ
c3 c8 c11 c15
pol32Δ
c5 c6 c13 c16
rad51Δ
c8 c9 c10 c17
rad59Δ
c2 c6 c9 c12
rad51Δ rad59Δ
c2 c7 c14 c15

 © The Author(s)

Figure EV3. Survival and alterations in single and double mutants in the *cdc13-1* background.

(**A**) Colony formation frequency after 24 h of transient telomere uncapping and return to 23 °C in the indicated mutants. A control plate with the *cdc13-1* strain was kept only at 23 °C for normalization. The number of independent samples is $n = 7$, 4, 4, 4, 5, 3, and 3, following the order of the x-axis. The error bars correspond to the standard error of the mean. Asterisks indicate statistically significant differences (Student's *t*-test: for *exo1Δ* against the other strains from left to right, p value $= 9.0 \times 10^{-7}$, 2.0×10^{-4}, 1.9×10^{-4}, 2.0×10^{-4}, 3.2×10^{-5}, and 1.3×10^{-3}; for other asterisks, from top to bottom, p value $= 0.040$, 0.032, 0.0032, 0.0036, 0.0062, 0.0011, and 0.034). (**B**) Same as (**A**) for the indicated strains, but following 12 h of transient telomere uncapping, with $n = 4$, 6, and 5, following the order of the x-axis. Student's t-test, *: p value $= 0.0048$ for WT against *pol32Δ* and p value $= 0.0015$ for *rad52Δ* against *pol32Δ*. (**C**) TRF Southern blots of *cdc13-1 rad51Δ rad59Δ* survivor colonies. A culture from the same strain that was maintained at 23 °C was used as a control. (**D**) Map of chromosome extremity alterations further detailing modified Y′ structures from Figs. 3B and 5E. In this map, a substitution is simultaneously a loss and a gain of the Y′ element.

© The Author(s)

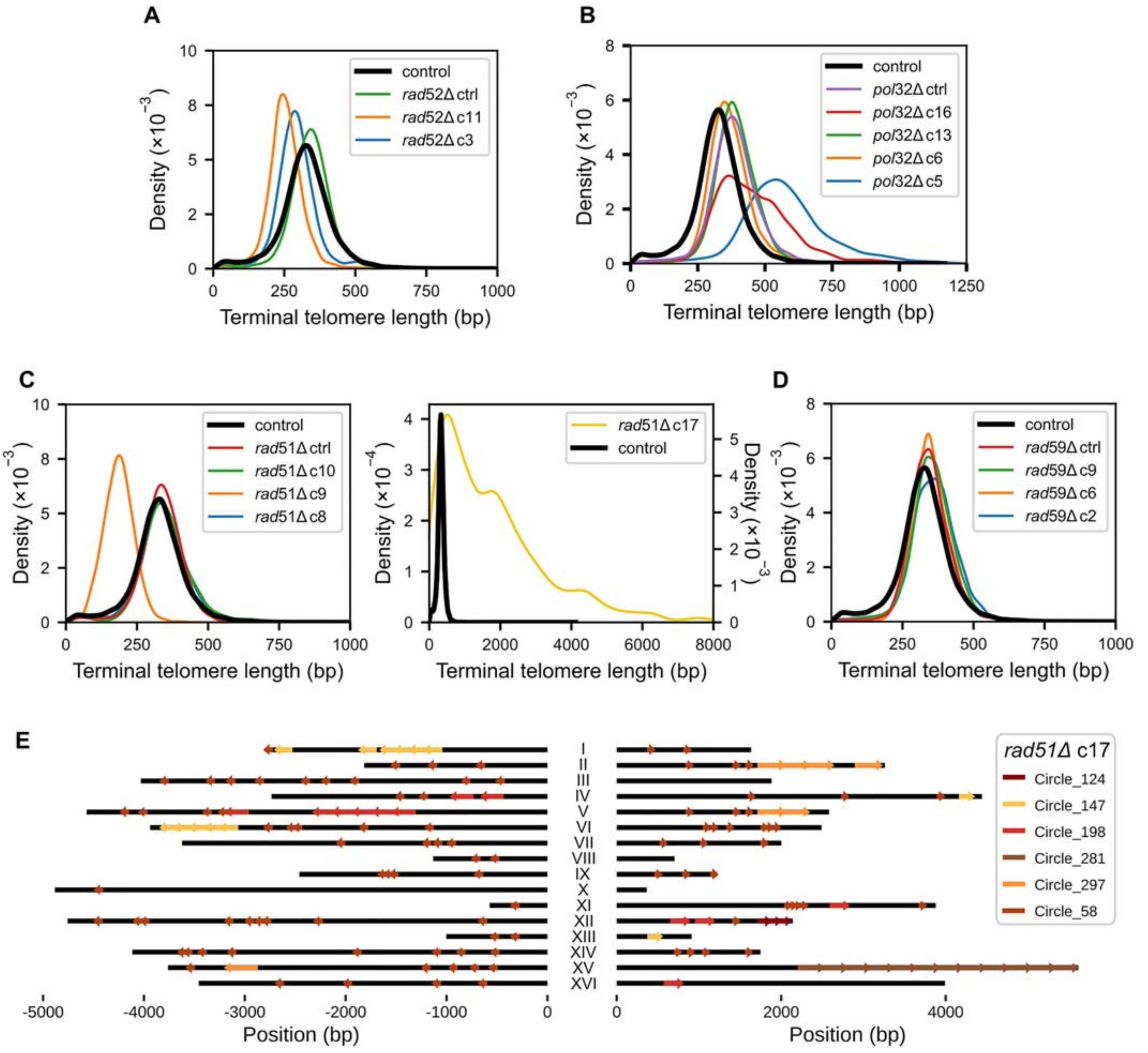

Figure EV4. Telomere length distributions of transient uncapping survivors in the *rad52*Δ, *pol32*Δ, *rad51*Δ, and *rad59*Δ mutants.

(**A–D**) Telomere length distributions of the indicated strains derived from nanopore sequencing reads, compared to the control (black line). In (**C**), the left plot displays the telomere length distribution of *rad51Δ* YAS survivors, while the right plot displays the telomere length distribution of the *rad51Δ* type-II-like survivor (c17), with a different scale for the x-axis. (**E**) Visualization of putative circle-originating sequences detected in the left and right telomeres of the 16 chromosomes of the *rad51Δ* T-II-L clone c17. The circle number corresponds to the length of the circle sequence. Black horizontal lines represent the assembled telomeric sequences.

 © The Author(s)

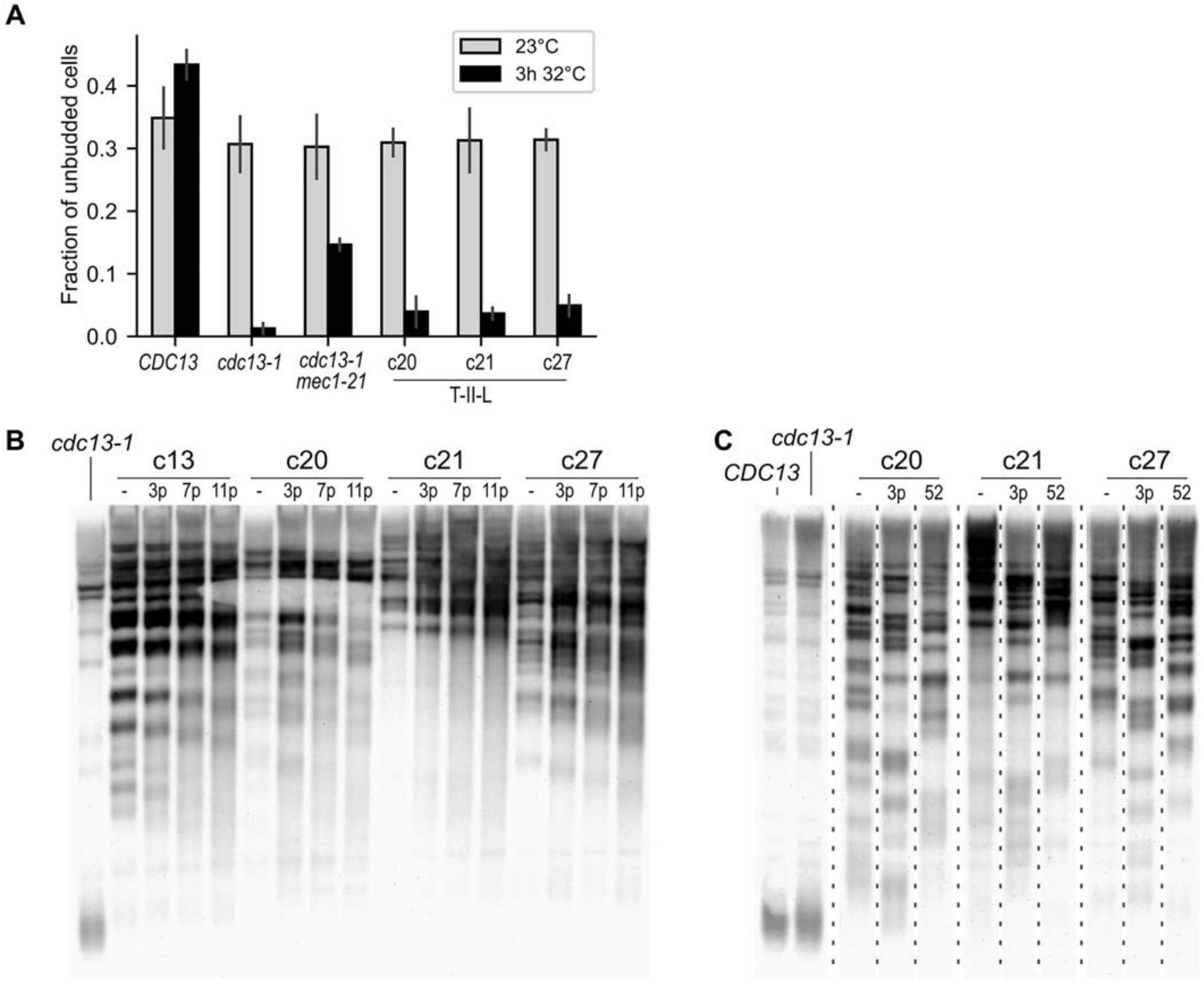

Figure EV5. Behavior of type-II-like survivors in response to a second telomere uncapping and through further passages.

(**A**) Fraction of unbudded cells in exponentially growing cultures at 23 °C or after 3 h at 32 °C. Unbudded cells are more abundant in the partially checkpoint-deficient mutant *mec1-21 cdc13-1* than in the control *cdc13-1* after 3 h at 32 °C. $n = 3$ independent cultures for each condition. The error bars correspond to the standard error of the mean. (**B**) TRF Southern blot of 4 type-II-like clones, passaged for an additional 3, 7, and 11 passages after initial subcloning. Contrary to Fig. 6C, restreaks were performed on bulk patches of cells instead of colonies. (**C**) Figure identical to Fig. 6C, except that the lanes have been digitally reordered to facilitate visual interpretation per clone. “52” indicates the *rad52Δ* derivative of the indicated strain.
